# Supplementary material for: A workplace organisational intervention to improve hospital nurses’ and physicians’ mental health: study protocol for the Magnet4Europe wait list cluster randomised controlled trial
Source: BMJ Open. 2022 Jul 28;12(7):e059159. doi: 10.1136/bmjopen-2021-059159 (PMC9341186; doi:10.1136/bmjopen-2021-059159)
Supplement: Supplementary data [file bmjopen-2021-059159supp002.pdf]

Supplementary File 2 – Participant information sheet and consent form for quantitative (p. 2-3) and qualitative data collection (p.4-5) (English version – Example)

Sermeus W, *et al. BMJ Open* 2022; 12:e059159. doi: 10.1136/bmjopen-2021-059159

Magnet4Europe - Improving Mental Health and Wellbeing in the Health Care Workplace

Dear colleague,
you have been invited to take part in a research study that aims to improve mental health and wellbeing among health professionals in Europe. The project is funded under the European Union's Horizon 2020 Research and Innovation programme from 2020 to 2023 (Grant Agreement 848031). The study will be conducted in compliance with your country's ethics guidelines for scientific studies on individuals. Your participation will neither involve costs nor reimbursements. You can withdraw from the study at any time, without giving a reason. Before you decide whether to take part it is important for you to understand why the research is being done and what it will involve. Please take your time to read the following information carefully and to decide whether you wish to take part or not. Ask us if there is anything that is not clear or if you would like to receive more information. For questions, please reach out to the Magnet4Europe project coordinator prof. dr. Walter Sermeus at walter.sermeus@kuleuven.be.

What is the purpose of the study?

Mental health and wellbeing are among the highest priorities of the public health agenda in the European Union (EU). A large-scale European study of hospital work conditions and associated nurse and patient outcomes revealed high rates of job dissatisfaction and burnout, with burnout rates varying between 10% and 78%. Physician burnout rates ranging from 25% to 60% have been reported, varying across organizations and medical specialties. Magnet4Europe aims to redesign clinical environments in sixty hospitals in five European countries (Belgium, United Kingdom, Germany, Ireland, and Sweden) to promote the mental health and wellbeing of health professionals. Magnet4Europe will implement an evidence-based intervention based on the successful Magnet Recognition Program®, a voluntary hospital designation for nursing care excellence by the American Nurses Credentialing Center. Countless studies have shown that Magnet-recognized hospitals have lower health professional burnout and safer patient care suggesting that the Magnet journey is an intervention that results in positive changes Magnet4Europe seeks to achieve.

How does this research study work?

This research study will be carried out between January 2020 and December 2023 in adult inpatient units. The intervention itself will officially start in September 2020 in participating European hospitals and will target the five overarching principles of the Magnet® concept: structural empowerment of clinical staff, transformational leadership, exemplary and evidence-based professional practice, new knowledge, innovations, and improvements, and empirical outcomes. These principles will serve as a continuous feedback loop as to whether organizational changes are producing the intended outcomes for staff and patients. The Magnet® blueprint provides definitions of the principles and gives examples of evidence-based indicators that reflect progress toward achieving them. To facilitate the implementation process, each participating European hospital receives support by one to one twinning with an experienced Magnet recognized hospital including annual on-site visits and by the creation of a critical mass of participating hospitals drawing public interest and promoting innovation, and replication. Before the intervention period officially starts, eligible participants will receive a link to the online platform Meplis Care Monitor via mail in May/June 2020. Following the link, they will be able to register first and then to complete a questionnaire. Within the scope of this questionnaire participants will be asked about various aspects of their work environment (work relations, job satisfaction, perception of the quality of care and workload) and mental health. In the course of the study, this procedure will be repeated annually for a total of 4 measurement occasions. In addition to the surveys, occasional focus groups (i.e., moderated interactive group discussions) will take place in a selection of hospitals between July 2021 and September 2023. The focus groups aim to understand clinicians' personal views and thoughts on the process of the intervention and to identify barriers and facilitators to implementation of the intervention.

Why have I been invited and am I eligible?

60 hospitals across the five European countries being involved have agreed to participate in this study. Within each participating hospital, all adult inpatient units including ICU and ER have been selected. You are eligible to participate if you (1) have direct patient contact and (2) meet the minimum standards of Directive 2013/55/EU amending Directive 2005/35/EC on the recognition of professional qualifications. You are invited to participate in this study, because you belong to the clinical staff of one of these departments and meet the selection criteria.

What will my participation in the study involve?

In order to participate in the survey, we ask you to register on Meplis Care Monitor after receiving your activation link. Following the instructions given in the questionnaire, we ask you to answer a number of questions about your perception of your working environment: the relationship with the doctors, the workload and staffing of the ward, any intention (if any) to leave the profession, patient safety, etc. The questionnaire takes approx. 20 minutes to complete; it should be completed within two weeks of receiving this invitation. Once you have successfully created an account and participated in the first survey, you will receive an automatically generated mail by Meplis Care Monitor for three more times, inviting you to participate in the annual survey. If you are new to this hospital and did not participate in the last measurement occasion(s) please reach out to the person instructed by the hospital in order to invite you to the Meplis Care Monitor platform.

Do I have to take part?

It is up to you to decide whether or not to take part. If you do decide to take part you will be given this information sheet to keep. After signing up at Meplis Care Monitor, you will be asked to express your consent before completing the questionnaire. You will not be able to engage in the survey prior to providing an informed consent. If you decide to take part you are still free to withdraw at any time and without giving a reason. The choice that you make will have no bearing on your job or on any work-related evaluations or reports. You may change your mind later and stop participating even if you agreed earlier.

Are there any benefits for me in joining the study?

You have no direct benefit from participation and will not be compensated for your participation (there are no costs or compensation associated with participation). However, we hope that your participation in the study will help us to gain more insight on how you perceive hospital care and the demands that are placed on clinical staff. By doing so, you can help to improve the working environment and safety of patient care.

Are there any risks for me in joining the study?

There are no disadvantages associated with participation. Neither the research team nor the hospital will know whether you have participated in the study or not. A possible refusal to participate in the study will have no adverse effect on your employment at this hospital. Nor will your answers have any effect on your participation. However, you may feel uncomfortable reading the information and answering the questions.

Who has approved this study?

The research project was submitted to the central and/or local ethics committees within your country for approval. This authority has not raised any objections to the design of the study.

Will information about me be kept confidentially?

If you agree to participate in this study, a unique code will be stored along with your answers. Nobody but you can make the connection between your identity, this code, and your answers. Your data will be processed in accordance with Regulation (EU) 2016/679 of 27 April 2016 on the protection of individuals with regard to the processing of personal data and on the free movement of such data, which entered into force on 25 May 2018. If the results of the study are published, your anonymity is guaranteed. The final results of the analyses will be published on the Magnet4Europe website (www.magnet4europe.eu) and will be publicly accessible. Your identity will not be revealed in any of these cases. Individual answers will not be published and also research reports will not include information on clinical staff or individual hospitals. Neither the hospital nor your superiors will have access to individual answers.

Magnet4Europe - Improving Mental Health and Wellbeing in the Health Care Workplace

Consent to participate in the study

	Mark 'Yes' to continue
I hereby declare that I have been informed in an understandable manner about the nature, method and purpose of this study.	Yes
I agree to participate in this scientific research.	Yes
I am aware that participation in these studies does not entail any additional costs and that there is no financial benefit.	Yes
I am aware that I can withdraw at any time up to the moment that the data is kept in the database without having to make a statement and without this in any way affecting me.	Yes
I understand that my data is collected and registered confidentially, and that the principal investigator guarantees its confidentiality.	Yes

Date	Signature

Magnet4Europe - Improving Mental Health and Wellbeing in the Health Care Workplace

Dear Sir/Madam,

you have been invited to take part in a research study that aims to improve mental health and wellbeing among health professionals in Europe. The project is funded under the European Union's Horizon 2020 Research and Innovation programme from 2020 to 2023 (Grant Agreement 848031). The study will be conducted in compliance with your country's ethics guidelines for scientific studies on individuals. Your participation will neither involve costs nor reimbursements. You can withdraw from the study at any time, without giving a reason. Before you decide whether to take part it is important for you to understand why the research is being done and what it will involve. Please take your time to read the following information carefully and to decide whether you wish to take part or not. Ask us if there is anything that is not clear or if you would like to receive more information. For questions, please reach out to the Magnet4Europe project coordinator prof. dr. Walter Sermeus at walter.sermeus@kuleuven.be

What is the purpose of the study?

Mental health and wellbeing are among the highest priorities of the public health agenda in the European Union (EU). A large-scale European study of hospital work conditions and associated nurse and patient outcomes revealed high rates of job dissatisfaction and burnout, with burnout rates varying between 10% and 78%. Physician burnout rates ranging from 25% to 60% have been reported, varying across organizations and medical specialties. Magnet4Europe aims to redesign clinical environments in sixty hospitals in six European countries (Belgium, United Kingdom, Germany, Ireland, Norway, and Sweden) to promote the mental health and wellbeing of health professionals. Magnet4Europe will implement an evidence-based intervention based on the successful Magnet Recognition Program®, a voluntary hospital designation for nursing care excellence by the American Nurses Credentialing Center. Countless studies have shown that Magnet-recognized hospitals have lower health professional burnout and safer patient care suggesting that the Magnet journey is an intervention that results in positive changes Magnet4Europe seeks to achieve.

How does this research study work?

This research study will be carried out between January 2020 and December 2023 in adult inpatient units. The intervention itself will officially start in September 2020 in participating European hospitals and will target the five overarching principles of the Magnet® concept: structural empowerment of clinical staff, transformational leadership, exemplary and evidence-based professional practice, new knowledge, innovations, and improvements, and empirical outcomes. These principles will serve as a continuous feedback loop as to whether organizational changes are producing the intended outcomes for staff and patients. The Magnet® blueprint provides definitions of the principles and gives examples of evidence-based indicators that reflect progress toward achieving them. To facilitate the implementation process, each participating European hospital receives support by one to one twinning with an experienced Magnet recognized hospital including annual on-site visits and by the creation of a critical mass of participating hospitals drawing public interest and promoting innovation, and replication.

You are asked to participate in this study because your workplace belongs to one of the hospitals participating in the Magnet4Europe project. A selection of nurses and doctors working at the participating hospitals will be asked to participate in focus groups (i.e moderated interactive group discussions) or individual interviews. Focus groups and interviews will be used to investigate experiences and perceptions of the implementation process and to identify factors that hinder or facilitate the implementation of the intervention.

Why have I been invited and am I eligible?

60 hospitals across the six European countries being involved have agreed to participate in this study. Within each participating hospital, all adult inpatient units including ICU and ER have been selected. You are eligible to participate if you (1) have direct patient contact and (2) meet the minimum standards of Directive 2013/55/EU amending Directive 2005/35/EC on the recognition of professional qualifications. You are invited to participate in this study, because you belong to the clinical staff of one of these departments and meet the selection criteria.

What will my participation in the study involve?

We want to investigate perceptions of the clinical work environment and to monitor the mental health and well-being of healthcare professionals among the hospitals participating in Magnet4Europe. Participation means that you will participate in a focus group discussion (i.e together with about 2-7 other people from your hospital) or in an individual interview that will be about your experiences and experiences of the Magnet4Europe project at your workplace. The interviews and the focus group discussions are estimated to take about 30–60 minutes and 90–120 minutes respectively. The interviews and focus group discussions are conducted in person or via virtual means. The interviews and focus group discussions will be recorded through audio recording so that they can later be transcribed verbatim and analyzed.

Do I have to take part?

Your participation is voluntary and you can choose to cancel the participation at any time. If you choose not to participate or want to cancel your participation, you do not need to state why, nor will it affect your work or employment in the future. If you wish to cancel your participation, you should contact the person responsible for the study (see contact details at the end of the information letter).

Are there any benefits or risks for me to participate in the study?

You have no direct benefit from participation and will not be compensated for your participation (there are no costs or compensation associated with participation). However, we hope that your participation in the study will help us to gain more insight on how you perceive the implementation process in your hospital. By doing so, you can help to improve the working environment and safety of patient care. There are no disadvantages associated with participation. You should be aware, however, that the questions discussed during the interviews and focus groups can elicit discomfort or other emotional responses. If you feel that you need to talk to someone after the interviews, we recommend that you contact occupational health care expert at your hospital.

Will information about me be kept confidentially?

The research being done in the community may draw attention and if you participate you may be asked questions by other people in the community. We will not be sharing any information about you to anyone outside of the research team. If you participate in a focus group discussion, we will ask you and others in the group not to talk to people outside the group about what was said in the group. We will, in other words, ask each of you to keep what was said in the group confidential. You should know, however, that we cannot stop or prevent participants who were in the group from sharing things that should be confidential.

Who has approved this study?

The research project was submitted to the central and/or local ethics committees within your country for approval. This authority has not raised any objections to the design of the study.

How do I get information about the results of the study?

The results of the study will be compiled at the group level and reported in such a way that no individual individuals will be able to be identified. The results of the study will be published in scientific journals and research reports in such a way that individual answers, individuals, clinics or hospitals cannot be identified. Results from the study will also be published on Magnet4Europe's website, www.magnet4europe.eu, but will not contain information that allows individuals or hospitals to be identified.

Feel free to contact us if you have any questions or if you would like more information.

Consent to participate in the study

I have been given oral and written information about the study and have had the opportunity to ask questions. I may retain the written information.

☐ I agree to participate in an interview or focus group discussion within the study Magnet4Europe

☐ I agree that information about me is processed in the manner described in the research information.

Date	Signature

Sermeus W, *et al. BMJ Open* 2022; 12:e059159. doi: 10.1136/bmjopen-2021-059159
