# Supplementary material for: A workplace organisational intervention to improve hospital nurses’ and physicians’ mental health: study protocol for the Magnet4Europe wait list cluster randomised controlled trial
Source: BMJ Open. 2022 Jul 28;12(7):e059159. doi: 10.1136/bmjopen-2021-059159 (PMC9341186; doi:10.1136/bmjopen-2021-059159)
Supplement: Author's manuscript [file bmjopen-2021-059159.draft_revisions.pdf]

BMJ Open is committed to open peer review. As part of this commitment we make the peer review history of every article we publish publicly available.

When an article is published we post the peer reviewers' comments and the authors' responses online. We also post the versions of the paper that were used during peer review. These are the versions that the peer review comments apply to.

The versions of the paper that follow are the versions that were submitted during the peer review process. They are not the versions of record or the final published versions. They should not be cited or distributed as the published version of this manuscript.

BMJ Open is an open access journal and the full, final, typeset and author-corrected version of record of the manuscript is available on our site with no access controls, subscription charges or pay-per-view fees (http://bmjopen.bmj.com).

If you have any questions on BMJ Open's open peer review process please email info.bmjopen@bmj.com

BMJ Open

A workplace organizational intervention to improve hospital nurses' and physicians' mental health: study protocol for the Magnet4Europe wait-list cluster randomized controlled trial

Journal:	*BMJ Open*
Manuscript ID	bmjopen-2021-059159
Article Type:	Protocol
Date Submitted by the Author:	09-Nov-2021
Complete List of Authors:	Sermeus, Walter; KU Leuven, Public Health & Primary Care<br>Aiken, Linda; University of Pennsylvania, School of Nursing<br>Ball, Jane; University of Southampton, Faculty of Health Sciences<br>Bridges, Jackie; University of Southampton<br>Bruyneel, Luk; KU Leuven, Public Health & Primary Care<br>Busse, Reinhard; Technische Universität Berlin, Health Care Management<br>De Witte, Hans; KU Leuven, Occupational & Organisational Psychology and Professional Learning<br>Dello, Simon; KU Leuven, Public Health & Primary Care<br>Drennan, Jonathan; University College Cork National University of Ireland<br>Eriksson, Lars E; Karolinska Institutet, Department of Learning, Informatics, Management and Ethics<br>Griffiths, Peter; University of Southampton, Faculty of Health Sciences<br>Kohnen, Dorothea; KU Leuven Faculty of Psychology and Educational Sciences, Work, Organisational, and Personnel Psychology<br>Köppen, Julia; Technical University Berlin, Department of Healthcare Management<br>Lindqvist, Rikard; Karolinska Institutet<br>Maier, Claudia; Technische Universitat Berlin, Department of Healthcare Management<br>McHugh, Matthew; University of Pennsylvania, School of Nursing, Center for Health Outcomes and Policy Research<br>McKee, Martin; European Centre on Health of Societ,<br>Rafferty, Anne Marie; King's College London, Florence Nightingale School of Nursing and Midwifery<br>Schaufeli, Wilmar; KU Leuven<br>Sloane, Douglas; University of Pennsylvania<br>Alenius, Lisa Smeds; Karolinska Institutet<br>Smith, Herbert; University of Pennsylvania,<br>Magnet4Europe consortium, Magnet4Europe consortium; Magnet4Europe consortium
Keywords:	Health policy < HEALTH SERVICES ADMINISTRATION & MANAGEMENT, Protocols & guidelines < HEALTH SERVICES ADMINISTRATION & MANAGEMENT, MENTAL HEALTH, Human resource management < HEALTH SERVICES ADMINISTRATION & MANAGEMENT, Organisation of health services < HEALTH SERVICES ADMINISTRATION & MANAGEMENT

SCHOLARONE™
Manuscripts

A WORKPLACE ORGANIZATIONAL INTERVENTION TO IMPROVE HOSPITAL NURSES' AND PHYSICIANS' MENTAL HEALTH: STUDY PROTOCOL FOR THE MAGNET4EUROPE WAIT-LIST CLUSTER RANDOMIZED CONTROLLED TRIAL

Authors

Sermeus Walter, KU Leuven, Belgium (first author)

Aiken Linda, University of Pennsylvania, USA (first author)

Ball Jane, University of Southampton, UK

Bridges Jackie, University of Southampton, UK

Bruyneel Luk, KU Leuven, Belgium

Busse Reinhard, Technical University Berlin, Germany

De Witte Hans, KU Leuven, Belgium

Dello Simon, KU Leuven, Belgium

Drennan Jonathan, University College Cork, Ireland

Eriksson Lars E., Karolinska Institutet, Sweden

Griffiths Peter, University of Southampton, UK

Kohnen, Dorothea, KU Leuven, Belgium

Köppen Julia, Technical University Berlin, Germany

Lindqvist Rikard, Karolinska Institutet, Sweden

Maier Claudia, Technical University Berlin, Germany

McHugh Matthew, University of Pennsylvania, USA

McKee Martin, London School of Hygiene and Tropical Medicine, UK

Rafferty Anne Marie, King's College London, UK

Schaufeli Wilmar, KU Leuven, Belgium

Sloane Douglas, University of Pennsylvania, USA

Smeds Alenius Lisa, Karolinska Institutet, Sweden

Smith Herbert, University of Pennsylvania, USA

For the Magnet4Europe consortium

Corresponding author: Walter Sermeus, Leuven Institute for Healthcare Policy, KU Leuven, Kapucijnenvoer 35, 3000 Leuven, Belgium; e-mail: walter.sermeus@kuleuven.be

WORD COUNT

Words: 4779

For peer review only - http://bmjopen.bmj.com/site/about/guidelines.xhtml

ABSTRACT

Introduction The increasing burden of mental distress reported by healthcare professionals is a matter of serious concern and there is a growing recognition of the role of the workplace in creating this problem. Magnet® hospitals, a model shown to attract and retain staff in US research, creates positive work environments that aim to support the well-being of healthcare professionals.

Methods and analysis Magnet4Europe is a cluster randomized controlled trial, with wait list controls, designed to evaluate the effects of organizational redesign, based on the Magnet® model, on nurses' and physicians' well-being in general acute care hospitals, using a multicomponent implementation strategy. The study will be conducted in more than 60 general acute care hospitals in Belgium, England, Germany, Ireland, Norway, and Sweden. The primary outcome is burnout among nurses and physicians, assessed in longitudinal surveys of nurses and physicians at participating hospitals. Additional data will be collected from them on perceived work environments, patient safety, and patient quality of care and will be triangulated with data from medical records, including case mix adjusted in-hospital mortality. The process of implementation will be evaluated using qualitative data from focus group and key informant interviews.

Ethics and dissemination This study was approved by the Ethics Committee Research UZ/KU Leuven, Belgium; additionally, ethics approval is obtained in all other participating countries either through a central or decentral authority. Findings will be disseminated at conferences, through peer reviewed manuscripts, and via social media.

Trial registration number ISRCTN10196901.

For peer review only - http://bmjopen.bmj.com/site/about/guidelines.xhtml

STRENGTHS AND LIMITATIONS OF THIS STUDY

- Magnet4Europe uses a cluster randomized controlled trial design with wait-list controls in over 60 hospitals in six European countries.
- A multicomponent strategy is used to support implementation of a complex intervention at the hospital level, aimed to improve clinician well-being.
- The Magnet4Europe intervention is tailored to European hospitals, facilitating implementation and transferability without disrupting clinical practice.
- Contextual factors such as different health systems within and across countries may impact the standardized Magnet4Europe intervention and unintentionally lead to variation in the intervention and outcomes.
- Contamination of the wait-list control group cannot be ruled out.
- Embedding a complex intervention at hospital-level takes time and any effect might become apparent only after the study ends.

For peer review only

INTRODUCTION

Mental health and well-being are high priorities for the European Union (EU).[1] Mental health conditions account for 22% of the EU's disease burden measured in Years Lived with Disability, thus imposing a significant burden on individuals, society and the economy.[2] Health workers experience greater levels of job-related burnout and other mental health disorders than those in other sectors.[3,4] An earlier European study of working conditions in European hospitals, identified high rates of job dissatisfaction and burnout, with burnout rates among nurses varying from 10% to 78%[5,6] while the corresponding figures from studies of physicians ranged from 25% to 60%, varying among organisations and medical specialties.[7–10] Burnout in the healthcare workforce not only impacts on those experiencing it, leading to depression, substance abuse and even suicide,[11–15] but is also associated with worse patient outcomes, lower patient satisfaction,[6] medical errors,[16] reduced quality and safety[17] and reduced efficiency of hospitals.[18] The resulting lost productivity, combined with current recruitment challenges, further threatens the already overstretched health workforce, widening the gap between provision of health services and population needs.[19]

This situation has been exacerbated by the COVID-19 pandemic,[20] increasing anxiety and stress among clinicians[21] and further impacting on health workers' mental health.[22–24]

Effective, affordable and sustainable interventions to improve health professionals' mental health and well-being are essential if we are to interrupt the vicious cycle of high burnout, worsening mental health, lost productivity, and unsafe care.[25] Interventions to prevent burnout can be divided into two types: firstly, psychological interventions aimed at individual level and secondly workplace redesign. There is only limited evidence of the impact of individual psychological approaches to improve resilience and coping skills (e.g. mindfulness).[26] The second approach, redesigning the organizational environment in which health professionals provide care, aims to reduce modifiable sources of clinician stress and burnout, reinforce clinicians' perceptions of well-being, and enhance their autonomy and control over the conditions of their work. There is substantial research pointing to organizational redesign as the most promising of these two approaches.[27]

For peer review only - http://bmjopen.bmj.com/site/about/guidelines.xhtml

The finding that the causes underlying burnout in nurses and physicians are similar and, especially, are rooted within the work environment[28–31] provides a compelling rationale for prioritizing the redesign of hospital environments to address this issue. In the 1980s, hospitals in the US were faced with high nurse turnover,[32,33] low retention, and increasing early retirement rates.[34] Despite the general nursing shortage, some US hospitals - later referred to as Magnet® hospitals - were more successful in recruiting and retaining nurses by creating a positive work environment. Features characterizing these hospitals included flat decentralized organizational structures, empowering frontline staff in decision making and transformational leadership.[35] The original characteristics of these hospitals[36] were developed into fourteen forces of Magnetism and later configured into five components of the Magnet® model that was used to establish the criteria for Magnet® designation.[37] A robust body of evidence, primarily stemming from US hospitals, documents the association of the Magnet® model with improved well-being of staff including lower burnout, higher job satisfaction, lower intent to leave their job,[38,39] in addition to positive financial outcomes for organizations,[40,41] higher levels of patient satisfaction,[42,43] and improved clinical outcomes.[44,45] Longitudinal panel studies have found that hospitals that follow the Magnet® model improve their work environments, well-being of staff and patient outcomes, compared to other hospitals. Two international pilots tested the transferability of the Magnet® model, demonstrating the feasibility of achieving positive results outside the USA. One, in England, focused primarily on the implementation of the Magnet® process.[46] The other, a four-hospital intervention with accompanying evaluation in Russia and Armenia, used the Magnet® model with twinning with Magnet® recognized hospitals.[47] In both cases, the intervention hospitals improved their work environments substantially from their baselines within the two-year intervention and achieved better outcomes for staff, such as reduced dissatisfaction, intent to leave, and emotional exhaustion, while perceived quality of care improved. Despite the large body of evidence and recent uptake in two European hospitals, Magnet® organizational redesign initiatives have not been adopted widely in Europe.

For peer review only - http://bmjopen.bmj.com/site/about/guidelines.xhtml

The aim of Magnet4Europe is to evaluate the ability of organizational redesign in European hospitals to improve nurses' and physicians' mental health and well-being, guided by the Magnet® blueprint of organizational redesign and supported by a multicomponent implementation strategy.

METHODS AND ANALYSIS

Study design and setting

Magnet4Europe will use a usual-practice cluster randomized controlled trial with wait-list controls in a nested mixed-methods evaluation. The study protocol for this study follows "the Standard protocol items: recommendations for intervention trials 2013 statement" (SPIRIT; supplementary file 1).[48] The setting will be acute general hospitals in six European countries (Belgium, England, Germany, Ireland, Norway, and Sweden). These countries represent two dominant types of health systems, Bismarck and Beveridge,[49] that vary with regard to the extent of national government intervention in health services delivery, something that may be a factor in uptake of innovation.

Eligibility

European hospitals are eligible to participate if: (1) no Magnet® designation by the American Nursing Credentialing Centre (ANCC) had been acquired in the past or at the time of the start of the intervention, (2) bed size ≥150, and (3) the hospital is focused on acute care for adults, including wards in the area of internal medicine and/or surgery. Excluded are highly specialized hospitals, e.g., psychiatric hospitals, tropical medicine, or paediatrics.

Within each hospital, registered nurses, and physicians (including residents) will be eligible to participate in the quantitative and qualitative evaluation of the intervention if they: (1) have direct patient contact, (2) meet the minimum qualifications as stipulated by Directive 2013/55/EU amending Directive 2005/36/EC on the recognition of professional qualifications, and (3) work on adult inpatient units including intensive care units (ICU) and the emergency room (ER). Nurses and physicians working in specialized units such as neonatology, paediatrics, obstetrics, psychiatry, operating room, pathology, microbiology, radiology, and medical imaging will be excluded.

For peer review only - http://bmjopen.bmj.com/site/about/guidelines.xhtml

Study sample

A two-phased non-probability strategy will be used to sample organisations at the hospital level. First, in February and March 2019, principal investigators in the six European countries contacted eligible hospitals (see below) within those countries to consider taking part in the study using various recruitment strategies. Hospitals were informed of the Magnet4Europe objectives and methodology and were invited to show their interest in participating by submitting a letter of intent by March 2019. In spring 2020, participating hospitals (some of the sites and other new ones), were asked to confirm their commitment by signing an agreement committing to participate for the full duration of the study period and adhere to the Magnet4Europe protocol.

For peer review only

Sample size will be determined based on the power calculation for a cluster randomized controlled trial with before-and-after measures (CRT-BA).[50] The calculated design effect is based on average cluster size and the intraclass correlation coefficient (ICC), a measure of the amount of dependency among observations within classes (i.e. hospitals in our study).[50,51] Power calculation will be performed for the primary outcome of interest, burnout, measured by the emotional exhaustion subscale of the Maslach Burnout Inventory [52]. The estimated prevalence rate of emotional exhaustion is 30% based upon the team's earlier RN4CAST study.[6] In order to detect a 20% relative reduction (i.e. a 6 percentage point reduction) in burnout with a power of 80% and an alpha of 5%, an average cluster size of 200 and an ICC of 0.025,[53] this study will require 10,253 health professionals in 51 clusters per measurement occasion (at endpoint of the study).

Based on the power calculation, we aim to include 60 general acute care hospitals in six countries (Belgium, England, Germany, Ireland, Norway, and Sweden) and recruit at least 200 professionals per hospital.

Randomization

Randomization to intervention and control group is at the hospital level. Within each country hospitals will be matched on three characteristics, (1) bed size, (2) teaching status and (3) high technology, using a Euclidian distance matrix based on the smallest within pair covariate distance. Subsequently, simple

For peer review only - http://bmjopen.bmj.com/site/about/guidelines.xhtml

random sampling - allowing for selection with equal probability - will be used to allocate one hospital within each matched pair of hospitals to the immediate intervention group or the wait list control group. Both immediate intervention group and wait list controls will be informed which group they have been allocated to. Given the characteristics of the intervention in which active participation of the hospitals is required, blinding to the intervention will not be possible.

Intervention

A multicomponent intervention comprising organizational redesign of hospitals will be implemented in both arms of the study. The intervention was launched in the immediate intervention group in month 10 of the project (October 2020) and will have a total duration of 31 months. The intervention in the usual-practice wait-list control group will be initiated in month 17 (May 2021) and will last for 24 months (Figure 1).

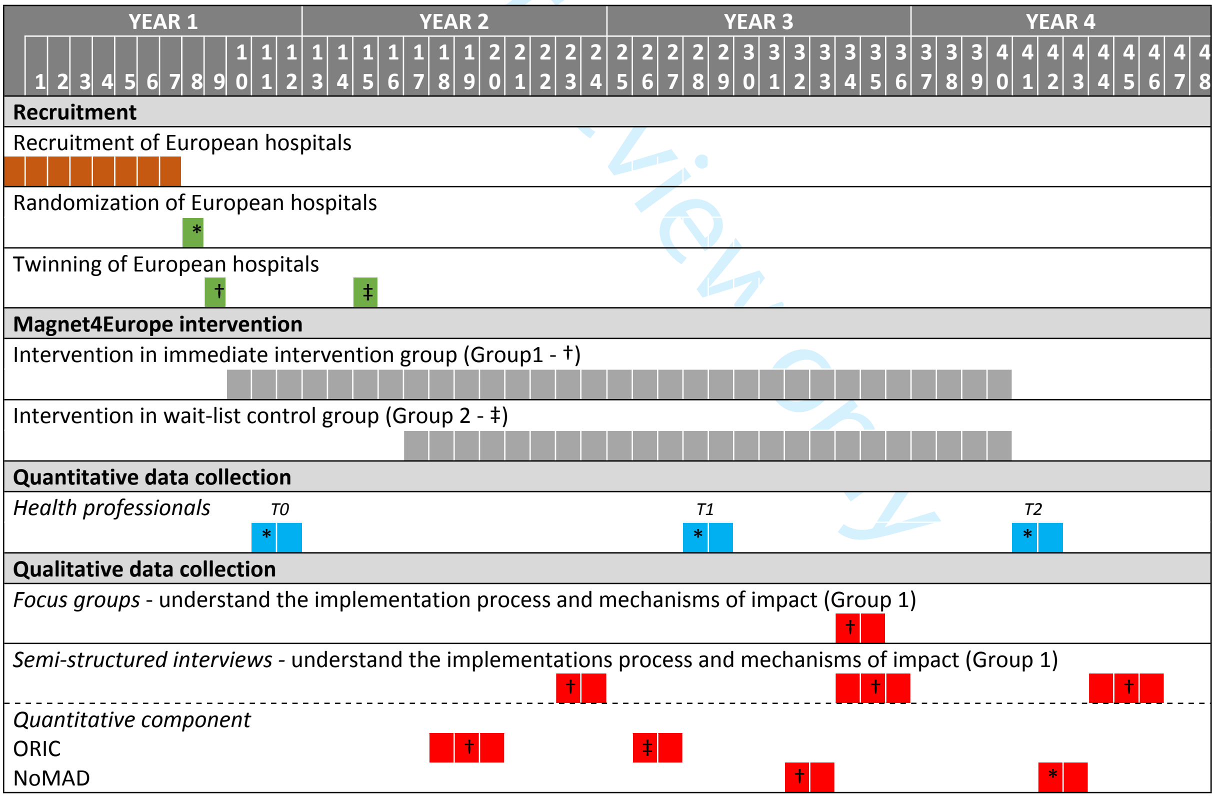

Figure 1 -Magnet4Europe timeline

*† - Activity for group 1; ‡ - Activity for group 2; * - Activity for both groups*

For peer review only

The intervention consists of five distinct components: The Magnet blueprint, one-to-one twinning between European hospitals and US Magnet designated hospitals, learning collaboratives involving group meetings to share best practices, critical mass, and communication and feedback.

The first part of the intervention comprises the elements outlined in the Magnet® manual of organizational redesign. This contains a step-by-step blueprint through the five overarching Magnet® components: (1) structural empowerment of clinical staff; (2) transformational leadership; (3) exemplary and evidence-based professional practice; (4) new knowledge, innovations, and improvements; and (5) empirical outcomes. These principles serve as continuous feedback loops as to whether organizational changes are producing the intended outcomes for health professionals and patients. The manual provides definitions of the principles and gives examples of evidence-based indicators that reflect progress toward achieving them. Using the Magnet4Europe Gap Analysis Tool (adapted from the ANCC Magnet® Gap Analysis tool), each participating European hospital will, in collaboration with their Magnet® twinning partner, perform a gap analysis that will illuminate the gap between the as-is situation and the aspirational organizational features as delineated in the Magnet® blueprint. The tool should be used for initial and continuous assessment of workplace redesign. This will increase the ability of hospitals to identify and prioritize learning and infrastructure needs, while also identifying initiative-wide gaps and exemplars for systematic improvement and recognition. Drawing on the results of the gap analysis, each hospital will be responsible for tailoring and individualizing the interventions to their hospital-specific context and developing a concrete, written action plan that will be monitored and executed during the course of the intervention period. Results of the gap analysis and the effect of tailored interventions will serve as feedback to enhance learning.

The second intervention component consists of one-to-one twinning between European hospitals aspiring to implement the Magnet® model and longstanding US Magnet® designated hospitals. This seeks to promote capacity building through transfer of knowledge, skills, tools, technologies and best practices, thereby supporting positive reciprocal organizational changes.[54] An equal number of U.S.

For peer review only - http://bmjopen.bmj.com/site/about/guidelines.xhtml

Magnet® hospitals – recognized for having an excellent work environment – will be recruited; allowing for a 1:1 twinning ratio of EU intervention hospitals with U.S. Magnet Hospitals. The twinning pairs will meet every two weeks virtually and two face-to-face mentoring sessions are planned each year when travel is safe.

Third, learning collaboratives will take place throughout the duration of the project and will be accessed and attended by all European hospitals in which the intervention is taking place at that point in time. The learning collaboratives will also involve policymakers so as to promote sustainability. Learning collaboratives build on the concept of communities of practice,[55] involving people coming together to engage in a process of learning in a shared domain of interest. These will be held monthly using remote means of communication with the possibility of in-person meetings annually when travel is safe. Effective elements of collaboratives, i.e. learning sessions, action periods and a collaborative extranet, will be implemented.[56–58]

Fourth is the creation of a critical mass of participating hospitals promoting innovation, attracting public interest, engaging stakeholders, and fostering replication and scaling of the intervention. Achieving a critical mass is essential for sustainability and scale-up of the intervention.[59] Critical mass shifts the context from one in which the change activities are unusual to one in which they are the norm. This long-standing principle in organizational sociology suggests that organizations become more open to and actively seek out change as the intervention gains legitimacy among peer institutions; this is particularly true in complex workplaces such as those found in health care.[60,61] Critical mass also maximizes the network exchanges and opportunities for feedback that arise out of other elements of our intervention such as the learning collaborative. This is supported by recent experience with the Magnet® model.[62,63]

A fifth component of the intervention is to provide regular survey feedback to hospitals on clinicians' reports on work conditions and well-being on the hospital level, separately for physicians and nurses.[64] Hospitals will be anonymized using a randomly generated letter code allowing each

For peer review only - http://bmjopen.bmj.com/site/about/guidelines.xhtml

hospital to identify themselves, but not any of the other hospitals. Data will only be reported in aggregated form and will not highlight a single individual's data.

Evaluation of the effect of the intervention

A mixed-methods evaluation will be conducted to examine the effect of the intervention, including quantitative and qualitative components.

The guiding framework for our quantitative evaluation is the Quality Health Outcomes Model (QHOM) – as adapted by Aiken and colleagues[65] – based on a conceptual framework that sets out mechanisms relating organizational attributes to clinician and patient outcomes. Their model is theoretically founded on Donabedian's early conception of associations between structures, processes and outcomes[66] including the dynamism between model components that was proposed in the Health Quality Outcomes Model.[67] The Job Demands-Resources (JD-R) model also informed the choice of survey items, seeking to understand the intervention's impact on the imbalance between demands on the clinician and the resources available to clinicians to deal with these demands.[68] Job demands refer to aspects of the job that require sustained physical or mental effort and are associated with certain physiological and psychological costs. Job resources describe aspects of the job that are related to achieving work goals, reducing job demands and the associated physiological and psychological costs, and stimulating personal growth and development.

The qualitative part of the study aims first, to evaluate the implementation process, adaptation, reach and quality as well as mechanisms of impact manifested in participants' responses, mediators, unexpected pathways and consequences; and second, to explore barriers and enablers of the implementation and the experiences of the change process from the perspectives of health professionals and persons involved in leading the implementation.[69]

Quantitative evaluation

Quantitative data collection

For peer review only

Quantitative data collection across countries is standardized using an online data collection platform. Quantitative survey data will be collected at three points in time, i.e., at baseline (T0) in months 11-12 of the project timeline, at T1 (months 28-29) and T2 (months 41-42) (Figure 1) during an 8-week period. An open cohort design will be employed in which all eligible nurses and physicians are invited to participate in each of these three measurement points (T0 – T2). Nurses and physicians will be recruited within each hospital at baseline and will be asked to participate in surveys with repeated measures taken over the three points in time allowing partially longitudinal follow-up of a cohort of individuals. Initial non-participants and new employees will be invited to join the study at later measurement points. Regular reminders will be sent to any clinicians (registered on the platform) through the online data collection platform who have not or only partially completed the survey. Individual informed consent for participation will be obtained by the researchers at the first contact (supplementary file 2). Each record of informed consent is retained on the online platform.

Quantitative measures

The main survey for nurses and physicians contains a core set of outcomes and main explanatory and process variables directly linked to the Magnet4Europe Magnet® Gap Analysis Tool. All study measures are based on validated instruments.

All survey items will be translated in Dutch, English, German, Norwegian and Swedish. When available, validated translations of instruments will be used. If no validated translation is available, a pre-specified translation procedure will be applied. First, a forward translation will be performed by a researcher involved in this research; the language in which the item is translated is the native language of the researcher. Secondly, other researchers within the country research group will thoroughly assess the forward translation in multiple phases until consensus is researched on each item.

Primary outcome

Burnout is the primary outcome measure and will be measured on individual level using the 9-item Emotional Exhaustion (EE) subscale of the Maslach Burnout Inventory (MBI).[70] In addition, the Burnout Assessment Tool (BAT), a 12-item novel self-report questionnaire will also be used to measure

For peer review only - http://bmjopen.bmj.com/site/about/guidelines.xhtml

For peer review only

burnout.[71–73] The combination of both instruments to assess burnout, allows for: (1) a direct comparison of levels of emotional exhaustion with previous studies in the medical field (using the EE-subscale of MBI); (2) an up-to-date assessment of the more comprehensive burnout syndrome, as well as a reliable estimation of the number of respondents with severe burnout symptoms and a comparison with the level of burnout of the national workforce.

Covariates

Secondary outcome measures include work engagement (UWES-3),[74] job satisfaction,[75,76] depression (PHQ-2),[77] anxiety (GAD-2),[78] general health (SF-8),[79] sleep quality (PSQ),[80] intent to leave the hospital,[75,76] absenteeism and presenteeism (HPQ),[14] workability, work-life conflict, team commitment, organizational commitment,[81] and whether clinicians would recommend the hospital.

Core exploratory variables include work environment (PES-NWI),[82] various measures of staffing and workload (NPQS),[83] care left undone,[84] operational failures, emotional demands (SIMPH),[85] red tape,[86] role conflicts (NPQS), job control, skill use, value congruence, performance feedback, opportunities for learning and development[87] and intrinsic motivation (WEIMS).[88]

Supplementary survey items are used to test more specific hypotheses.[68,89] These items contain measures on qualitative job insecurity [90], emotional dissonance,[91] task variety (QEEW),[92] and role clarity (NQPS),[83] basic need satisfaction,[93] and engaging leadership.[94]

Productivity measures consist of staff turnover rate, sickness absence rate, number of vacancies, and the use of agency staff - measured at organization level - complemented by previously mentioned secondary outcome measures measured at individual level, i.e., absenteeism and presenteeism.

Data analysis

Regression models to estimate the association between the intervention and outcomes will include fixed effects of time, intervention, and intervention by time. Hospitals represent higher order units of analysis, and clinicians the lower order unit of analysis. Countries will be treated as fixed effects.

For peer review only - http://bmjopen.bmj.com/site/about/guidelines.xhtml

Repeated measures on nurses and physicians will also be appropriately modelled. More complex multilevel structural models will be employed to examine indirect and moderated associations between the process measures which we would expect to respond to the intervention and our outcome measures. All analyses will be conducted according to intention-to-treat. Data on hospital characteristics (e.g., size, teaching status) and clinician (e.g., gender, age) will be collected and used to adjust for potential confounders in the regression analyses. Statistical significance will be assessed at the 5% level. Occasional missing data will be limited by the electronic survey implementation using a force response option for each item. Data analysis will be conducted using SAS software, version 9.4 (and subsequent releases) of the SAS system for windows (SAS Institute Inc., Cary, NC, USA).

Process evaluation

Qualitative data collection

Data will be collected on context, implementation, and mechanisms of impact. Based on the results at baseline measurement, a purposive sample of two hospitals per country will be selected from the immediate intervention group for case studies to explore different aspects of the implementation process. Hospitals with the lowest burnout scores among clinicians will be contrasted with hospitals with the highest to explore potential differences in motivation and ambition in the hospitals. In total, four qualitative data collection periods are planned throughout the project (Figure 1). Two focus group interviews (one with 6-8 nurses, one with 6-8 physicians) will be conducted in each of the two case hospitals (per country) at one point in time (M34-35). This will generate 20 focus groups in total across all countries. A purposive sampling approach is used to recruit participants. We will invite physicians and nurses who work in direct patient care in clinical areas targeted by the Magnet intervention and most closely involved in implementing change. By doing so, we will aim for diversity within each staff group in terms of clinical areas and length of clinical experience. If focus groups cannot be convened (for example, recruitment difficulties), then each focus group will be replaced with 2 to 3 one-to-one interviews with similar staff types to the target focus group sample. Depending on practicalities, this

For peer review only - http://bmjopen.bmj.com/site/about/guidelines.xhtml

may result in a dataset that is generated from focus groups and/or one-to-one interviews. A minimum of 40 and maximum of 160 individuals will be involved in this part of the study.

In addition to the focus groups, two semi-structured individual interviews will be conducted at three time-points (months 23, 34 and 44) in each of the two case hospitals (per country). Persons directly involved in leading the implementation, i.e., the Magnet4Europe intervention coordinator, as well as another member of the interdisciplinary Magnet council will be invited to participate. This will lead to 60 interviews in total (across countries) over the course of the project. Where possible, we aim to interview the same two people across all time-points. If individuals leave the study, we will aim to recruit a replacement with a similar role and professional background. We will also aim for maximum diversity in professional backgrounds, e.g., one from nursing, one from medicine. A minimum of 30 and a maximum of 60 individuals will be involved in this part of the study.

To complement the qualitative evaluation, the 12-item Organizational Readiness for Implementing Change scale (ORIC)[95] and the 23-item Normalization Measure Development Questionnaire (NoMAD)[96,97] will be used to explore the readiness for change and the extent to which Magnet is integrated and sustained in the hospital over time. This will be done after the start of the intervention and at various time points. Magnet4Europe intervention coordinators and all other members of the interdisciplinary Magnet4Europe council in all hospitals will be invited to respond to the surveys. A minimum of 54 and a maximum of 324 individuals across all countries will be involved in this part of the study at each time point. If individuals leave and are replaced, their replacements will be invited to complete the survey on the next occasion.

Qualitative data analysis

Analysis of the qualitative data generated through focus groups and individual interviews will be guided by the Framework Analysis approach.[98] Data collection and analysis will be conducted by researchers in each of the Magnet4Europe countries, using a common coding template which will be developed based on initial themes. International meetings will be held regularly to discuss analysis and

For peer review only - http://bmjopen.bmj.com/site/about/guidelines.xhtml

findings. NVivo 12 (and subsequent releases) will be used for management and analysis of qualitative data.[99]

Data generated through collection of policy documents and general hospital information about context will be analysed through content analysis and/or discourse analysis[100] to explore different aspects of the organizational context in relation to the implementation process.

In the process evaluation we will analyse how organisational context links to the impact and sustainability of the Magnet programme. Emerging themes from the qualitative analysis will be used, together with the results from descriptive analyses of our quantitative measurements of readiness for change and of implementation normalization (NoMAD instrument), to explain variations in implementation between and within individual settings over time (Table 1).

Table 1 - Magnet4Europe Implementation Evaluation

**Context**
▪ General information about the hospital (e.g., type of hospital, size, organizational models, professional structure)<br>▪ Hospital policy documents (e.g., about staffing, work environment, leadership development, organizational plans)<br>▪ General country information (e.g., relevant legislation, financing of the healthcare sector)
**Implementation**
▪ Results from the Magnet4Europe Magnet® Gap Analysis Tool and the work plan of each hospital<br>▪ Implementation process survey data<br>▪ Data from interviews and focus groups
**Mechanisms of impact**
▪ Hospital policy documents (e.g., staffing, work environment, leadership development, organizational plans)<br>▪ Implementation process survey data<br>▪ Data from interviews and focus groups

For peer review only - http://bmjopen.bmj.com/site/about/guidelines.xhtml

Patient and public involvement

Patients and/or public were not involved in the design or development of research questions of this study.

ETHICS AND DISSEMINATION

Ethics

This study was approved by the Ethics Committee Research UZ/KU Leuven, Belgium; additionally, ethics approval is obtained in all other participating countries either through a central or decentral authority, to conduct nurse surveys as well as collect qualitative data. Any protocol amendments will be promptly reported to all relevant parties. An Ethical Advisory Committee was installed at the start of project and includes an independent ethics advisor with documented expertise to monitor ethics issues. The study is registered in the ISRCTN registry (ISRCTN10196901).

Dissemination

Magnet4Europe has a strong focus on disseminating evidence on the impact of work environment on mental health of health professionals. Findings will be published in peer reviewed journals and through other channels designed to reach a diverse community of researchers, practitioners, and other stakeholders. Gold open access will be used for the key findings of the study to ensure maximum exposure to a practice community. Beyond the funded life of the project the aim is to maximize accessibility by fully exploiting the opportunity for green open access and, where possible, identifying additional funds to support further gold open access publishing. Data management is compliant in line with the European Commission's Horizon 2020 Data Management Plan guidelines and FAIR-principles.

For peer review only - http://bmjopen.bmj.com/site/about/guidelines.xhtml

For peer review only

DISCUSSION

This paper describes the theoretical foundations, conceptual model, study design and methodological approaches of the EU H2020 Magnet4Europe project, which aims to evaluate the transfer, implementation, scale up and cost-effectiveness of the - Magnet® model of organizational redesign in a European context as a system-level approach to improve clinician well-being. Mental health and well-being is among one of the highest priorities of the public health agenda in the European Union.[1] This is based on the growing awareness and recognition of the magnitude of mental health problems and the burden on individuals, society, and economy. With the extraordinary conditions created by the coronavirus pandemic (COVID-19), this issue has taken an even more central role in the policy landscape.[20] COVID-19 is expected to have psychological impacts for health professional, in particular frontline workers that will be sustained over time.[101–104]

The research activities conducted within the project are expected to improve significantly the work environment of health professionals in hospitals, especially after the COVID-19 pandemic. In particular, Magnet4Europe proposes an organizational intervention, targeting the root of many of the problems (i.e. organizational and work-related stressors) facing health systems rather than focusing narrowly of their symptoms. It does so by means of a multi-centre study involving more than 60 hospitals across six European countries. By choosing a cluster randomized controlled design, this study is the first with the power to identify a causal association between implementing healthy workplace characteristics and improved staff wellbeing in European hospitals on a large scale. Two international pilot initiatives testing the transferability of Magnet key principles and accompanied by rigorous evaluations have informed the design of Magnet4Europe.[46,47] In both, the intervention hospitals improved their work environments substantially from their baselines at the end of the two-year intervention, and outcomes for staff improved. The Magnet4Europe intervention builds upon lessons learned from these pilot initiatives. The degree of innovation can also be seen in the use of a multi-faceted implementation strategy involving one-to-one twinning with international Magnet®-designated hospitals, creation of a critical mass, co-creation of solutions, development of learning collaboratives, benchmarking, and

For peer review only - http://bmjopen.bmj.com/site/about/guidelines.xhtml

interdisciplinary and management buy-in. In addition, Magnet4Europe makes a strong scientific contribution as it goes beyond traditional studies which focus solely on individual professions and disciplines separately. This allows us to study the interaction between physicians and nurses working in teams and the impact of healthy environments on their well-being and patient outcomes.

Magnet4Europe will also allow countries to learn from the experience of other health systems and understand the factors influencing their sustainability, as the study will be undertaken in six European countries representing the two dominant health system forms. The so-called Beveridge countries (England, Ireland, Sweden, Norway) are tax funded resulting in potentially more government health policy decision-making while the so-called Bismarck countries (Belgium, Germany) have an employer-based social insurance financing system, which potentially allows for more organizational variation. This study allows us to compare and contrast the impact of health care systems on the implementation and sustainability of the intervention.

The project involves dissemination and stakeholder engagement. Various stakeholders will be involved in the project including designees from international and European organizations who represent nursing professionals, health care employers, patients, and governments. Their main role is to raise awareness of the project and to support dissemination of the results as well as the Magnet4Europe consortium in formulating policy recommendations based on the scientific results.

For peer review only - http://bmjopen.bmj.com/site/about/guidelines.xhtml

AUTHORS' CONTRIBUTIONS

WS, LA, MMH and LB devised the study. WS, LA, MMH and LB drafted the protocol, with assistance from JB, JB, RB, HDW, SD, JD, LEE, PG, DK, JK, RL, CM, MM, AMR, WS, DS, LSA, HS. All authors provided edits and reviewed the manuscript for important intellectual content. All authors gave final approval of the version to be published and agreed to be accountable for all aspects of this work.

FUNDING

This study is funded by the European Union's Horizon 2020 research and innovation programme under the project Magnet4Europe: Improving Mental Health and Wellbeing in the Health Care Workplace (Grant Agreement No. 848031), starting 1st January 2020 until 31st December 2023. The materials presented here are the responsibility of the authors only. The EU Commission takes no responsibility for any use made of the information set out.

COMPETING INTERESTS

None declared.

ACKNOWLEDGMENTS

We thank the American Nurses Credentialing Center (ANCC) for granting the right to use Magnet® model as core element of the Magnet4Europe intervention. The Magnet® model is a trademark of ANCC registered in the United States of America and other jurisdictions, and is being used under license from ANCC. All rights are reserved by ANCC. ANCC's consent to the use of the Intervention Hospital Magnet® mark shall not be construed as ANCC sponsoring, participating, or endorsing the Magnet4Europe intervention. ©American Nurses Credentialing Center. Reproduced under license from the American Nurses Credentialing Center. All rights reserved.

For peer review only - http://bmjopen.bmj.com/site/about/guidelines.xhtml

MAGNET4EUROPE CONSORTIUM

The Magnet4Europe Consortium consists of Walter Sermeus (director), Luk Bruyneel, Hans De Witte, Wilmar Schaufeli, Simon Dello, Dorothea Kohnen (Belgium, Catholic University Leuven); Linda Aiken (codirector), Matthew McHugh, Mary Del Guidice, Herbert Smith, Timothy Cheney, Douglas Sloane (USA, University of Pennsylvania); Reinhard Busse, Claudia Maier, Julia Köppen, Joan Kleine (Germany, Technical University Berlin); Jonathan Drennan, Vera McCarthy, Elaine Lehane, Noeleen Brady, Anne Scott (Ireland, University College Cork); Ingeborg Strømseng Sjetne (Norway, Norwegian Institute of Public Health); Anners Lerdal, Monica Bukkøy Kjetland (Norway, Lovisenberg Diaconal Hospital); Lars E. Eriksson, Rikard Lindqvist, Lisa Smeds Alenius (Sweden, Karolinska Institutet); Jane Ball, Peter Griffiths, Jackie Bridges, Sydney Anstee (England, University of Southampton); Anne Marie Rafferty (England, King's College London); Martin McKee (England, London School of Hygiene and Tropical Medicine); Paul Van Aken, Danny Van Heusden, Kaat Siebens, Peter Van Bogaert (Belgium, University Hospital Antwerp).

For peer review only - http://bmjopen.bmj.com/site/about/guidelines.xhtml

REFERENCES

1 European Commission. Green Paper: Improving the Mental Health of the Population: Towards a Strategy on Mental Health in the European Union. OOPEC 2005.

2 Leka S, Jain A. Eu Compass for Action on Mental Health and Well-Being: Mental Health in the Workplace in Europe - consensus paper. 2014.

3 Kim M-S, Kim T, Lee D, *et al.* Mental disorders among workers in the healthcare industry: 2014 national health insurance data. *Ann Occup Environ Med* 2018;**30**:31. doi:10.1186/s40557-018-0244-x

4 Shanafelt TD, Boone S, Tan L, *et al.* Burnout and Satisfaction With Work-Life Balance Among US Physicians Relative to the General US Population. *Arch Intern Med* 2012;**172**:1377. doi:10.1001/archinternmed.2012.3199

5 Aiken LH, Sloane DM, Bruyneel L, *et al.* Nurse staffing and education and hospital mortality in nine European countries: a retrospective observational study. *Lancet* 2014;**383**:1824–30. doi:10.1016/s0140-6736(13)62631-8

6 Aiken LH, Sermeus W, Van den Heede K, *et al.* Patient safety, satisfaction, and quality of hospital care: cross sectional surveys of nurses and patients in 12 countries in Europe and the United States. *BMJ* 2012;**344**:e1717. doi:10.1136/bmj.e1717

7 Dyrbye LN, Burke SE, Hardeman RR, *et al.* Association of Clinical Specialty With Symptoms of Burnout and Career Choice Regret Among US Resident Physicians. *JAMA* 2018;**320**:1114. doi:10.1001/jama.2018.12615

8 Rotenstein LS, Torre M, Ramos MA, *et al.* Prevalence of burnout among physicians a systematic review. *JAMA - J Am Med Assoc* 2018;**320**:1131–50. doi:10.1001/jama.2018.12777

9 Van Gerven E, Vander Elst T, Vandenbroeck S, *et al.* Increased risk of burnout for physicians

For peer review only

For peer review only - http://bmjopen.bmj.com/site/about/guidelines.xhtml

and nurses involved in a patient safety incident. *Med Care* 2016;**54**:937–43. doi:10.1097/MLR.0000000000000582

10 Vandenbroeck S, Van Gerven E, De Witte H, *et al.* Burnout in Belgian physicians and nurses. *Occup Med (Chic Ill)* 2017;**67**:546–54. doi:10.1093/occmed/kqx126

11 Schernhammer E. Taking Their Own Lives — The High Rate of Physician Suicide. *N Engl J Med* 2005;**352**:2473–6. doi:10.1056/NEJMp058014

12 Dyrbye LN, Thomas MR, Massie FS, *et al.* Burnout and suicidal ideation among U.S. medical students. *Ann Intern Med* 2008;**149**:334–41. doi:10.7326/0003-4819-149-5-200809020-00008

13 Schwenk TL, Davis L, Wimsatt LA. Depression, stigma, and suicidal ideation in medical students. *JAMA* 2010;**304**:1181–90. doi:10.1001/jama.2010.1300

14 Kessler RC, Barber C, Beck A, *et al.* The World Health Organization Health and Work Performance Questionnaire (HPQ). *J Occup Env Med* 2003;**45**:156–74. doi:10.1097/01.jom.0000052967.43131.51

15 Davis MA, Cher BAY, Friese CR, *et al.* Association of US Nurse and Physician Occupation With Risk of Suicide. *JAMA Psychiatry* 2021;**78**:651–8. doi:10.1001/JAMAPSYCHIATRY.2021.0154

16 Shanafelt TD, Balch CM, Bechamps G, *et al.* Burnout and medical errors among American surgeons. *Ann Surg* 2010;**251**:995–1000. doi:10.1097/SLA.0b013e3181bfdab3

17 Salyers MP, Bonfils KA, Luther L, *et al.* The Relationship Between Professional Burnout and Quality and Safety in Healthcare: A Meta-Analysis. *J Gen Intern Med* 2017;**32**:475–82. doi:10.1007/s11606-016-3886-9

18 Dewa CS, Loong D, Bonato S, *et al.* The relationship between physician burnout and quality of healthcare in terms of safety and acceptability: a systematic review. *BMJ Open* 2017;**7**:e015141. doi:10.1136/bmjopen-2016-015141

For peer review only - http://bmjopen.bmj.com/site/about/guidelines.xhtml

19 Dewa CS, Jacobs P, Thanh NX, *et al.* An estimate of the cost of burnout on early retirement and reduction in clinical hours of practicing physicians in Canada. *BMC Health Serv Res* 2014;**14**:254. doi:10.1186/1472-6963-14-254

20 Muller AE, Hafstad EV, Himmels JPW, *et al.* The mental health impact of the covid-19 pandemic on healthcare workers, and interventions to help them: A rapid systematic review. Psychiatry Res. 2020;**293**:113441. doi:10.1016/j.psychres.2020.113441

21 Vanhaecht K, Seys D, Bruyneel L, *et al.* COVID-19 is having a destructive impact on health-care workers' mental well-being. *Int J Qual Heal Care* 2021;**33**. doi:10.1093/intqhc/mzaa158

22 Tan BYQ, Chew NWS, Lee GKH, *et al.* Psychological Impact of the COVID-19 Pandemic on Health Care Workers in Singapore. *Ann Intern Med* Published Online First: 2020. doi:10.7326/m20-1083

23 Pfefferbaum B, North CS. Mental Health and the Covid-19 Pandemic. *N Engl J Med* Published Online First: 2020. doi:10.1056/nejmp2008017

24 Walton M, Murray E, Christian MD. Mental health care for medical staff and affiliated healthcare workers during the COVID-19 pandemic. *Eur Hear J Acute Cardiovasc Care* 2020;**9**:241–7. doi:10.1177/2048872620922795

25 EU Joint Action on Mental Health and Wellbeing. European Framework for Action on Mental Health and Wellbeing. 2016.

26 Panagioti M, Panagopoulou E, Bower P, *et al.* Controlled interventions to reduce burnout in physicians a systematic review and meta-analysis. JAMA Intern. Med. 2017;**177**:195–205. doi:10.1001/jamainternmed.2016.7674

27 West CP, Dyrbye LN, Erwin PJ, *et al.* Interventions to prevent and reduce physician burnout: a systematic review and meta-analysis. *Lancet* 2016;**388**:2272–81. doi:10.1016/S0140-6736(16)31279-X

For peer review only - http://bmjopen.bmj.com/site/about/guidelines.xhtml

For peer review only

28 West CP, Dyrbye LN, Shanafelt TD. Physician burnout: contributors, consequences and solutions. *J Intern Med* 2018;**283**:516–29. doi:10.1111/joim.12752

29 Kutney-Lee A, Wu ES, Sloane DM, *et al.* Changes in hospital nurse work environments and nurse job outcomes: An analysis of panel data. *Int J Nurs Stud* 2013;**50**:195–201. doi:10.1016/j.ijnurstu.2012.07.014

30 Shanafelt TD, Gorringe G, Menaker R, *et al.* Impact of organizational leadership on physician burnout and satisfaction. *Mayo Clin Proc* 2015;**90**:432–40. doi:10.1016/j.mayocp.2015.01.012

31 Diez-Pinol M, Dolan SL, Sierra V, *et al.* Personal and organizational determinants of well-being at work: the case of Swedish physicians. *Int J Health Care Qual Assur* 2008;**21**:598–610. doi:10.1108/09526860810900754

32 Hayes LJ, O'Brien-Pallas L, Duffield C, *et al.* Nurse turnover: a literature review - an update. *Int J Nurs Stud* 2012;**49**:887–905. doi:10.1016/j.ijnurstu.2011.10.001

33 Hayes LJ, O'Brien-Pallas L, Duffield C, *et al.* Nurse turnover: A literature review. *Int J Nurs Stud* 2006;**43**:237–63. doi:10.1016/j.ijnurstu.2005.02.007

34 Buerhaus PI. Current and future state of the US nursing workforce. *JAMA* 2008;**300**:2422–4. doi:10.1001/jama.2008.729

35 Upenieks V V. The interrelationship of organizational characteristics of magnet hospitals, nursing leadership, and nursing job satisfaction. *Health Care Manag (Frederick)* 2003;**22**:83–98. doi:10.1097/00126450-200304000-00002

36 Magnet hospitals. Attraction and retention of professional nurses. Task Force on Nursing Practice in Hospitals. American Academy of Nursing. *ANA Publ* 1983;:i–xiv, 1–135.

37 Blegen MA, Goode CJ, Spetz J, *et al.* Nurse staffing effects on patient outcomes: safety-net and non-safety-net hospitals. *Med Care* 2011;**49**:406–14.

For peer review only - http://bmjopen.bmj.com/site/about/guidelines.xhtml

doi:10.1097/MLR.0b013e318202e129

38 Kelly LA, McHugh MD, Aiken LH. Nurse outcomes in magnet® and non-magnet hospitals. *J Nurs Adm* 2012;**42**:S44-9. doi:10.1097/01.NNA.0000420394.18284.4f

39 Kutney-Lee A, Stimpfel AW, Sloane DM, *et al.* Changes in Patient and Nurse Outcomes Associated With Magnet Hospital Recognition. *Med Care* 2015;**53**:550–7. doi:10.1097/mlr.0000000000000355

40 Drenkard K. The business case for Magnet. *J Nurs Adm* 2010;**40**:263–71. doi:10.1097/NNA.0b013e3181df0fd6

41 Jayawardhana J, Welton JM, Lindrooth RC. Is there a business case for magnet hospitals? Estimates of the cost and revenue implications of becoming a magnet. *Med Care* 2014;**52**:400–6. doi:10.1097/MLR.0000000000000092

42 Stimpfel AW, Sloane DM, McHugh MD, *et al.* Hospitals Known for Nursing Excellence Associated with Better Hospital Experience for Patients. *Health Serv Res* 2016;**51**:1120–34. doi:10.1111/1475-6773.12357

43 Aiken LHH, Sloane DMM, Ball J, *et al.* Patient satisfaction with hospital care and nurses in England: an observational study. *BMJ Open* 2018;**8**:e019189. doi:10.1136/bmjopen-2017-019189

44 McHugh MD, Kelly LA, Smith HL, *et al.* Lower mortality in magnet hospitals. *Med Care* 2013;**51**:382–8. doi:10.1097/mlr.0b013e3182726cc5

45 Barnes H, Rearden J, McHugh MD. Magnet® Hospital Recognition Linked to Lower Central Line-Associated Bloodstream Infection Rates. *Res Nurs Health* 2016;**39**:96–104. doi:10.1002/NUR.21709

46 Aiken LH, Buchan J, Ball J, *et al.* Transformative impact of Magnet designation: England case

For peer review only

For peer review only - http://bmjopen.bmj.com/site/about/guidelines.xhtml

study. *J Clin Nurs* 2008;**17**:3330–7. doi:10.1111/j.1365-2702.2008.02640.x

47 Aiken LH, Poghosyan L. Evaluation of “Magnet Journey to Nursing Excellence Program” in Russia and Armenia. *J Nurs Scholarsh* 2009;**41**:166–74. doi:10.1111/j.1547-5069.2009.01268.x

48 Chan A-W, Tetzlaff JM, Altman DG, *et al.* SPIRIT 2013 statement: defining standard protocol items for clinical trials. *Ann Intern Med* 2013;**158**:200–7.

49 Saltman RB, Dubois HF. Social health insurance systems in western Europe. *Open Univ Press* Published Online First: 2004. doi:44,2

50 Hemming K, Taljaard M. Sample size calculations for stepped wedge and cluster randomised trials: a unified approach. *J Clin Epidemiol* 2016;**69**:137–46. doi:10.1016/j.jclinepi.2015.08.015

51 Woertman W, de Hoop E, Moerbeek M, *et al.* Stepped wedge designs could reduce the required sample size in cluster randomized trials. *J Clin Epidemiol* 2013;**66**:752–8. doi:10.1016/j.jclinepi.2013.01.009

52 Maslach C, Jackson SE. The measurement of experienced burnout. *J Organ Behav* 1981;**2**:99–113. doi:10.1002/job.4030020205

53 Li B, Bruyneel L, Sermeus W, *et al.* Group-level impact of work environment dimensions on burnout experiences among nurses: a multivariate multilevel probit model. *Int J Nurs Stud* 2013;**50**:281–91. doi:10.1016/j.ijnurstu.2012.07.001

54 Cadée F, Nieuwenhuijze MJ, Lagro-Janssen ALM, *et al.* The state of the art of twinning, a concept analysis of twinning in healthcare. *Global Health* 2016;**12**:66. doi:10.1186/s12992-016-0205-5

55 Wenger E, McDermomtt R, Snyder WM. *Cultivating Communities of Practice: A Guide to Managing Knowledge - Seven Principles for Cultivating Communities of Practice*. Harvard Business School Press 2002.

For peer review only

56 Mills PD, Weeks WB. Characteristics of successful quality improvement teams: lessons from five collaborative projects in the VHA. *Jt Comm J Qual Saf* 2004;**30**:152–62. doi:10.1016/s1549-3741(04)30017-1

57 Nembhard IM. Learning and improving in quality improvement collaboratives: which collaborative features do participants value most? *Health Serv Res* 2009;**44**:359–78. doi:10.1111/j.1475-6773.2008.00923.x

58 Yuan CT, Nembhard IM, Stern AF, *et al.* Blueprint for the dissemination of evidence-based practices in health care. *Issue Brief (Commonw Fund)* 2010;**86**:1–16.

59 Greenhalgh T, Robert G, Macfarlane F, *et al.* Diffusion of innovations in service organizations: systematic review and recommendations. *Milbank Q* 2004;**82**:581–629.

60 Meyer AD, Goes JB. Organizational Assimilation of Innovations: A Multilevel Contextual Analysis. *Acad Manag J* 1988;**31**:897–923. doi:10.5465/256344

61 Kimberly JR, Evanisko MJ. Organizational Innovation: The Influence of Individual, Organizational, and Contextual Factors on Hospital Adoption of Technological and Administrative Innovations. *Acad Manag J* 1981;**24**:689–713. doi:10.2307/256170

62 Richards MR, Lasater K, McHugh M. A Race to the Top? Competitive Pressure and Magnet Adoption Among US Hospitals 1997-2012. *Med Care* 2017;**55**:384–90. doi:10.1097/MLR.0000000000000650

63 Lasater KB, Richards MR, Dandapani NB, *et al.* Magnet hospital recognition in hospital systems over time. *Health Care Manage Rev* 2019;**44**:19–29. doi:10.1097/HMR.0000000000000167

64 Björklund C, Grahn A, Jensen I, *et al.* Does survey feedback enhance the psychosocial work environment and decrease sick leave? *http://dx.doi.org/101080/13594320601112169* 2007;**16**:76–93. doi:10.1080/13594320601112169

For peer review only

65 Aiken LH, Sochalski J, Lake ET. Studying outcomes of organizational change in health services. *Med Care* 1997;**35**:NS6-18.

66 Donabedian A. Evaluating the quality of medical care. *Milbank Q* 1966;**44**:166–203. doi:10.1111/j.1468-0009.2005.00397.x

67 Mitchell PH, Ferketich S, Jennings BM. Quality Health Outcomes Model. *J Nurs Scholarsh* 1998;**30**:43–6. doi:10.1111/j.1547-5069.1998.tb01234.x

68 Demerouti E, Nachreiner F, Bakker AB, *et al.* The job demands-resources model of burnout. *J Appl Psychol* 2001;**86**:499–512. doi:10.1037/0021-9010.86.3.499

69 Moore GF, Audrey S, Barker M, *et al.* Process evaluation of complex interventions: Medical Research Council guidance. *Bmj* 2015;**350**:h1258. doi:10.1136/bmj.h1258

70 Maslach C, Jackson SE. The measurement of experienced burnout. *J Organ Behav* 1981;**2**:99–113. doi:10.1002/job.4030020205

71 de Beer LT, Schaufeli WB, De Witte H, *et al.* Measurement invariance of the burnout assessment tool (Bat) across seven cross-national representative samples. *Int J Environ Res Public Health* 2020;**17**:1–14. doi:10.3390/ijerph17155604

72 Schaufeli WB, Desart S, De Witte H. Burnout assessment tool (Bat)—development, validity, and reliability. *Int J Environ Res Public Health* 2020;**17**:1–21. doi:10.3390/ijerph17249495

73 Hadžibajramović E, Schaufeli W, De Witte H. A Rasch analysis of the Burnout Assessment Tool (BAT). *PLoS One* 2020;**15**. doi:10.1371/journal.pone.0242241

74 Schaufeli WB, Shimazu A, Hakanen J, *et al.* An Ultra-Short Measure for Work Engagement: The UWES-3 Validation Across Five Countries. *Eur J Psychol Assess* Published Online First: October 2017. doi:10.1027/1015-5759/A000430

75 Wanous JP, Reichers AE, Hudy MJ. Overall job satisfaction: How good are single-item

For peer review only - http://bmjopen.bmj.com/site/about/guidelines.xhtml

measures? *J Appl Psychol* 1997;**82**:247–52. doi:10.1037/0021-9010.82.2.247

76 Wanous JP, Hudy MJ. Single-Item Reliability: A Replication and Extension. *Organ Res Methods* 2001;**4**:361–75. doi:10.1177/109442810144003

77 Kroenke K, Spitzer RL, Williams JBW. The patient health questionnaire-2: Validity of a two-item depression screener. *Med Care* 2003;**41**:1284–92. doi:10.1097/01.MLR.0000093487.78664.3C

78 Kroenke K, Spitzer RL, Williams JBW, *et al.* Anxiety disorders in primary care: Prevalence, impairment, comorbidity, and detection. *Ann Intern Med* 2007;**146**:317–25. doi:10.7326/0003-4819-146-5-200703060-00004

79 Ware J, Kosinski M, Dewey J, *et al.* How to score and interpret single-item health status measures: a manual for users of the SF-8™ Health Survey. 2008.

80 Buysse DJ, Reynolds CF, Monk TH, *et al.* The Pittsburgh sleep quality index: A new instrument for psychiatric practice and research. *Psychiatry Res* 1989;**28**:193–213. doi:10.1016/0165-1781(89)90047-4

81 Rahmadani VG, Schaufeli WB, Stouten J, *et al.* Engaging Leadership and Its Implication for Work Engagement and Job Outcomes at the Individual and Team Level: A Multi-Level Longitudinal Study. *Int J Environ Res Public Health* 2020;**17**:776. doi:10.3390/ijerph17030776

82 Lake ET. Development of the practice environment scale of the Nursing Work Index. *Res Nurs Health* 2002;**25**:176–88. doi:10.1002/nur.10032

83 Lindström K. *User's guide for the QPSNordic : general Nordic questionnaire for psychological and social factors at work*. KÃ¸benhavn: : Nordic Council of Ministers 2000.

84 Aiken LH, Clarke SP, Sloane DM, *et al.* Nurses' reports on hospital care in five countries. *Health Aff (Millwood)* 2001;**20**:43–53.

For peer review only

85 Notelaers G, De Witte H, Van Veldhoven M, *et al.* Construction and validation of the Short Inventory to Monitor Psychosocial Hazards. *Médecine du Trav Ergon Ergon* 2007;**Band XLIV**:11–7.

86 van Loon NM, Leisink PLM, Knies E, *et al.* Red Tape: Developing and Validating a New Job-Centered Measure. *Public Adm Rev* 2016;**76**:662–73. doi:10.1111/puar.12569

87 Schaufeli WB. Applying the Job Demands-Resources model: A “how to” guide to measuring and tackling work engagement and burnout. Published Online First: 2017. doi:10.1016/j.orgdyn.2017.04.008

88 Tremblay MA, Blanchard CM, Taylor S, *et al.* Work Extrinsic and Intrinsic Motivation scale its value for organizational psychology research. *Can J Behav Sci* 2009;**41**:213–26. doi:10.1037/a0015167

89 Bakker AB, Demerouti E. The Job Demands-Resources model: State of the art. *J Manag Psychol* 2007;**22**:309–28. doi:10.1108/02683940710733115

90 De Witte H, De Cuyper N, Handaja Y, *et al.* Associations between quantitative and qualitative job insecurity and well-being. *Int Stud Manag Organ* 2010;**40**:40–56. doi:10.2753/IMO0020-8825400103

91 Zapf D. Emotion work and psychological well-being. A review of the literature and some conceptual considerations. *Hum Resour Manag Rev* 2002;**12**:237–68. doi:10.1016/S1053-4822(02)00048-7

92 Van Veldhoven M, De Jonge J, Broersen S, *et al.* Specific relationships between psychosocial job conditions and job-related stress: A three-level analytic approach. *Work Stress* 2002;**16**:207–28. doi:10.1080/02678370210166399

93 Van den Broeck A, Vansteenkiste M, De Witte H, *et al.* Capturing autonomy, competence, and relatedness at work: Construction and initial validation of the Work-related Basic Need

For peer review only - http://bmjopen.bmj.com/site/about/guidelines.xhtml

Satisfaction scale. *J Occup Organ Psychol* 2010;**83**:981–1002. doi:10.1348/096317909X481382

94 Schaufeli WB. Engaging leadership in the job demands-resources model. *Career Dev Int* 2015;**20**:446–63. doi:10.1108/CDI-02-2015-0025

95 Shea CM, Jacobs SR, Esserman DA, *et al.* Organizational readiness for implementing change: A psychometric assessment of a new measure. *Implement Sci* 2014;**9**. doi:10.1186/1748-5908-9-7

96 Rapley T, Girling M, Mair FS, *et al.* Improving the normalization of complex interventions: Part 1 - Development of the NoMAD instrument for assessing implementation work based on normalization process theory (NPT) 17 Psychology and Cognitive Sciences 1701 Psychology. *BMC Med Res Methodol* 2018;**18**:133. doi:10.1186/s12874-018-0590-y

97 Finch TL, Girling M, May CR, *et al.* Improving the normalization of complex interventions: Part 2 - Validation of the NoMAD instrument for assessing implementation work based on normalization process theory (NPT) 17 Psychology and Cognitive Sciences 1701 Psychology. *BMC Med Res Methodol* 2018;**18**. doi:10.1186/s12874-018-0591-x

98 Creswell JW. Research Design: Qualitative, Quantitative, and Mixed Methods Approaches. *Res Des Qual Quant Mix methods approaches* 2014;**16**:189. doi:http://dx.doi.org/10.1016/j.math.2010.09.003

99 Ltd. QIP. NVivo. 2020.

100 Bowen GA. Document analysis as a qualitative research method. *Qual Res J* 2009;**9**:27–40. doi:10.3316/QRJ0902027

101 Blake H, Bermingham F, Johnson G, *et al.* Mitigating the psychological impact of covid-19 on healthcare workers: A digital learning package. *Int J Environ Res Public Health* 2020;**17**:2997. doi:10.3390/ijerph17092997

For peer review only

For peer review only - http://bmjopen.bmj.com/site/about/guidelines.xhtml

102 Sampaio F, Sequeira C, Teixeira L. Impact of COVID-19 outbreak on nurses' mental health: A prospective cohort study. *Environ Res* 2021;**194**:110620. doi:10.1016/J.ENVRES.2020.110620

103 De Kock JH, Latham HA, Leslie SJ, *et al.* A rapid review of the impact of COVID-19 on the mental health of healthcare workers: implications for supporting psychological well-being. *BMC Public Heal 2021 211* 2021;**21**:1–18. doi:10.1186/S12889-020-10070-3

104 Greenberg N, Weston D, Hall C, *et al.* Mental health of staff working in intensive care during Covid-19. *Occup Med (Chic Ill)* 2021;**71**:62–7. doi:10.1093/OCCMED/KQAA220

For peer review only

STANDARD PROTOCOL ITEMS: RECOMMENDATIONS FOR INTERVENTIONAL TRIALS

Supplementary File 1 - SPIRIT 2013 Checklist: Recommended items to address in a clinical trial protocol and related documents*

Section/item	Item No	Description	Addressed on page number
**Administrative information**			
Title	1	Descriptive title identifying the study design, population, interventions, and, if applicable, trial acronym	1
Trial registration	2a	Trial identifier and registry name. If not yet registered, name of intended registry	2, 17
	2b	All items from the World Health Organization Trial Registration Data Set	2, 6, 7
Protocol version	3	Date and version identifier	N/A
Funding	4	Sources and types of financial, material, and other support	20
Roles and responsibilities	5a	Names, affiliations, and roles of protocol contributors	1, 20
	5b	Name and contact information for the trial sponsor	20
	5c	Role of study sponsor and funders, if any, in study design; collection, management, analysis, and interpretation of data; writing of the report; and the decision to submit the report for publication, including whether they will have ultimate authority over any of these activities	20
	5d	Composition, roles, and responsibilities of the coordinating centre, steering committee, endpoint adjudication committee, data management team, and other individuals or groups overseeing the trial, if applicable (see Item 21a for data monitoring committee)	N/A

For peer review only

**Introduction**			
Background and rationale	6a	Description of research question and justification for undertaking the trial, including summary of relevant studies (published and unpublished) examining benefits and harms for each intervention	4-5
	6b	Explanation for choice of comparators	4-5
Objectives	7	Specific objectives or hypotheses	6
Trial design	8	Description of trial design including type of trial (eg, parallel group, crossover, factorial, single group), allocation ratio, and framework (eg, superiority, equivalence, noninferiority, exploratory)	6
**Methods: Participants, interventions, and outcomes**			
Study setting	9	Description of study settings (eg, community clinic, academic hospital) and list of countries where data will be collected. Reference to where list of study sites can be obtained	6
Eligibility criteria	10	Inclusion and exclusion criteria for participants. If applicable, eligibility criteria for study centres and individuals who will perform the interventions (eg, surgeons, psychotherapists)	6
Interventions	11a	Interventions for each group with sufficient detail to allow replication, including how and when they will be administered	9-12
	11b	Criteria for discontinuing or modifying allocated interventions for a given trial participant (eg, drug dose change in response to harms, participant request, or improving/worsening disease)	12
	11c	Strategies to improve adherence to intervention protocols, and any procedures for monitoring adherence (eg, drug tablet return, laboratory tests)	N/A
	11d	Relevant concomitant care and interventions that are permitted or prohibited during the trial	N/A
Outcomes	12	Primary, secondary, and other outcomes, including the specific measurement variable (eg, systolic blood pressure), analysis metric (eg, change from baseline, final value, time to event), method of aggregation (eg, median, proportion), and time point for each outcome. Explanation of the clinical relevance of chosen efficacy and harm outcomes is strongly recommended	12-16
Participant timeline	13	Time schedule of enrolment, interventions (including any run-ins and washouts), assessments, and visits for participants. A schematic diagram is highly recommended (see Figure)	8

For peer review only - http://bmjopen.bmj.com/site/about/guidelines.xhtml

Sample size	14	Estimated number of participants needed to achieve study objectives and how it was determined, including clinical and statistical assumptions supporting any sample size calculations	7
Recruitment	15	Strategies for achieving adequate participant enrolment to reach target sample size	7, 12

Methods: Assignment of interventions (for controlled trials)

Allocation:

Sequence generation	16a	Method of generating the allocation sequence (eg, computer-generated random numbers), and list of any factors for stratification. To reduce predictability of a random sequence, details of any planned restriction (eg, blocking) should be provided in a separate document that is unavailable to those who enrol participants or assign interventions	7-8
Allocation concealment mechanism	16b	Mechanism of implementing the allocation sequence (eg, central telephone; sequentially numbered, opaque, sealed envelopes), describing any steps to conceal the sequence until interventions are assigned	7-8
Implementation	16c	Who will generate the allocation sequence, who will enrol participants, and who will assign participants to interventions	7-8
Blinding (masking)	17a	Who will be blinded after assignment to interventions (eg, trial participants, care providers, outcome assessors, data analysts), and how	N/A
	17b	If blinded, circumstances under which unblinding is permissible, and procedure for revealing a participant's allocated intervention during the trial	N/A

Methods: Data collection, management, and analysis

Data collection methods	18a	Plans for assessment and collection of outcome, baseline, and other trial data, including any related processes to promote data quality (eg, duplicate measurements, training of assessors) and a description of study instruments (eg, questionnaires, laboratory tests) along with their reliability and validity, if known. Reference to where data collection forms can be found, if not in the protocol	8, 11-16
	18b	Plans to promote participant retention and complete follow-up, including list of any outcome data to be collected for participants who discontinue or deviate from intervention protocols	12

Data management	19	Plans for data entry, coding, security, and storage, including any related processes to promote data quality (eg, double data entry; range checks for data values). Reference to where details of data management procedures can be found, if not in the protocol	___17_________
Statistical methods	20a	Statistical methods for analysing primary and secondary outcomes. Reference to where other details of the statistical analysis plan can be found, if not in the protocol	_14-16________
	20b	Methods for any additional analyses (eg, subgroup and adjusted analyses)	_14-16________
	20c	Definition of analysis population relating to protocol non-adherence (eg, as randomised analysis), and any statistical methods to handle missing data (eg, multiple imputation)	___14________
**Methods: Monitoring**			
Data monitoring	21a	Composition of data monitoring committee (DMC); summary of its role and reporting structure; statement of whether it is independent from the sponsor and competing interests; and reference to where further details about its charter can be found, if not in the protocol. Alternatively, an explanation of why a DMC is not needed	_ ___N/A____
	21b	Description of any interim analyses and stopping guidelines, including who will have access to these interim results and make the final decision to terminate the trial	__ _N/A_____
Harms	22	Plans for collecting, assessing, reporting, and managing solicited and spontaneously reported adverse events and other unintended effects of trial interventions or trial conduct	_ __N/A_____
Auditing	23	Frequency and procedures for auditing trial conduct, if any, and whether the process will be independent from investigators and the sponsor	__ _N/A_____
**Ethics and dissemination**			
Research ethics approval	24	Plans for seeking research ethics committee/institutional review board (REC/IRB) approval	__2, 17________
Protocol amendments	25	Plans for communicating important protocol modifications (eg, changes to eligibility criteria, outcomes, analyses) to relevant parties (eg, investigators, REC/IRBs, trial participants, trial registries, journals, regulators)	__17__________

Consent or assent	26a	Who will obtain informed consent or assent from potential trial participants or authorised surrogates, and how (see Item 32)	12
	26b	Additional consent provisions for collection and use of participant data and biological specimens in ancillary studies, if applicable	N/A
Confidentiality	27	How personal information about potential and enrolled participants will be collected, shared, and maintained in order to protect confidentiality before, during, and after the trial	suppl. material #2
Declaration of interests	28	Financial and other competing interests for principal investigators for the overall trial and each study site	20
Access to data	29	Statement of who will have access to the final trial dataset, and disclosure of contractual agreements that limit such access for investigators	17
Ancillary and post-trial care	30	Provisions, if any, for ancillary and post-trial care, and for compensation to those who suffer harm from trial participation	N/A
Dissemination policy	31a	Plans for investigators and sponsor to communicate trial results to participants, healthcare professionals, the public, and other relevant groups (eg, via publication, reporting in results databases, or other data sharing arrangements), including any publication restrictions	17
	31b	Authorship eligibility guidelines and any intended use of professional writers	17
	31c	Plans, if any, for granting public access to the full protocol, participant-level dataset, and statistical code	17
**Appendices**			
Informed consent materials	32	Model consent form and other related documentation given to participants and authorised surrogates	12, suppl. material #2
Biological specimens	33	Plans for collection, laboratory evaluation, and storage of biological specimens for genetic or molecular analysis in the current trial and for future use in ancillary studies, if applicable	N/A

*It is strongly recommended that this checklist be read in conjunction with the SPIRIT 2013 Explanation & Elaboration for important clarification on the items. Amendments to the protocol should be tracked and dated. The SPIRIT checklist is copyrighted by the SPIRIT Group under the Creative Commons "Attribution-NonCommercial-NoDerivs 3.0 Unported" license.

BMJ Open

A workplace organizational intervention to improve hospital nurses' and physicians' mental health: study protocol for the Magnet4Europe wait-list cluster randomized controlled trial

Journal:	*BMJ Open*
Manuscript ID	bmjopen-2021-059159.R1
Article Type:	Protocol
Date Submitted by the Author:	24-Jun-2022

Complete List of Authors:	Sermeus, Walter; Katholieke Universiteit Leuven, Department of Public Health and Primary Care<br>Aiken, Linda; University of Pennsylvania, Center for Health Outcomes and Policy Research<br>Ball, Jane; University of Southampton, Faculty of Health Sciences<br>Bridges, Jackie; University of Southampton, Faculty of Health Sciences<br>Bruyneel, Luk; Katholieke Universiteit Leuven, Department of Public Health and Primary Care<br>Busse, Reinhard; Technical University of Berlin, Health Care Management<br>De Witte, Hans; Katholieke Universiteit Leuven, Occupational & Organisational Psychology and Professional Learning<br>Dello, Simon; Katholieke Universiteit Leuven, Department of Public Health and Primary Care<br>Drennan, Jonathan; University College Cork National University of Ireland<br>Eriksson, Lars E; Karolinska Institutet, Department of Learning, Informatics, Management and Ethics<br>Griffiths, Peter; University of Southampton, Faculty of Health Sciences<br>Kohnen, Dorothea; KU Leuven Faculty of Psychology and Educational Sciences, Work, Organisational, and Personnel Psychology<br>Köppen, Julia; Technical University of Berlin, Department of Healthcare Management<br>Lindqvist, Rikard; Karolinska Institutet<br>Maier, Claudia; Technische Universitat Berlin, Department of Healthcare Management<br>McHugh, Matthew; University of Pennsylvania, School of Nursing, Center for Health Outcomes and Policy Research<br>McKee, Martin; London School of Hygiene & Tropical Medicine<br>Rafferty, Anne Marie; King's College London, Florence Nightingale School of Nursing and Midwifery<br>Schaufeli, Wilmar; Katholieke Universiteit Leuven; Utrecht University, Department of Psychology<br>Sloane, Douglas; University of Pennsylvania<br>Alenius, Lisa Smeds; Karolinska Institutet<br>Smith, Herbert; University of Pennsylvania,<br>Magnet4Europe consortium, Magnet4Europe consortium; Magnet4Europe consortium

**Primary Subject Heading**:	Health policy
Secondary Subject Heading:	Health policy, Health services research, Mental health, Public health, Nursing
Keywords:	Health policy < HEALTH SERVICES ADMINISTRATION & MANAGEMENT, Protocols & guidelines < HEALTH SERVICES ADMINISTRATION & MANAGEMENT, MENTAL HEALTH, Human resource management < HEALTH SERVICES ADMINISTRATION & MANAGEMENT, Organisation of health services < HEALTH SERVICES ADMINISTRATION & MANAGEMENT

SCHOLARONE™
Manuscripts

A WORKPLACE ORGANIZATIONAL INTERVENTION TO IMPROVE HOSPITAL NURSES' AND PHYSICIANS' MENTAL HEALTH: STUDY PROTOCOL FOR THE MAGNET4EUROPE WAIT-LIST CLUSTER RANDOMIZED CONTROLLED TRIAL

Authors

Sermeus Walter, KU Leuven, Belgium (first author)

Aiken Linda, University of Pennsylvania, USA (first author)

Ball Jane, University of Southampton, UK

Bridges Jackie, University of Southampton, UK

Bruyneel Luk, KU Leuven, Belgium

Busse Reinhard, Technical University Berlin, Germany

De Witte Hans, KU Leuven, Belgium

Dello Simon, KU Leuven, Belgium

Drennan Jonathan, University College Cork, Ireland

Eriksson Lars E., Karolinska Institutet, Sweden

Griffiths Peter, University of Southampton, UK

Kohnen, Dorothea, KU Leuven, Belgium

Köppen Julia, Technical University Berlin, Germany

Lindqvist Rikard, Karolinska Institutet, Sweden

Maier Claudia, Technical University Berlin, Germany

McHugh Matthew, University of Pennsylvania, USA

McKee Martin, London School of Hygiene and Tropical Medicine, UK

Rafferty Anne Marie, King's College London, UK

Schaufeli Wilmar, KU Leuven, Belgium

Sloane Douglas, University of Pennsylvania, USA

Smeds Alenius Lisa, Karolinska Institutet, Sweden

Smith Herbert, University of Pennsylvania, USA

For the Magnet4Europe consortium

Corresponding author: Walter Sermeus, Leuven Institute for Healthcare Policy, KU Leuven, Kapucijnenvoer 35, 3000 Leuven, Belgium; e-mail: walter.sermeus@kuleuven.be

WORD COUNT

Words: 4992

For peer review only - http://bmjopen.bmj.com/site/about/guidelines.xhtml

ABSTRACT

Introduction The increasing burden of mental distress reported by healthcare professionals is a matter of serious concern and there is a growing recognition of the role of the workplace in creating this problem. Magnet® hospitals, a model shown to attract and retain staff in US research, creates positive work environments that aim to support the well-being of healthcare professionals.

Methods and analysis Magnet4Europe is a cluster randomized controlled trial, with wait list controls, designed to evaluate the effects of organizational redesign, based on the Magnet® model, on nurses' and physicians' well-being in general acute care hospitals, using a multicomponent implementation strategy. The study will be conducted in more than 60 general acute care hospitals in Belgium, England, Germany, Ireland, Norway, and Sweden. The primary outcome is burnout among nurses and physicians, assessed in longitudinal surveys of nurses and physicians at participating hospitals. Additional data will be collected from them on perceived work environments, patient safety, and patient quality of care and will be triangulated with data from medical records, including case mix adjusted in-hospital mortality. The process of implementation will be evaluated using qualitative data from focus group and key informant interviews.

Ethics and dissemination This study was approved by the Ethics Committee Research UZ/KU Leuven, Belgium; additionally, ethics approval is obtained in all other participating countries either through a central or decentral authority. Findings will be disseminated at conferences, through peer reviewed manuscripts, and via social media.

Trial registration number ISRCTN10196901.

For peer review only

For peer review only - http://bmjopen.bmj.com/site/about/guidelines.xhtml

STRENGTHS AND LIMITATIONS OF THIS STUDY

- Magnet4Europe uses a cluster randomized controlled trial design with wait-list controls in over 60 hospitals in six European countries.
- A multicomponent strategy is used to support implementation of a complex intervention at the hospital level, aimed to improve clinician well-being.
- The Magnet4Europe intervention is tailored to European hospitals, facilitating implementation and transferability without disrupting clinical practice.
- Contextual factors such as different health systems within and across countries may impact the standardized Magnet4Europe intervention and unintentionally lead to variation in the intervention and outcomes.
- Contamination of the wait-list control group cannot be ruled out.
- Embedding a complex intervention at hospital-level takes time and any effect might become apparent only after the study ends.

For peer review only

For peer review only - http://bmjopen.bmj.com/site/about/guidelines.xhtml

INTRODUCTION

Mental health and well-being are high priorities for the European Union (EU).[1] Mental health conditions account for 22% of the EU's disease burden measured in Years Lived with Disability, thus imposing a significant burden on individuals, society and the economy.[2] Health workers experience greater levels of job-related burnout and other mental health disorders than those in other sectors.[3,4] An earlier European study of working conditions in European hospitals, identified high rates of job dissatisfaction and burnout, with burnout rates among nurses varying from 10% to 78%[5,6] while the corresponding figures from studies of physicians ranged from 25% to 60%, varying among organizations and medical specialties.[7–10] Burnout in the healthcare workforce not only impacts on those experiencing it, leading to depression, substance abuse and even suicide,[11–15] but is also associated with worse patient outcomes, lower patient satisfaction,[6] medical errors,[16] reduced quality and safety[17] and reduced efficiency of hospitals.[18] The resulting lost productivity, combined with current recruitment challenges, further threatens the already overstretched health workforce, widening the gap between provision of health services and population needs.[19]

This situation has been exacerbated by the COVID-19 pandemic,[20] increasing anxiety and stress among clinicians[21] and further impacting on health workers' mental health.[22–24]

Effective, affordable and sustainable interventions to improve health professionals' mental health and well-being are essential if we are to interrupt the vicious cycle of high burnout, worsening mental health, lost productivity, and unsafe care.[25] Interventions to prevent burnout can be divided into two types: firstly, psychological interventions aimed at individual level and secondly workplace redesign. There is only limited evidence of the impact of individual psychological approaches to improve resilience and coping skills (e.g. mindfulness).[26] The second approach, redesigning the organizational environment in which health professionals provide care, aims to reduce modifiable sources of clinician stress and burnout, reinforce clinicians' perceptions of well-being, and enhance their autonomy and control over the conditions of their work. There is substantial research pointing to organizational redesign as the most promising of these two approaches.[27]

For peer review only - http://bmjopen.bmj.com/site/about/guidelines.xhtml

The finding that the causes underlying burnout in nurses and physicians are similar and, especially, are rooted within the work environment[28–31] provides a compelling rationale for prioritizing the redesign of hospital environments to address this issue. In the 1980s, hospitals in the US were faced with high nurse turnover,[32,33] low retention, and increasing early retirement rates.[34] Despite the general nursing shortage, some US hospitals - later referred to as Magnet® hospitals - were more successful in recruiting and retaining nurses by creating a positive work environment. Features characterizing these hospitals included flat decentralized organizational structures, empowering frontline staff in decision making and transformational leadership.[35] The original characteristics of these hospitals[36] were developed into fourteen forces of Magnetism and later configured into five components of the Magnet® model that was used to establish the criteria for Magnet® designation.[37] A robust body of evidence, primarily stemming from US hospitals, documents the association of the Magnet® model with improved well-being of staff including lower burnout, higher job satisfaction, lower intent to leave their job,[38,39] in addition to positive financial outcomes for organizations,[40,41] higher levels of patient satisfaction,[42,43] and improved clinical outcomes.[44,45] Longitudinal panel studies have found that hospitals that follow the Magnet® model improve their work environments, well-being of staff and patient outcomes, compared to other hospitals. Two international pilots tested the transferability of the Magnet® model, demonstrating the feasibility of achieving positive results outside the USA. One, in England, focused primarily on the implementation of the Magnet® process.[46] The other, a four-hospital intervention with accompanying evaluation in Russia and Armenia, used the Magnet® model with twinning with Magnet® recognized hospitals.[47] In both cases, the intervention hospitals improved their work environments substantially from their baselines within the two-year intervention and achieved better outcomes for staff, such as reduced dissatisfaction, intent to leave, and emotional exhaustion, while perceived quality of care improved. Despite the large body of evidence and recent uptake in two European hospitals, Magnet® organizational redesign initiatives have not been adopted widely in Europe.

For peer review only - http://bmjopen.bmj.com/site/about/guidelines.xhtml

The aim of the Magnet4Europe project is to evaluate the ability of organizational redesign in European hospitals to improve nurses' and physicians' mental health and well-being. The organizational redesign intended in Magnet4Europe is a system level intervention, targeting the hospital as a whole on the level of the organization. Through co-creation and stakeholder co-designed adaptations, the Magnet® model is adapted to the European context, providing the first element and basis for the Magnet4Europe intervention. In addition to this first component, one-to-one twinning with Magnet® recognized hospitals, learning collaboratives and a critical mass of hospitals are also introduced into the intervention. While the Magnet4Europe intervention shares common elements with other organizational interventions, the combination of all the above components, targeting two major clinical professions – both nurses and physicians – and its primary focus on the work environment as levers for change differentiate the Magnet4Europe intervention from any other organizational intervention and make it unique in its kind.[48]

METHODS AND ANALYSIS

Study design and setting

Magnet4Europe will use a usual-practice cluster randomized controlled trial with wait-list controls in a nested mixed-methods evaluation. The study protocol for this study follows "the Standard protocol items: recommendations for intervention trials 2013 statement" (SPIRIT; supplementary file 1).[49] The setting will be acute general hospitals in six European countries, including both European Union member states (Belgium, Germany, Ireland and Sweden) and non-European member states (England and Norway). These six countries also represent two dominant types of health systems, Bismarck and Beveridge,[50] that vary with regard to the extent of national government intervention in health services delivery, something that may be a factor in uptake of innovation.

Eligibility

European hospitals are eligible to participate if: (1) no Magnet® designation by the American Nursing Credentialing Centre (ANCC) had been acquired in the past or at the time of the start of the intervention, (2) bed size ≥150, and (3) the hospital is focused on acute care for adults, including wards

For peer review only - http://bmjopen.bmj.com/site/about/guidelines.xhtml

in the area of internal medicine and/or surgery. Excluded are highly specialized hospitals, e.g., psychiatric hospitals, tropical medicine, or paediatrics.

Within each hospital, registered nurses, and physicians (including residents) will be eligible to participate in the quantitative and qualitative evaluation of the intervention if they: (1) have direct patient contact, (2) meet the minimum qualifications as stipulated by Directive 2013/55/EU amending Directive 2005/36/EC on the recognition of professional qualifications, and (3) work on adult inpatient units including intensive care units (ICU) and the emergency room (ER). Nurses and physicians working in specialized units such as neonatology, paediatrics, obstetrics, psychiatry, operating room, pathology, microbiology, radiology, and medical imaging will be excluded.

Study sample

A two-phased non-probability strategy will be used to sample organizations at the hospital level. First, in February and March 2019, principal investigators in the six European countries contacted eligible hospitals (see below) within those countries to consider taking part in the study using various recruitment strategies. Hospitals were informed of the Magnet4Europe objectives and methodology and were invited to show their interest in participating by submitting a letter of intent by March 2019. In spring 2020, participating hospitals (some of the sites and other new ones), were asked to confirm their commitment by signing an agreement committing to participate for the full duration of the study period and adhere to the Magnet4Europe protocol.

Sample size will be determined based on the power calculation for a cluster randomized controlled trial with before-and-after measures (CRT-BA).[51] The calculated design effect is based on average cluster size and the intraclass correlation coefficient (ICC), a measure of the amount of dependency among observations within classes (i.e. hospitals in our study).[51,52] Power calculation will be performed for the primary outcome of interest, burnout, measured by the emotional exhaustion subscale of the Maslach Burnout Inventory [53]. The estimated prevalence rate of emotional exhaustion is 30% based upon the team's earlier RN4CAST study.[6] In order to detect a 20% relative

For peer review only - http://bmjopen.bmj.com/site/about/guidelines.xhtml

reduction (i.e. a 6 percentage point reduction) in burnout with a power of 80% and an alpha of 5%, an average cluster size of 200 and an ICC of 0.025,[54] this study will require 10,253 health professionals in 51 clusters per measurement occasion (at endpoint of the study).

Based on the power calculation, we aim to include 60 general acute care hospitals in six countries (Belgium, England, Germany, Ireland, Norway, and Sweden) and recruit at least 200 professionals per hospital.

Randomization

Randomization to intervention and control group is at the hospital level. Within each country hospitals will be matched on three characteristics, (1) bed size, (2) teaching status and (3) high technology, using a Euclidian distance matrix based on the smallest within pair covariate distance. Subsequently, simple random sampling - allowing for selection with equal probability - will be used to allocate one hospital within each matched pair of hospitals to the immediate intervention group or the wait list control group. Both immediate intervention group and wait list controls will be informed which group they have been allocated to. Given the characteristics of the intervention in which active participation of the hospitals is required, blinding to the intervention will not be possible.

Intervention

A multicomponent intervention comprising organizational redesign of hospitals will be implemented in both arms of the study. The intervention was launched in the immediate intervention group in month 10 of the project (October 2020) and will have a total duration of 31 months. The intervention in the usual-practice wait-list control group will be initiated in month 17 (May 2021) and will last for 24 months (Figure 1).

The intervention consists of five distinct components: The Magnet blueprint, one-to-one twinning between European hospitals and US Magnet designated hospitals, learning collaboratives involving group meetings to share best practices, critical mass, and communication and feedback.

For peer review only - http://bmjopen.bmj.com/site/about/guidelines.xhtml

For peer review only

The first part of the intervention comprises the elements outlined in the Magnet® manual of organizational redesign. This contains a step-by-step blueprint through the five overarching Magnet® components: (1) structural empowerment of clinical staff; (2) transformational leadership; (3) exemplary and evidence-based professional practice; (4) new knowledge, innovations, and improvements; and (5) empirical outcomes. These principles serve as continuous feedback loops as to whether organizational changes are producing the intended outcomes for health professionals and patients. The manual provides definitions of the principles and gives examples of evidence-based indicators that reflect progress toward achieving them. Using the Magnet4Europe Gap Analysis Tool (adapted from the ANCC Magnet® Gap Analysis tool), each participating European hospital will, in collaboration with their Magnet® twinning partner, perform a gap analysis that will illuminate the gap between the as-is situation and the aspirational organizational features as delineated in the Magnet® blueprint. The tool should be used for initial and continuous assessment of workplace redesign. This will increase the ability of hospitals to identify and prioritize learning and infrastructure needs, while also identifying initiative-wide gaps and exemplars for systematic improvement and recognition. Drawing on the results of the gap analysis, each hospital will be responsible for tailoring and individualizing the interventions to their hospital-specific context and developing a concrete, written action plan that will be monitored and executed during the course of the intervention period. Results of the gap analysis and the effect of tailored interventions will serve as feedback to enhance learning.

The second intervention component consists of one-to-one twinning between European hospitals aspiring to implement the Magnet® model and longstanding US Magnet® designated hospitals. This seeks to promote capacity building through transfer of knowledge, skills, tools, technologies and best practices, thereby supporting positive reciprocal organizational changes.[55] An equal number of U.S. Magnet® hospitals – recognized for having an excellent work environment – will be recruited; allowing for a 1:1 twinning ratio of EU intervention hospitals with U.S. Magnet Hospitals. The twinning pairs will meet every two weeks virtually and two face-to-face mentoring sessions are planned each year when travel is safe. Face-to-face meetings between both parties allow for a profound, impact- and

For peer review only - http://bmjopen.bmj.com/site/about/guidelines.xhtml

meaningful experience that maximises the effect of the capacity building activities facilitating the intended organizational redesign.

Third, learning collaboratives will take place throughout the duration of the project and will be accessed and attended by all European hospitals in which the intervention is taking place at that point in time. The learning collaboratives will also involve policymakers so as to promote sustainability. Learning collaboratives build on the concept of communities of practice,[56] involving people coming together to engage in a process of learning in a shared domain of interest. These will be held monthly using remote means of communication with the possibility of in-person meetings annually when travel is safe. Effective elements of collaboratives, i.e. learning sessions, action periods and a collaborative extranet, will be implemented.[57–59]

Fourth is the creation of a critical mass of participating hospitals promoting innovation, attracting public interest, engaging stakeholders, and fostering replication and scaling of the intervention. Achieving a critical mass is essential for sustainability and scale-up of the intervention.[60] Critical mass shifts the context from one in which the change activities are unusual to one in which they are the norm. This long-standing principle in organizational sociology suggests that organizations become more open to and actively seek out change as the intervention gains legitimacy among peer institutions; this is particularly true in complex workplaces such as those found in health care.[61,62] Critical mass also maximizes the network exchanges and opportunities for feedback that arise out of other elements of our intervention such as the learning collaborative. This is supported by recent experience with the Magnet® model.[63,64]

A fifth component of the intervention is to provide regular survey feedback to hospitals on clinicians' reports on work conditions and well-being on the hospital level, separately for physicians and nurses.[65] Hospitals will be anonymized using a randomly generated letter code allowing each hospital to identify themselves, but not any of the other hospitals. Data will only be reported in aggregated form and will not highlight a single individual's data.

For peer review only - http://bmjopen.bmj.com/site/about/guidelines.xhtml

Evaluation of the effect of the intervention

A mixed-methods evaluation will be conducted to examine the effect of the intervention, including quantitative and qualitative components.

The guiding framework for our quantitative evaluation is the Quality Health Outcomes Model (QHOM) – as adapted by Aiken and colleagues[66] – based on a conceptual framework that sets out mechanisms relating organizational attributes to clinician and patient outcomes. Their model is theoretically founded on Donabedian's early conception of associations between structures, processes and outcomes[67] including the dynamism between model components that was proposed in the Health Quality Outcomes Model.[68] The Job Demands-Resources (JD-R) model also informed the choice of survey items, seeking to understand the intervention's impact on the imbalance between demands on the clinician and the resources available to clinicians to deal with these demands.[69] Job demands refer to aspects of the job that require sustained physical or mental effort and are associated with certain physiological and psychological costs. Job resources describe aspects of the job that are related to achieving work goals, reducing job demands and the associated physiological and psychological costs, and stimulating personal growth and development.

The qualitative part of the study aims first, to evaluate the implementation process, adaptation, reach and quality as well as mechanisms of impact manifested in participants' responses, mediators, unexpected pathways and consequences; and second, to explore barriers and enablers of the implementation and the experiences of the change process from the perspectives of health professionals and persons involved in leading the implementation.[70]

Quantitative evaluation

Quantitative data collection

Quantitative data collection across countries is standardized using an online data collection platform. Quantitative survey data will be collected at three points in time, i.e., at baseline (T0) in months 11-12 of the project timeline, at T1 (months 28-29) and T2 (months 41-42) (Figure 1) during an 8-week period. An open cohort design will be employed in which all eligible nurses and physicians are invited to

For peer review only - http://bmjopen.bmj.com/site/about/guidelines.xhtml

participate in each of these three measurement points (T0 – T2). Nurses and physicians will be recruited within each hospital at baseline and will be asked to participate in surveys with repeated measures taken over the three points in time allowing partially longitudinal follow-up of a cohort of individuals. Initial non-participants and new employees will be invited to join the study at later measurement points. Regular reminders will be sent to any clinicians (registered on the platform) through the online data collection platform who have not or only partially completed the survey. Individual informed consent for participation will be obtained by the researchers at the first contact (supplementary file 2). Each record of informed consent is retained on the online platform.

Quantitative measures

The main survey for nurses and physicians contains a core set of outcomes and main explanatory and process variables directly linked to the Magnet4Europe Magnet® Gap Analysis Tool. All study measures are based on validated instruments.

All survey items will be translated in Dutch, English, German, Norwegian and Swedish. When available, validated translations of instruments will be used. If no validated translation is available, a pre-specified translation procedure will be applied. First, a forward translation will be performed by a researcher involved in this research; the language in which the item is translated is the native language of the researcher. Secondly, other researchers within the country research group will thoroughly assess the forward translation in multiple phases until consensus is researched on each item.

Primary outcome

Burnout is the primary outcome measure and will be measured on individual level using the 9-item Emotional Exhaustion (EE) subscale of the Maslach Burnout Inventory (MBI).[71] In addition, the Burnout Assessment Tool (BAT), a 12-item novel self-report questionnaire will also be used to measure burnout.[72–74] The combination of both instruments to assess burnout, allows for: (1) a direct comparison of levels of emotional exhaustion with previous studies in the medical field (using the EE-subscale of MBI); (2) an up-to-date assessment of the more comprehensive burnout syndrome, as well

For peer review only - http://bmjopen.bmj.com/site/about/guidelines.xhtml

For peer review only

as a reliable estimation of the number of respondents with severe burnout symptoms and a comparison with the level of burnout of the national workforce.

Covariates

Secondary outcome measures include work engagement (UWES-3),[75] job satisfaction,[76,77] depression (PHQ-2),[78] anxiety (GAD-2),[79] general health (SF-8),[80] sleep quality (PSQ),[81] intent to leave the hospital,[76,77] absenteeism and presenteeism (HPQ),[14] workability, work-life conflict, team commitment, organizational commitment,[82] and whether clinicians would recommend the hospital.

Core exploratory variables include work environment (PES-NWI),[83] various measures of staffing and workload (NPQS),[84] care left undone,[85] operational failures, emotional demands (SIMPH),[86] red tape,[87] role conflicts (NPQS), job control, skill use, value congruence, performance feedback, opportunities for learning and development[88] and intrinsic motivation (WEIMS).[89]

Supplementary survey items are used to test more specific hypotheses.[69,90] These items contain measures on qualitative job insecurity [91], emotional dissonance,[92] task variety (QEEW),[93] and role clarity (NQPS),[84] basic need satisfaction,[94] and engaging leadership.[95]

Productivity measures consist of staff turnover rate, sickness absence rate, number of vacancies, and the use of agency staff - measured at organization level - complemented by previously mentioned secondary outcome measures, e.g., absenteeism and presenteeism are measured at individual level by means of a survey. Productivity measures evaluated at the level of the organization are evaluated over the period of one calendar year. The timeframe to evaluate absenteeism on individual level is 28 days, participants are asked to indicate the number of scheduled work days they have missed during the past 28 days. The timeframe to evaluate presenteeism on individual level is one year.

Data analysis

Regression models to estimate the association between the intervention and outcomes will include fixed effects of time, intervention, and intervention by time. Hospitals represent higher order units of

For peer review only - http://bmjopen.bmj.com/site/about/guidelines.xhtml

analysis, and clinicians the lower order unit of analysis. Countries will be treated as fixed effects. Repeated measures on nurses and physicians will also be appropriately modelled. More complex multilevel structural models will be employed to examine indirect and moderated associations between the process measures which we would expect to respond to the intervention and our outcome measures. All analyses will be conducted according to intention-to-treat. Data on hospital characteristics (e.g., size, teaching status) and clinician (e.g., gender, age) will be collected and used to adjust for potential confounders in the regression analyses. Statistical significance will be assessed at the 5% level. Occasional missing data will be limited by the electronic survey implementation using a force response option for each item. Data analysis will be conducted using SAS software, version 9.4 (and subsequent releases) of the SAS system for windows (SAS Institute Inc., Cary, NC, USA).

Process evaluation

Qualitative data collection

Data will be collected on context, implementation, and mechanisms of impact. Based on the results at baseline measurement, a purposive sample of two hospitals per country will be selected from the immediate intervention group for case studies to explore different aspects of the implementation process. Hospitals with the lowest burnout scores among clinicians will be contrasted with hospitals with the highest to explore potential differences in motivation and ambition in the hospitals. In total, four qualitative data collection periods are planned throughout the project (Figure 1). Two focus group interviews (one with 6-8 nurses, one with 6-8 physicians) will be conducted in each of the two case hospitals (per country) at one point in time (M34-35). This will generate 20 focus groups in total across all countries. A purposive sampling approach is used to recruit participants. We will invite physicians and nurses who work in direct patient care in clinical areas targeted by the Magnet intervention and most closely involved in implementing change. By doing so, we will aim for diversity within each staff group in terms of clinical areas and length of clinical experience. If focus groups cannot be convened (for example, recruitment difficulties), then each focus group will be replaced with 2 to 3 one-to-one interviews with similar staff types to the target focus group sample. Depending on practicalities, this

For peer review only - http://bmjopen.bmj.com/site/about/guidelines.xhtml

may result in a dataset that is generated from focus groups and/or one-to-one interviews. A minimum of 40 and maximum of 160 individuals will be involved in this part of the study.

In addition to the focus groups, two semi-structured individual interviews will be conducted at three time-points (months 23, 34 and 44) in each of the two case hospitals (per country). Persons directly involved in leading the implementation, i.e., the Magnet4Europe intervention coordinator, as well as another member of the interdisciplinary Magnet council will be invited to participate. This will lead to 60 interviews in total (across countries) over the course of the project. Where possible, we aim to interview the same two people across all time-points. If individuals leave the study, we will aim to recruit a replacement with a similar role and professional background. We will also aim for maximum diversity in professional backgrounds, e.g., one from nursing, one from medicine. A minimum of 30 and a maximum of 60 individuals will be involved in this part of the study.

To complement the qualitative evaluation, the 12-item Organizational Readiness for Implementing Change scale (ORIC)[96] and the 23-item Normalization Measure Development Questionnaire (NoMAD)[97,98] will be used to explore the readiness for change and the extent to which Magnet is integrated and sustained in the hospital over time. This will be done after the start of the intervention and at various time points. Magnet4Europe intervention coordinators and all other members of the interdisciplinary Magnet4Europe council in all hospitals will be invited to respond to the surveys. A minimum of 54 and a maximum of 324 individuals across all countries will be involved in this part of the study at each time point. If individuals leave and are replaced, their replacements will be invited to complete the survey on the next occasion.

Qualitative data analysis

Analysis of the qualitative data generated through focus groups and individual interviews will be guided by the Framework Analysis approach.[99] Data collection and analysis will be conducted by researchers in each of the Magnet4Europe countries, using a common coding template which will be developed based on initial themes. International meetings will be held regularly to discuss analysis and

For peer review only - http://bmjopen.bmj.com/site/about/guidelines.xhtml

findings. NVivo 12 (and subsequent releases) will be used for management and analysis of qualitative data.[100]

Data generated through collection of policy documents and general hospital information about context will be analysed through content analysis and/or discourse analysis[101] to explore different aspects of the organizational context in relation to the implementation process.

In the process evaluation we will analyse how organizational context links to the impact and sustainability of the Magnet programme. Emerging themes from the qualitative analysis will be used, together with the results from descriptive analyses of our quantitative measurements of readiness for change and of implementation normalization (NoMAD instrument), to explain variations in implementation between and within individual settings over time (Table 1).

Table 1 - Magnet4Europe Implementation Evaluation

**Context**
▪ General information about the hospital (e.g., type of hospital, size, organizational models, professional structure)<br>▪ Hospital policy documents (e.g., about staffing, work environment, leadership development, organizational plans)<br>▪ General country information (e.g., relevant legislation, financing of the healthcare sector)
**Implementation**
▪ Results from the Magnet4Europe Magnet® Gap Analysis Tool and the work plan of each hospital<br>▪ Implementation process survey data<br>▪ Data from interviews and focus groups
**Mechanisms of impact**
▪ Hospital policy documents (e.g., staffing, work environment, leadership development, organizational plans)<br>▪ Implementation process survey data<br>▪ Data from interviews and focus groups

For peer review only - http://bmjopen.bmj.com/site/about/guidelines.xhtml

Patient and public involvement

Patients and/or public were not involved in the design or development of research questions of this study.

ETHICS AND DISSEMINATION

Ethics

This study was approved by the Ethics Committee Research UZ/KU Leuven, Belgium; additionally, ethics approval is obtained in all other participating countries either through a central or decentral authority, to conduct nurse surveys as well as collect qualitative data. Any protocol amendments will be promptly reported to all relevant parties. An Ethical Advisory Committee was installed at the start of project and includes an independent ethics advisor with documented expertise to monitor ethics issues. The study is registered in the ISRCTN registry (ISRCTN10196901).

Dissemination

Magnet4Europe has a strong focus on disseminating evidence on the impact of work environment on mental health of health professionals. Findings will be published in peer reviewed journals and through other channels designed to reach a diverse community of researchers, practitioners, and other stakeholders. Gold open access will be used for the key findings of the study to ensure maximum exposure to a practice community. Beyond the funded life of the project the aim is to maximize accessibility by fully exploiting the opportunity for green open access and, where possible, identifying additional funds to support further gold open access publishing. Data management is compliant in line with the European Commission's Horizon 2020 Data Management Plan guidelines and FAIR-principles.

For peer review only

DISCUSSION

This paper describes the theoretical foundations, conceptual model, study design and methodological approaches of the EU H2020 Magnet4Europe project, which aims to evaluate the transfer, implementation, scale up and cost-effectiveness of the - Magnet® model of organizational redesign in a European context as a system-level approach to improve clinician well-being. Mental health and well-being is among one of the highest priorities of the public health agenda in the European Union.[1] This is based on the growing awareness and recognition of the magnitude of mental health problems and the burden on individuals, society, and economy. With the extraordinary conditions created by the coronavirus pandemic (COVID-19), this issue has taken an even more central role in the policy landscape.[102] COVID-19 is expected to have psychological impacts for health professional, in particular frontline workers that will be sustained over time.[103–106]

The research activities conducted within the project are expected to improve significantly the work environment of health professionals in hospitals, especially after the COVID-19 pandemic. In particular, Magnet4Europe proposes an organizational intervention, targeting the root of many of the problems (i.e. organizational and work-related stressors) facing health systems rather than focusing narrowly of their symptoms. It does so by means of a multi-centre study involving more than 60 hospitals across six European countries. By choosing a cluster randomized controlled design, this study is the first with the power to identify a causal association between implementing healthy workplace characteristics and improved staff wellbeing in European hospitals on a large scale. Two international pilot initiatives testing the transferability of Magnet key principles and accompanied by rigorous evaluations have informed the design of Magnet4Europe.[46,47] In both, the intervention hospitals improved their work environments substantially from their baselines at the end of the two-year intervention, and outcomes for staff improved. The Magnet4Europe intervention builds upon lessons learned from these pilot initiatives. The degree of innovation can also be seen in the use of a multi-faceted implementation strategy involving one-to-one twinning with international Magnet®-designated hospitals, creation of a critical mass, co-creation of solutions, development of learning collaboratives, benchmarking, and

For peer review only - http://bmjopen.bmj.com/site/about/guidelines.xhtml

interdisciplinary and management buy-in. In addition, Magnet4Europe makes a strong scientific contribution as it goes beyond traditional studies which focus solely on individual professions and disciplines separately. This allows us to study the interaction between physicians and nurses working in teams and the impact of healthy environments on their well-being and patient outcomes.

Magnet4Europe will also allow countries to learn from the experience of other health systems and understand the factors influencing their sustainability, as the study will be undertaken in six European countries representing the two dominant health system forms. The so-called Beveridge countries (England, Ireland, Sweden, Norway) are tax funded resulting in potentially more government health policy decision-making while the so-called Bismarck countries (Belgium, Germany) have an employer-based social insurance financing system, which potentially allows for more organizational variation. This study allows us to compare and contrast the impact of health care systems on the implementation and sustainability of the intervention.

The project involves dissemination and stakeholder engagement. Various stakeholders will be involved in the project including designees from international and European organizations who represent nursing professionals, health care employers, patients, and governments. Their main role is to raise awareness of the project and to support dissemination of the results as well as the Magnet4Europe consortium in formulating policy recommendations based on the scientific results.

For peer review only

For peer review only - http://bmjopen.bmj.com/site/about/guidelines.xhtml

LIST OF FIGURES

1. Figure 1 – Magnet4Europe timeline. Legend: † - Activity for group 1; ‡ - Activity for group 2; * - Activity for both groups

AUTHORS' CONTRIBUTIONS

WS, LA, MMH and LB devised the study. WS, LA, MMH and LB drafted the protocol, with assistance from JB, JB, RB, HDW, SD, JD, LEE, PG, DK, JK, RL, CM, MM, AMR, WS, DS, LSA, HS. All authors provided edits and reviewed the manuscript for important intellectual content. All authors gave final approval of the version to be published and agreed to be accountable for all aspects of this work.

FUNDING

This study is funded by the European Union's Horizon 2020 research and innovation programme under the project Magnet4Europe: Improving Mental Health and Wellbeing in the Health Care Workplace (Grant Agreement No. 848031), starting 1st January 2020 until 31st December 2023. The materials presented here are the responsibility of the authors only. The EU Commission takes no responsibility for any use made of the information set out.

COMPETING INTERESTS

None declared.

ACKNOWLEDGMENTS

We thank the American Nurses Credentialing Center (ANCC) for granting the right to use Magnet® model as core element of the Magnet4Europe intervention. The Magnet® model is a trademark of ANCC registered in the United States of America and other jurisdictions, and is being used under license from ANCC. All rights are reserved by ANCC. ANCC's consent to the use of the Intervention Hospital Magnet® mark shall not be construed as ANCC sponsoring, participating, or endorsing the

For peer review only - http://bmjopen.bmj.com/site/about/guidelines.xhtml

Magnet4Europe intervention. ©American Nurses Credentialing Center. Reproduced under license from the American Nurses Credentialing Center. All rights reserved.

MAGNET4EUROPE CONSORTIUM

The Magnet4Europe Consortium consists of Walter Sermeus (director), Luk Bruyneel, Hans De Witte, Wilmar Schaufeli, Simon Dello, Dorothea Kohnen (Belgium, Catholic University Leuven); Linda Aiken (codirector), Matthew McHugh, Mary Del Guidice, Herbert Smith, Timothy Cheney, Douglas Sloane (USA, University of Pennsylvania); Reinhard Busse, Claudia Maier, Julia Köppen, Joan Kleine (Germany, Technical University Berlin); Jonathan Drennan, Vera McCarthy, Elaine Lehane, Noeleen Brady, Anne Scott (Ireland, University College Cork); Ingeborg Strømseng Sjetne (Norway, Norwegian Institute of Public Health); Anners Lerdal, Monica Bukkøy Kjetland (Norway, Lovisenberg Diaconal Hospital); Lars E. Eriksson, Rikard Lindqvist, Lisa Smeds Alenius (Sweden, Karolinska Institutet); Jane Ball, Peter Griffiths, Jackie Bridges, Sydney Anstee (England, University of Southampton); Anne Marie Rafferty (England, King's College London); Martin McKee (England, London School of Hygiene and Tropical Medicine); Paul Van Aken, Danny Van Heusden, Kaat Siebens, Peter Van Bogaert (Belgium, University Hospital Antwerp).

For peer review only

For peer review only - http://bmjopen.bmj.com/site/about/guidelines.xhtml

REFERENCES

1 Europa-Kommisisonen. GREEN PAPER- Improving the mental health of the population Towards a strategy on mental health for the European Union. OOPEC 2005. http://ec.europa.eu/health/ph_determinants/life_style/mental/green_paper/mental_gp_en.pdf

2 Health M, The IN, Europe IN. Eu Compass for Action on Mental Health and Well-Being Mental Health in the Workplace. 2020.

3 Kim M-S, Kim T, Lee D, *et al.* Mental disorders among workers in the healthcare industry: 2014 national health insurance data. *Ann Occup Environ Med* 2018;**30**:31. doi:10.1186/s40557-018-0244-x

4 Shanafelt TD, Boone S, Tan L, *et al.* Burnout and satisfaction with work-life balance among US physicians relative to the general US population. *Arch Intern Med* 2012;**172**:1377–85. doi:10.1001/archinternmed.2012.3199

5 Aiken LH, Sloane DM, Bruyneel L, *et al.* Nurse staffing and education and hospital mortality in nine European countries: A retrospective observational study. *Lancet* 2014;**383**:1824–30. doi:10.1016/S0140-6736(13)62631-8

6 Aiken LH, Sermeus W, Van Den Heede K, *et al.* Patient safety, satisfaction, and quality of hospital care: Cross sectional surveys of nurses and patients in 12 countries in Europe and the United States. *BMJ* 2012;**344**:e1717. doi:10.1136/bmj.e1717

7 Dyrbye LN, Burke SE, Hardeman RR, *et al.* Association of clinical specialty with symptoms of burnout and career choice regret among US resident physicians. *JAMA - J Am Med Assoc* 2018;**320**:1114–30. doi:10.1001/jama.2018.12615

8 Rotenstein LS, Torre M, Ramos MA, *et al.* Prevalence of burnout among physicians a systematic review. *JAMA - J Am Med Assoc* 2018;**320**:1131–50. doi:10.1001/jama.2018.12777

For peer review only

For peer review only - http://bmjopen.bmj.com/site/about/guidelines.xhtml

9 Van Gerven E, Vander Elst T, Vandenbroeck S, *et al.* Increased risk of burnout for physicians and nurses involved in a patient safety incident. *Med Care* 2016;**54**:937–43. doi:10.1097/MLR.0000000000000582

10 Vandenbroeck S, Van Gerven E, De Witte H, *et al.* Burnout in Belgian physicians and nurses. *Occup Med (Chic Ill)* 2017;**67**:546–54. doi:10.1093/occmed/kqx126

11 Schernhammer E. Taking Their Own Lives — The High Rate of Physician Suicide. *N Engl J Med* 2005;**352**:2473–6. doi:10.1056/nejmp058014

12 Dyrbye LN, Thomas MR, Massie FS, *et al.* Burnout and suicidal ideation among U.S. medical students. *Ann Intern Med* 2008;**149**:334–41. doi:10.7326/0003-4819-149-5-200809020-00008

13 Schwenk TL, Davis L, Wimsatt LA. Depression, stigma, and suicidal ideation in medical students. *JAMA - J Am Med Assoc* 2010;**304**:1181–90. doi:10.1001/jama.2010.1300

14 Kessler RC, Barber C, Beck A, *et al.* The World Health Organization Health and Work Performance Questionnaire (HPQ). *J Occup Environ Med* 2003;**45**:156–74. doi:10.1097/01.jom.0000052967.43131.51

15 Davis MA, Cher BAY, Friese CR, *et al.* Association of US Nurse and Physician Occupation with Risk of Suicide. *JAMA Psychiatry* 2021;**78**:651–8. doi:10.1001/jamapsychiatry.2021.0154

16 Shanafelt TD, Balch CM, Bechamps G, *et al.* Burnout and medical errors among American surgeons. *Ann Surg* 2010;**251**:995–1000. doi:10.1097/SLA.0b013e3181bfdab3

17 Salyers MP, Bonfils KA, Luther L, *et al.* The Relationship Between Professional Burnout and Quality and Safety in Healthcare: A Meta-Analysis. *J Gen Intern Med* 2017;**32**:475–82. doi:10.1007/s11606-016-3886-9

18 Dewa CS, Loong D, Bonato S, *et al.* The relationship between physician burnout and quality of healthcare in terms of safety and acceptability: A systematic review. *BMJ Open*

For peer review only

2017;**7**:e015141. doi:10.1136/bmjopen-2016-015141

19 Dewa CS, Jacobs P, Thanh NX, *et al.* An estimate of the cost of burnout on early retirement and reduction in clinical hours of practicing physicians in Canada. *BMC Health Serv Res* 2014;**14**:254. doi:10.1186/1472-6963-14-254

20 Muller AE, Hafstad EV, Himmels JPW, *et al.* The mental health impact of the covid-19 pandemic on healthcare workers, and interventions to help them: A rapid systematic review. Psychiatry Res. 2020;**293**:113441. doi:10.1016/j.psychres.2020.113441

21 Vanhaecht K, Seys D, Bruyneel L, *et al.* COVID-19 is having a destructive impact on health-care workers' mental well-being. *Int J Qual Heal Care* Published Online First: 3 December 2020. doi:10.1093/intqhc/mzaa158

22 Tan BYQ, Chew NWS, Lee GKH, *et al.* Psychological Impact of the COVID-19 Pandemic on Health Care Workers in Singapore. *Ann Intern Med* 2020;**173**:317–20. doi:10.7326/M20-1083

23 Pfefferbaum B, North CS. Mental Health and the Covid-19 Pandemic. *N Engl J Med* 2020;**383**:510–2. doi:10.1056/nejmp2008017

24 Walton M, Murray E, Christian MD. Mental health care for medical staff and affiliated healthcare workers during the COVID-19 pandemic. *Eur Hear J Acute Cardiovasc Care* 2020;**9**:241–7. doi:10.1177/2048872620922795

25 European Union. European Framework for Action on Mental Health and Wellbeing. 2016. http://www.mentalhealthandwellbeing.eu/assets/docs/publications/Framework for action_19jan (1)-20160119192639.pdf

26 Panagioti M, Panagopoulou E, Bower P, *et al.* Controlled interventions to reduce burnout in physicians a systematic review and meta-analysis. JAMA Intern. Med. 2017;**177**:195–205. doi:10.1001/jamainternmed.2016.7674

For peer review only

For peer review only - http://bmjopen.bmj.com/site/about/guidelines.xhtml

27 West CP, Dyrbye LN, Erwin PJ, *et al.* Interventions to prevent and reduce physician burnout: a systematic review and meta-analysis. *Lancet* 2016;**388**:2272–81. doi:10.1016/S0140-6736(16)31279-X

28 West CP, Dyrbye LN, Shanafelt TD. Physician burnout: contributors, consequences and solutions. *J Intern Med* 2018;**283**:516–29. doi:10.1111/joim.12752

29 Kutney-Lee A, Wu ES, Sloane DM, *et al.* Changes in hospital nurse work environments and nurse job outcomes: an analysis of panel data. *Int J Nurs Stud* 2013;**50**:195–201. doi:10.1016/j.ijnurstu.2012.07.014

30 Shanafelt TD, Gorringe G, Menaker R, *et al.* Impact of organizational leadership on physician burnout and satisfaction. *Mayo Clin Proc* 2015;**90**:432–40. doi:10.1016/j.mayocp.2015.01.012

31 Diez-Pinol M, Dolan SL, Sierra V, *et al.* Personal and organizational determinants of well-being at work: The case of Swedish physicians. *Int J Health Care Qual Assur* 2008;**21**:598–610. doi:10.1108/09526860810900754

32 Hayes LJ, O'Brien-Pallas L, Duffield C, *et al.* Nurse turnover: A literature review - An update. *Int J Nurs Stud* 2012;**49**:887–905. doi:10.1016/j.ijnurstu.2011.10.001

33 Hayes LJ, O'Brien-Pallas L, Duffield C, *et al.* Nurse turnover: a literature review. *Int J Nurs Stud* 2006;**43**:237–63. doi:10.1016/j.ijnurstu.2005.02.007

34 Buerhaus PI. Current and future State of the US nursing workforce. *JAMA - J Am Med Assoc* 2008;**300**:2422–4. doi:10.1001/jama.2008.729

35 Upenieks V V. The interrelationship of organizational characteristics of magnet hospitals, nursing leadership, and nursing job satisfaction. *Health Care Manag (Frederick)* 2003;**22**:83–98. doi:10.1097/00126450-200304000-00002

36 Magnet hospitals. Attraction and retention of professional nurses. Task Force on Nursing

For peer review only - http://bmjopen.bmj.com/site/about/guidelines.xhtml

Practice in Hospitals. American Academy of Nursing. *ANA Publ* 1983;:i–xiv, 1–135.

37 Blegen MA, Goode CJ, Spetz J, *et al.* Nurse staffing effects on patient outcomes safety-net and non-safety-net hospitals. *Med Care* 2011;**49**:406–14. doi:10.1097/MLR.0b013e318202e129

38 Kelly LA, McHugh MD, Aiken LH. Nurse Outcomes in Magnet® and Non-Magnet Hospitals. *JONA J Nurs Adm* 2012;**42**:S44–9. doi:10.1097/01.NNA.0000420394.18284.4f

39 Kutney-Lee A, Stimpfel AW, Sloane DM, *et al.* Changes in patient and nurse outcomes associated with magnet hospital recognition. *Med Care* 2015;**53**:550–7. doi:10.1097/MLR.0000000000000355

40 Drenkard K. The business case for magnet®. *J Nurs Adm* 2010;**40**:263–71. doi:10.1097/NNA.0b013e3181df0fd6

41 Jayawardhana J, Welton JM, Lindrooth RC. Is there a business case for magnet hospitals? Estimates of the cost and revenue implications of becoming a magnet. *Med Care* 2014;**52**:400–6. doi:10.1097/MLR.0000000000000092

42 Stimpfel AW, Sloane DM, McHugh MD, *et al.* Hospitals Known for Nursing Excellence Associated with Better Hospital Experience for Patients. *Health Serv Res* 2016;**51**:1120–34. doi:10.1111/1475-6773.12357

43 Aiken LH, Sloane DM, Ball J, *et al.* Patient satisfaction with hospital care and nurses in England: An observational study. *BMJ Open* 2018;**8**:e019189. doi:10.1136/bmjopen-2017-019189

44 McHugh MD, Kelly LA, Smith HL, *et al.* Lower mortality in magnet hospitals. *Med Care* 2013;**51**:382–8. doi:10.1097/MLR.0b013e3182726cc5

45 Barnes H, Rearden J, Mchugh MD. Magnet® Hospital Recognition Linked to Lower Central Line-Associated Bloodstream Infection Rates. *Res Nurs Heal* 2016;**39**:96–104.

For peer review only - http://bmjopen.bmj.com/site/about/guidelines.xhtml

doi:10.1002/nur.21709

46 Aiken LH, Buchan J, Ball J, *et al.* Transformative impact of Magnet designation: England case study. *J Clin Nurs* 2008;**17**:3330–7. doi:10.1111/j.1365-2702.2008.02640.x

47 Aiken LH, Poghosyan L. Evaluation of 'magnet journey to nursing excellence program' in Russia and Armenia. *J Nurs Scholarsh* 2009;**41**:166–74. doi:10.1111/j.1547-5069.2009.01268.x

48 Aunger JA, Millar R, Rafferty AM, *et al.* How, when, and why do inter-organisational collaborations in healthcare work? A realist evaluation. *PLoS One* 2022;**17**:e0266899. doi:10.1371/JOURNAL.PONE.0266899

49 Chan A-W, Tetzlaff JM, Altman DG, *et al.* SPIRIT 2013 statement: defining standard protocol items for clinical trials. *Ann Intern Med* 2013;**158**:200–7.

50 Kim HW, Chung CH. Mitral-aortic intervalvular fibrosa pseudoaneurysm resulting in the displacement of the left main coronary artery after aortic valve replacement. *J Thorac Cardiovasc Surg* 2010;**139**. doi:10.1016/j.jtcvs.2008.07.045

51 Hemming K, Taljaard M. Sample size calculations for stepped wedge and cluster randomised trials: A unified approach. *J Clin Epidemiol* 2016;**69**:137–46. doi:10.1016/j.jclinepi.2015.08.015

52 Woertman W, De Hoop E, Moerbeek M, *et al.* Stepped wedge designs could reduce the required sample size in cluster randomized trials. *J Clin Epidemiol* 2013;**66**:752–8. doi:10.1016/j.jclinepi.2013.01.009

53 Maslach C, Jackson SE. The measurement of experienced burnout. *J Organ Behav* 1981;**2**:99–113. doi:10.1002/job.4030020205

54 Li B, Bruyneel L, Sermeus W, *et al.* Group-level impact of work environment dimensions on burnout experiences among nurses: a multivariate multilevel probit model. *Int J Nurs Stud* 2013;**50**:281–91. doi:10.1016/j.ijnurstu.2012.07.001

For peer review only - http://bmjopen.bmj.com/site/about/guidelines.xhtml

55 Cadée F, Nieuwenhuijze MJ, Lagro-Janssen ALM, *et al.* The state of the art of twinning, a concept analysis of twinning in healthcare. *Global Health* 2016;**12**:66. doi:10.1186/s12992-016-0205-5

56 Cuddy C. *Cultivating Communities of Practice: A Guide to Managing Knowledge*. Harvard Business School Press 2002. doi:10.1108/bl.2002.17015bae.001

57 Mills PD, Weeks WB. Characteristics of successful quality improvement teams: lessons from five collaborative projects in the VHA. *Jt Comm J Qual Saf* 2004;**30**:152–62. doi:10.1016/S1549-3741(04)30017-1

58 Nembhard IM. Learning and improving in quality improvement collaboratives: Which collaborative features do participants value most?: Quality and patient safety. *Health Serv Res* 2009;**44**:359–78. doi:10.1111/j.1475-6773.2008.00923.x

59 Yuan CT, Nembhard IM, Stern AF, *et al.* Blueprint for the dissemination of evidence-based practices in health care. *Issue Brief (Commonw Fund)* 2010;**86**:1–16.https://www.ncbi.nlm.nih.gov/pubmed/20469542

60 Greenhalgh T, Robert G, Macfarlane F, *et al.* Diffusion of innovations in service organizations: Systematic review and recommendations. *Milbank Q* 2004;**82**:581–629. doi:10.1111/j.0887-378X.2004.00325.x

61 Meyer AD, Goes JB. Organizational Assimilation of Innovations: A Multilevel Contextual Analysis. *Acad Manag J* 1988;**31**:897–923. doi:10.5465/256344

62 Kimberly JR, Evanisko MJ. Organizational innovation: the influence of individual, organizational, and contextual factors on hospital adoption of technological and administrative innovations. *Acad Manage J* 1981;**24**:689–713. doi:10.2307/256170

63 Richards MR, Lasater K, McHugh M. A race to the top? Competitive pressure and magnet adoption among us hospitals 1997-2012. *Med Care* 2017;**55**:384–90.

For peer review only - http://bmjopen.bmj.com/site/about/guidelines.xhtml

doi:10.1097/MLR.0000000000000650

64 Lasater KB, Richards MR, Dandapani NB, *et al.* Magnet hospital recognition in hospital systems over time. *Health Care Manage Rev* 2019;**44**:19–29. doi:10.1097/HMR.0000000000000167

65 Björklund C, Grahn A, Jensen I, *et al.* Does survey feedback enhance the psychosocial work environment and decrease sick leave? *Eur J Work Organ Psychol* 2007;**16**:76–93. doi:10.1080/13594320601112169

66 Aiken LH, Sochalski J, Lake ET. Studying outcomes of organizational change in health services. *Med Care* 1997;**35**:NS6-18. doi:10.1097/00005650-199711001-00002

67 Donabedian A. Evaluating the quality of medical care. *Milbank Q* 2005;**83**:691–729. doi:10.1111/j.1468-0009.2005.00397.x

68 Mitchell PH, Ferketich S, Jennings BM. Quality Health Outcomes Model. *J Nurs Scholarsh* 1998;**30**:43–6. doi:10.1111/j.1547-5069.1998.tb01234.x

69 Demerouti E, Nachreiner F, Bakker AB, *et al.* The job demands-resources model of burnout. *J Appl Psychol* 2001;**86**:499–512. doi:10.1037/0021-9010.86.3.499

70 Moore GF, Audrey S, Barker M, *et al.* Process evaluation of complex interventions: Medical Research Council guidance. *BMJ* 2015;**350**:h1258. doi:10.1136/bmj.h1258

71 Maslach C, Jackson SE. The measurement of experienced burnout. *J Organ Behav* 1981;**2**:99–113. doi:10.1002/job.4030020205

72 de Beer LT, Schaufeli WB, De Witte H, *et al.* Measurement invariance of the burnout assessment tool (Bat) across seven cross-national representative samples. *Int J Environ Res Public Health* 2020;**17**:1–14. doi:10.3390/ijerph17155604

73 Schaufeli WB, Desart S, De Witte H. Burnout assessment tool (Bat)—development, validity, and reliability. *Int J Environ Res Public Health* 2020;**17**:1–21. doi:10.3390/ijerph17249495

For peer review only - http://bmjopen.bmj.com/site/about/guidelines.xhtml

74 Hadžibajramović E, Schaufeli W, De Witte H. A Rasch analysis of the Burnout Assessment Tool (BAT). *PLoS One* 2020;**15**. doi:10.1371/journal.pone.0242241

75 Schaufeli WB, Shimazu A, Hakanen J, *et al.* An ultra-short measure for work engagement: The UWES-3 validation across five countries. *Eur J Psychol Assess* 2019;**35**:577–91. doi:10.1027/1015-5759/a000430

76 Wanous JP, Reichers AE, Hudy MJ. Overall job satisfaction: How good are single-item measures? *J Appl Psychol* 1997;**82**:247–52. doi:10.1037/0021-9010.82.2.247

77 Wanous JP, Hudy MJ. Single-Item Reliability: A Replication and Extension. *Organ Res Methods* 2001;**4**:361–75. doi:10.1177/109442810144003

78 Kroenke K, Spitzer R, Williams J. The Patient Health Questionnaire-2: Validity of a Two-Item Depression Screener. *Med Care* 2003;**41**:1284–92.

79 Kroenke K, Spitzer RL, Williams JBW, *et al.* Anxiety disorders in primary care: Prevalence, impairment, comorbidity, and detection. *Ann Intern Med* 2007;**146**:317–25. doi:10.7326/0003-4819-146-5-200703060-00004

80 Ware JE, GlaxoSmithKline. How to score and interpret single-item health status measures : a manual for users of the of the SF-8 health survey : (with a supplement on the SF-6 health survey). 2001;:220.http://www.worldcat.org/title/how-to-score-and-interpret-single-item-health-status-measures-a-manual-for-users-of-the-of-the-sf-8-health-survey-with-a-supplement-on-the-sf-6-health-survey/oclc/47005803

81 Buysse DJ, Reynolds CF, Monk TH, *et al.* The Pittsburgh sleep quality index: A new instrument for psychiatric practice and research. *Psychiatry Res* 1989;**28**:193–213. doi:10.1016/0165-1781(89)90047-4

82 Rahmadani VG, Schaufeli WB, Stouten J, *et al.* Engaging leadership and its implication for work engagement and job outcomes at the individual and team level: A multi-level longitudinal

For peer review only - http://bmjopen.bmj.com/site/about/guidelines.xhtml

study. *Int J Environ Res Public Health* 2020;**17**:776. doi:10.3390/ijerph17030776

83 Lake ET. Development of the practice environment scale of the nursing work index. *Res Nurs Heal* 2002;**25**:176–88. doi:10.1002/nur.10032

84 Lindström K, Elo A-L, Skogstad A, *et al. User ' S Guide for the QPS Nordic. General Nordic Questionnaire for Psychological and Social Factors at Work.* KÃ¸benhavn: : Nordic Council of Ministers 2000.

85 Aiken LH, Clarke SP, Sloane DM, *et al.* Nurses' reports on hospital care in five countries. *Health Aff* 2001;**20**:43–53. doi:10.1377/hlthaff.20.3.43

86 Notelaers G, De Witte H, Van Veldhoven M, *et al.* Construction and validation of the Short Inventory to Monitor Psychosocial Hazards. *Arb en Ergon* 2007;**44**:11–7.

87 van Loon NM, Leisink PLM, Knies E, *et al.* Red Tape: Developing and Validating a New Job-Centered Measure. *Public Adm Rev* 2016;**76**:662–73. doi:10.1111/puar.12569

88 Schaufeli WB. Applying the Job Demands-Resources model: A 'how to' guide to measuring and tackling work engagement and burnout. *Organ Dyn* 2017;**46**:120–32. doi:10.1016/j.orgdyn.2017.04.008

89 Tremblay MA, Blanchard CM, Taylor S, *et al.* Work Extrinsic and Intrinsic Motivation scale: its value for organizational psychology research. *Can J Behav Sci* 2009;**41**:213–26. doi:10.1037/a0015167

90 Bakker AB, Demerouti E. The Job Demands-Resources model: State of the art. *J Manag Psychol* 2007;**22**:309–28. doi:10.1108/02683940710733115

91 De Witte H, De Cuyper N, Handaja Y, *et al.* Associations between quantitative and qualitative job insecurity and well-being. *Int Stud Manag Organ* 2010;**40**:40–56. doi:10.2753/IMO0020-8825400103

For peer review only

For peer review only - http://bmjopen.bmj.com/site/about/guidelines.xhtml

92 Zapf D. Emotion work and psychological well-being. A review of the literature and some conceptual considerations. *Hum Resour Manag Rev* 2002;**12**:237–68. doi:10.1016/S1053-4822(02)00048-7

93 Van Veldhoven M, De Jonge J, Broersen S, *et al.* Specific relationships between psychosocial job conditions and job-related stress: A three-level analytic approach. *Work Stress* 2002;**16**:207–28. doi:10.1080/02678370210166399

94 Van den Broeck A, Vansteenkiste M, De Witte H, *et al.* Capturing autonomy, competence, and relatedness at work: Construction and initial validation of the Work-related Basic Need Satisfaction scale. *J Occup Organ Psychol* 2010;**83**:981–1002. doi:10.1348/096317909X481382

95 Schaufeli WB. Engaging leadership in the job demands-resources model. *Career Dev Int* 2015;**20**:446–63. doi:10.1108/CDI-02-2015-0025

96 Shea CM, Jacobs SR, Esserman DA, *et al.* Organizational readiness for implementing change: A psychometric assessment of a new measure. *Implement Sci* 2014;**9**. doi:10.1186/1748-5908-9-7

97 Rapley T, Girling M, Mair FS, *et al.* Improving the normalization of complex interventions: Part 1 - Development of the NoMAD instrument for assessing implementation work based on normalization process theory (NPT) 17 Psychology and Cognitive Sciences 1701 Psychology. *BMC Med Res Methodol* 2018;**18**:133. doi:10.1186/s12874-018-0590-y

98 Finch TL, Girling M, May CR, *et al.* Improving the normalization of complex interventions: Part 2 - Validation of the NoMAD instrument for assessing implementation work based on normalization process theory (NPT) 17 Psychology and Cognitive Sciences 1701 Psychology. *BMC Med Res Methodol* 2018;**18**. doi:10.1186/s12874-018-0591-x

99 Perreault K. Research Design: Qualitative, Quantitative, and Mixed Methods Approaches. *Man Ther* 2011;**16**:103. doi:10.1016/j.math.2010.09.003

For peer review only

For peer review only - http://bmjopen.bmj.com/site/about/guidelines.xhtml

100 Ltd. QIP. NVivo. 2020.https://www.qsrinternational.com/nvivo-qualitative-data-analysis-software/home

101 Bowen GA. Document analysis as a qualitative research method. *Qual Res J* 2009;**9**:27–40. doi:10.3316/QRJ0902027

102 Muller AE, Hafstad EV, Himmels JPW, *et al.* The mental health impact of the covid-19 pandemic on healthcare workers, and interventions to help them: A rapid systematic review. Psychiatry Res. 2020;**293**:113441. doi:10.1016/j.psychres.2020.113441

103 Blake H, Bermingham F, Johnson G, *et al.* Mitigating the psychological impact of covid-19 on healthcare workers: A digital learning package. *Int J Environ Res Public Health* 2020;**17**:2997. doi:10.3390/ijerph17092997

104 Sampaio F, Sequeira C, Teixeira L. Impact of COVID-19 outbreak on nurses' mental health: A prospective cohort study. *Environ Res* 2021;**194**:110620. doi:10.1016/j.envres.2020.110620

105 De Kock JH, Latham HA, Leslie SJ, *et al.* A rapid review of the impact of COVID-19 on the mental health of healthcare workers: implications for supporting psychological well-being. *BMC Public Health* 2021;**21**:1–18. doi:10.1186/s12889-020-10070-3

106 Greenberg N, Weston D, Hall C, *et al.* Mental health of staff working in intensive care during Covid-19. *Occup Med (Lond)* 2021;**71**:62–7. doi:10.1093/occmed/kqaa220

For peer review only

For peer review only - http://bmjopen.bmj.com/site/about/guidelines.xhtml

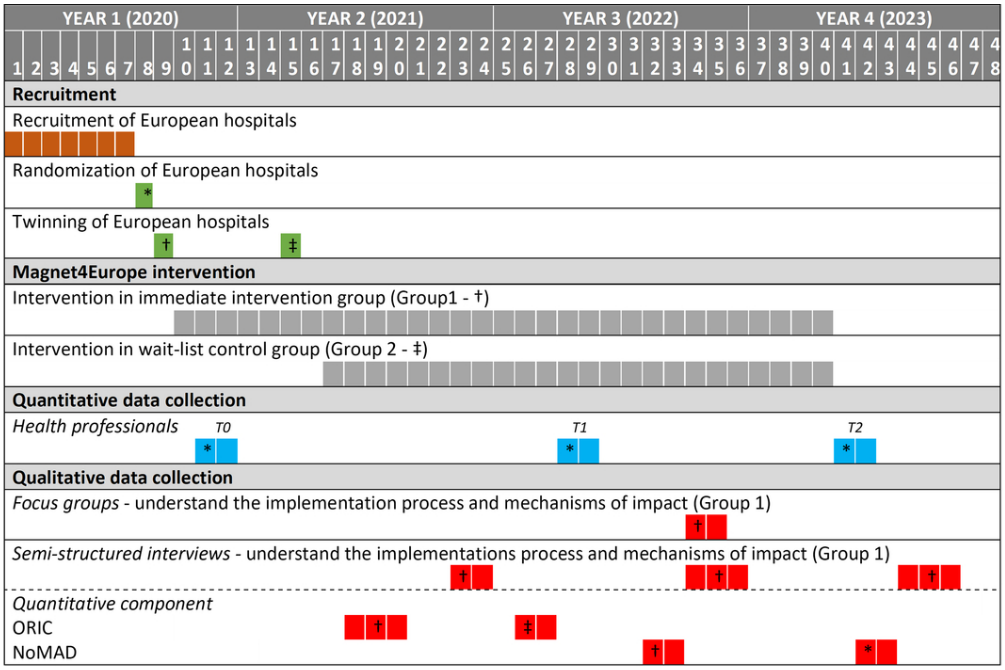

Figure 1 - Magnet4Europe timeline
† - Activity for group 1; ‡ - Activity for group 2; * - Activity for both groups

89x59mm (300 x 300 DPI)

STANDARD PROTOCOL ITEMS: RECOMMENDATIONS FOR INTERVENTIONAL TRIALS

Supplementary File 1 - SPIRIT 2013 Checklist: Recommended items to address in a clinical trial protocol and related documents*

Section/item	Item No	Description	Addressed on page number
**Administrative information**			
Title	1	Descriptive title identifying the study design, population, interventions, and, if applicable, trial acronym	1
Trial registration	2a	Trial identifier and registry name. If not yet registered, name of intended registry	2, 17
	2b	All items from the World Health Organization Trial Registration Data Set	2, 6, 7
Protocol version	3	Date and version identifier	N/A
Funding	4	Sources and types of financial, material, and other support	20
Roles and responsibilities	5a	Names, affiliations, and roles of protocol contributors	1, 20
	5b	Name and contact information for the trial sponsor	20
	5c	Role of study sponsor and funders, if any, in study design; collection, management, analysis, and interpretation of data; writing of the report; and the decision to submit the report for publication, including whether they will have ultimate authority over any of these activities	20
	5d	Composition, roles, and responsibilities of the coordinating centre, steering committee, endpoint adjudication committee, data management team, and other individuals or groups overseeing the trial, if applicable (see Item 21a for data monitoring committee)	N/A

For peer review only - http://bmjopen.bmj.com/site/about/guidelines.xhtml

Introduction

Background and rationale	6a	Description of research question and justification for undertaking the trial, including summary of relevant studies (published and unpublished) examining benefits and harms for each intervention	4-5
	6b	Explanation for choice of comparators	4-5
Objectives	7	Specific objectives or hypotheses	6
Trial design	8	Description of trial design including type of trial (eg, parallel group, crossover, factorial, single group), allocation ratio, and framework (eg, superiority, equivalence, noninferiority, exploratory)	6

Methods: Participants, interventions, and outcomes

Study setting	9	Description of study settings (eg, community clinic, academic hospital) and list of countries where data will be collected. Reference to where list of study sites can be obtained	6
Eligibility criteria	10	Inclusion and exclusion criteria for participants. If applicable, eligibility criteria for study centres and individuals who will perform the interventions (eg, surgeons, psychotherapists)	6
Interventions	11a	Interventions for each group with sufficient detail to allow replication, including how and when they will be administered	9-12
	11b	Criteria for discontinuing or modifying allocated interventions for a given trial participant (eg, drug dose change in response to harms, participant request, or improving/worsening disease)	12
	11c	Strategies to improve adherence to intervention protocols, and any procedures for monitoring adherence (eg, drug tablet return, laboratory tests)	N/A
	11d	Relevant concomitant care and interventions that are permitted or prohibited during the trial	N/A
Outcomes	12	Primary, secondary, and other outcomes, including the specific measurement variable (eg, systolic blood pressure), analysis metric (eg, change from baseline, final value, time to event), method of aggregation (eg, median, proportion), and time point for each outcome. Explanation of the clinical relevance of chosen efficacy and harm outcomes is strongly recommended	12-16
Participant timeline	13	Time schedule of enrolment, interventions (including any run-ins and washouts), assessments, and visits for participants. A schematic diagram is highly recommended (see Figure)	8

For peer review only - http://bmjopen.bmj.com/site/about/guidelines.xhtml

Sample size	14	Estimated number of participants needed to achieve study objectives and how it was determined, including clinical and statistical assumptions supporting any sample size calculations	7
Recruitment	15	Strategies for achieving adequate participant enrolment to reach target sample size	7, 12

Methods: Assignment of interventions (for controlled trials)

Allocation:

Sequence generation	16a	Method of generating the allocation sequence (eg, computer-generated random numbers), and list of any factors for stratification. To reduce predictability of a random sequence, details of any planned restriction (eg, blocking) should be provided in a separate document that is unavailable to those who enrol participants or assign interventions	7-8
Allocation concealment mechanism	16b	Mechanism of implementing the allocation sequence (eg, central telephone; sequentially numbered, opaque, sealed envelopes), describing any steps to conceal the sequence until interventions are assigned	7-8
Implementation	16c	Who will generate the allocation sequence, who will enrol participants, and who will assign participants to interventions	7-8
Blinding (masking)	17a	Who will be blinded after assignment to interventions (eg, trial participants, care providers, outcome assessors, data analysts), and how	N/A
	17b	If blinded, circumstances under which unblinding is permissible, and procedure for revealing a participant's allocated intervention during the trial	N/A

Methods: Data collection, management, and analysis

Data collection methods	18a	Plans for assessment and collection of outcome, baseline, and other trial data, including any related processes to promote data quality (eg, duplicate measurements, training of assessors) and a description of study instruments (eg, questionnaires, laboratory tests) along with their reliability and validity, if known. Reference to where data collection forms can be found, if not in the protocol	8, 11-16
	18b	Plans to promote participant retention and complete follow-up, including list of any outcome data to be collected for participants who discontinue or deviate from intervention protocols	12

Data management	19	Plans for data entry, coding, security, and storage, including any related processes to promote data quality (eg, double data entry; range checks for data values). Reference to where details of data management procedures can be found, if not in the protocol	17
Statistical methods	20a	Statistical methods for analysing primary and secondary outcomes. Reference to where other details of the statistical analysis plan can be found, if not in the protocol	14-16
	20b	Methods for any additional analyses (eg, subgroup and adjusted analyses)	14-16
	20c	Definition of analysis population relating to protocol non-adherence (eg, as randomised analysis), and any statistical methods to handle missing data (eg, multiple imputation)	14
**Methods: Monitoring**			
Data monitoring	21a	Composition of data monitoring committee (DMC); summary of its role and reporting structure; statement of whether it is independent from the sponsor and competing interests; and reference to where further details about its charter can be found, if not in the protocol. Alternatively, an explanation of why a DMC is not needed	N/A
	21b	Description of any interim analyses and stopping guidelines, including who will have access to these interim results and make the final decision to terminate the trial	N/A
Harms	22	Plans for collecting, assessing, reporting, and managing solicited and spontaneously reported adverse events and other unintended effects of trial interventions or trial conduct	N/A
Auditing	23	Frequency and procedures for auditing trial conduct, if any, and whether the process will be independent from investigators and the sponsor	N/A
**Ethics and dissemination**			
Research ethics approval	24	Plans for seeking research ethics committee/institutional review board (REC/IRB) approval	2, 17
Protocol amendments	25	Plans for communicating important protocol modifications (eg, changes to eligibility criteria, outcomes, analyses) to relevant parties (eg, investigators, REC/IRBs, trial participants, trial registries, journals, regulators)	17

For peer review only - http://bmjopen.bmj.com/site/about/guidelines.xhtml

Consent or assent	26a	Who will obtain informed consent or assent from potential trial participants or authorised surrogates, and how (see Item 32)	12
	26b	Additional consent provisions for collection and use of participant data and biological specimens in ancillary studies, if applicable	N/A
Confidentiality	27	How personal information about potential and enrolled participants will be collected, shared, and maintained in order to protect confidentiality before, during, and after the trial	suppl. material #2
Declaration of interests	28	Financial and other competing interests for principal investigators for the overall trial and each study site	20
Access to data	29	Statement of who will have access to the final trial dataset, and disclosure of contractual agreements that limit such access for investigators	17
Ancillary and post-trial care	30	Provisions, if any, for ancillary and post-trial care, and for compensation to those who suffer harm from trial participation	N/A
Dissemination policy	31a	Plans for investigators and sponsor to communicate trial results to participants, healthcare professionals, the public, and other relevant groups (eg, via publication, reporting in results databases, or other data sharing arrangements), including any publication restrictions	17
	31b	Authorship eligibility guidelines and any intended use of professional writers	17
	31c	Plans, if any, for granting public access to the full protocol, participant-level dataset, and statistical code	17
**Appendices**			
Informed consent materials	32	Model consent form and other related documentation given to participants and authorised surrogates	12, suppl. material #2
Biological specimens	33	Plans for collection, laboratory evaluation, and storage of biological specimens for genetic or molecular analysis in the current trial and for future use in ancillary studies, if applicable	N/A

*It is strongly recommended that this checklist be read in conjunction with the SPIRIT 2013 Explanation & Elaboration for important clarification on the items. Amendments to the protocol should be tracked and dated. The SPIRIT checklist is copyrighted by the SPIRIT Group under the Creative Commons "Attribution-NonCommercial-NoDerivs 3.0 Unported" license.

For peer review only - http://bmjopen.bmj.com/site/about/guidelines.xhtml

Supplementary File 2 – Participant information sheet and consent form for quantitative (p. 2-3) and qualitative data collection (p.4-5) (English version – Example)

For peer review only

For peer review only - http://bmjopen.bmj.com/site/about/guidelines.xhtml

Magnet4Europe - Improving Mental Health and Wellbeing in the Health Care Workplace

Dear colleague,

you have been invited to take part in a research study that aims to improve mental health and wellbeing among health professionals in Europe. The project is funded under the European Union's Horizon 2020 Research and Innovation programme from 2020 to 2023 (Grant Agreement 848031). The study will be conducted in compliance with your country's ethics guidelines for scientific studies on individuals. Your participation will neither involve costs nor reimbursements. You can withdraw from the study at any time, without giving a reason. Before you decide whether to take part it is important for you to understand why the research is being done and what it will involve. Please take your time to read the following information carefully and to decide whether you wish to take part or not. Ask us if there is anything that is not clear or if you would like to receive more information. For questions, please reach out to the Magnet4Europe project coordinator prof. dr. Walter Sermeus at walter.sermeus@kuleuven.be.

What is the purpose of the study?

Mental health and wellbeing are among the highest priorities of the public health agenda in the European Union (EU). A large-scale European study of hospital work conditions and associated nurse and patient outcomes revealed high rates of job dissatisfaction and burnout, with burnout rates varying between 10% and 78%. Physician burnout rates ranging from 25% to 60% have been reported, varying across organizations and medical specialties. Magnet4Europe aims to redesign clinical environments in sixty hospitals in five European countries (Belgium, United Kingdom, Germany, Ireland, and Sweden) to promote the mental health and wellbeing of health professionals. Magnet4Europe will implement an evidence-based intervention based on the successful Magnet Recognition Program®, a voluntary hospital designation for nursing care excellence by the American Nurses Credentialing Center. Countless studies have shown that Magnet-recognized hospitals have lower health professional burnout and safer patient care suggesting that the Magnet journey is an intervention that results in positive changes Magnet4Europe seeks to achieve.

How does this research study work?

This research study will be carried out between January 2020 and December 2023 in adult inpatient units. The intervention itself will officially start in September 2020 in participating European hospitals and will target the five overarching principles of the Magnet® concept: structural empowerment of clinical staff, transformational leadership, exemplary and evidence-based professional practice, new knowledge, innovations, and improvements, and empirical outcomes. These principles will serve as a continuous feedback loop as to whether organizational changes are producing the intended outcomes for staff and patients. The Magnet® blueprint provides definitions of the principles and gives examples of evidence-based indicators that reflect progress toward achieving them. To facilitate the implementation process, each participating European hospital receives support by one to one twinning with an experienced Magnet recognized hospital including annual on-site visits and by the creation of a critical mass of participating hospitals drawing public interest and promoting innovation, and replication. Before the intervention period officially starts, eligible participants will receive a link to the online platform Meplis Care Monitor via mail in May/June 2020. Following the link, they will be able to register first and then to complete a questionnaire. Within the scope of this questionnaire participants will be asked about various aspects of their work environment (work relations, job satisfaction, perception of the quality of care and workload) and mental health. In the course of the study, this procedure will be repeated annually for a total of 4 measurement occasions. In addition to the surveys, occasional focus groups (i.e., moderated interactive group discussions) will take place in a selection of hospitals between July 2021 and September 2023. The focus groups aim to understand clinicians' personal views and thoughts on the process of the intervention and to identify barriers and facilitators to implementation of the intervention.

Why have I been invited and am I eligible?

60 hospitals across the five European countries being involved have agreed to participate in this study. Within each participating hospital, all adult inpatient units including ICU and ER have been selected. You are eligible to participate if you (1) have direct patient contact and (2) meet the minimum standards of Directive 2013/55/EU amending Directive 2005/35/EC on the recognition of professional qualifications. You are invited to participate in this study, because you belong to the clinical staff of one of these departments and meet the selection criteria.

What will my participation in the study involve?

In order to participate in the survey, we ask you to register on Meplis Care Monitor after receiving your activation link. Following the instructions given in the questionnaire, we ask you to answer a number of questions about your perception of your working environment: the relationship with the doctors, the workload and staffing of the ward, any intention (if any) to leave the profession, patient safety, etc. The questionnaire takes approx. 20 minutes to complete; it should be completed within two weeks of receiving this invitation. Once you have successfully created an account and participated in the first survey, you will receive an automatically generated mail by Meplis Care Monitor for three more times, inviting you to participate in the annual survey. If you are new to this hospital and did not participate in the last measurement occasion(s) please reach out to the person instructed by the hospital in order to invite you to the Meplis Care Monitor platform.

Do I have to take part?

It is up to you to decide whether or not to take part. If you do decide to take part you will be given this information sheet to keep. After signing up at Meplis Care Monitor, you will be asked to express your consent before completing the questionnaire. You will not be able to engage in the survey prior to providing an informed consent. If you decide to take part you are still free to withdraw at any time and without giving a reason. The choice that you make will have no bearing on your job or on any work-related evaluations or reports. You may change your mind later and stop participating even if you agreed earlier.

Are there any benefits for me in joining the study?

You have no direct benefit from participation and will not be compensated for your participation (there are no costs or compensation associated with participation). However, we hope that your participation in the study will help us to gain more insight on how you perceive hospital care and the demands that are placed on clinical staff. By doing so, you can help to improve the working environment and safety of patient care.

Are there any risks for me in joining the study?

There are no disadvantages associated with participation. Neither the research team nor the hospital will know whether you have participated in the study or not. A possible refusal to participate in the study will have no adverse effect on your employment at this hospital. Nor will your answers have any effect on your participation. However, you may feel uncomfortable reading the information and answering the questions.

Who has approved this study?

The research project was submitted to the central and/or local ethics committees within your country for approval. This authority has not raised any objections to the design of the study.

Will information about me be kept confidentially?

If you agree to participate in this study, a unique code will be stored along with your answers. Nobody but you can make the connection between your identity, this code, and your answers. Your data will be processed in accordance with Regulation (EU) 2016/679 of 27 April 2016 on the protection of individuals with regard to the processing of personal data and on the free movement of such data, which entered into force on 25 May 2018. If the results of the study are published, your anonymity is guaranteed. The final results of the analyses will be published on the Magnet4Europe website (www.magnet4europe.eu) and will be publicly accessible. Your identity will not be revealed in any of these cases. Individual answers will not be published and also research reports will not include information on clinical staff or individual hospitals. Neither the hospital nor your superiors will have access to individual answers.

For peer review only - http://bmjopen.bmj.com/site/about/guidelines.xhtml

Consent to participate in the study

	Mark 'Yes' to continue
I hereby declare that I have been informed in an understandable manner about the nature, method and purpose of this study.	Yes
I agree to participate in this scientific research.	Yes
I am aware that participation in these studies does not entail any additional costs and that there is no financial benefit.	Yes
I am aware that I can withdraw at any time up to the moment that the data is kept in the database without having to make a statement and without this in any way affecting me.	Yes
I understand that my data is collected and registered confidentially, and that the principal investigator guarantees its confidentiality.	Yes

Date	Signature

For peer review only

Magnet4Europe - Improving Mental Health and Wellbeing in the Health Care Workplace

Dear Sir/Madam,

you have been invited to take part in a research study that aims to improve mental health and wellbeing among health professionals in Europe. The project is funded under the European Union's Horizon 2020 Research and Innovation programme from 2020 to 2023 (Grant Agreement 848031). The study will be conducted in compliance with your country's ethics guidelines for scientific studies on individuals. Your participation will neither involve costs nor reimbursements. You can withdraw from the study at any time, without giving a reason. Before you decide whether to take part it is important for you to understand why the research is being done and what it will involve. Please take your time to read the following information carefully and to decide whether you wish to take part or not. Ask us if there is anything that is not clear or if you would like to receive more information. For questions, please reach out to the Magnet4Europe project coordinator prof. dr. Walter Sermeus at walter.sermeus@kuleuven.be

What is the purpose of the study?

Mental health and wellbeing are among the highest priorities of the public health agenda in the European Union (EU). A large-scale European study of hospital work conditions and associated nurse and patient outcomes revealed high rates of job dissatisfaction and burnout, with burnout rates varying between 10% and 78%. Physician burnout rates ranging from 25% to 60% have been reported, varying across organizations and medical specialties. Magnet4Europe aims to redesign clinical environments in sixty hospitals in six European countries (Belgium, United Kingdom, Germany, Ireland, Norway, and Sweden) to promote the mental health and wellbeing of health professionals. Magnet4Europe will implement an evidence-based intervention based on the successful Magnet Recognition Program®, a voluntary hospital designation for nursing care excellence by the American Nurses Credentialing Center. Countless studies have shown that Magnet-recognized hospitals have lower health professional burnout and safer patient care suggesting that the Magnet journey is an intervention that results in positive changes Magnet4Europe seeks to achieve.

How does this research study work?

This research study will be carried out between January 2020 and December 2023 in adult inpatient units. The intervention itself will officially start in September 2020 in participating European hospitals and will target the five overarching principles of the Magnet® concept: structural empowerment of clinical staff, transformational leadership, exemplary and evidence-based professional practice, new knowledge, innovations, and improvements, and empirical outcomes. These principles will serve as a continuous feedback loop as to whether organizational changes are producing the intended outcomes for staff and patients. The Magnet® blueprint provides definitions of the principles and gives examples of evidence-based indicators that reflect progress toward achieving them. To facilitate the implementation process, each participating European hospital receives support by one to one twinning with an experienced Magnet recognized hospital including annual on-site visits and by the creation of a critical mass of participating hospitals drawing public interest and promoting innovation, and replication.

You are asked to participate in this study because your workplace belongs to one of the hospitals participating in the Magnet4Europe project. A selection of nurses and doctors working at the participating hospitals will be asked to participate in focus groups (i.e moderated interactive group discussions) or individual interviews. Focus groups and interviews will be used to investigate experiences and perceptions of the implementation process and to identify factors that hinder or facilitate the implementation of the intervention.

Why have I been invited and am I eligible?

60 hospitals across the six European countries being involved have agreed to participate in this study. Within each participating hospital, all adult inpatient units including ICU and ER have been selected. You are eligible to participate if you (1) have direct patient contact and (2) meet the minimum standards of Directive 2013/55/EU amending Directive 2005/35/EC on the recognition of professional qualifications. You are invited to participate in this study, because you belong to the clinical staff of one of these departments and meet the selection criteria.

What will my participation in the study involve?

We want to investigate perceptions of the clinical work environment and to monitor the mental health and well-being of healthcare professionals among the hospitals participating in Magnet4Europe. Participation means that you will participate in a focus group discussion (i.e together with about 2-7 other people from your hospital) or in an individual interview that will be about your experiences and experiences of the Magnet4Europe project at your workplace. The interviews and the focus group discussions are estimated to take about 30–60 minutes and 90–120 minutes respectively. The interviews and focus group discussions are conducted in person or via virtual means. The interviews and focus group discussions will be recorded through audio recording so that they can later be transcribed verbatim and analyzed.

Do I have to take part?

Your participation is voluntary and you can choose to cancel the participation at any time. If you choose not to participate or want to cancel your participation, you do not need to state why, nor will it affect your work or employment in the future. If you wish to cancel your participation, you should contact the person responsible for the study (see contact details at the end of the information letter).

Are there any benefits or risks for me to participate in the study?

You have no direct benefit from participation and will not be compensated for your participation (there are no costs or compensation associated with participation). However, we hope that your participation in the study will help us to gain more insight on how you perceive the implementation process in your hospital. By doing so, you can help to improve the working environment and safety of patient care. There are no disadvantages associated with participation. You should be aware, however, that the questions discussed during the interviews and focus groups can elicite discomfort or other emotional responses. If you feel that you need to talk to someone after the interviews, we recommend that you contact occupational health care expert at your hospital.

Will information about me be kept confidentially?

The research being done in the community may draw attention and if you participate you may be asked questions by other people in the community. We will not be sharing any information about you to anyone outside of the research team. If you participate in a focus group discussion, we will ask you and others in the group not to talk to people outside the group about what was said in the group. We will, in other words, ask each of you to keep what was said in the group confidential. You should know, however, that we cannot stop or prevent participants who were in the group from sharing things that should be confidential.

Who has approved this study?

The research project was submitted to the central and/or local ethics committees within your country for approval. This authority has not raised any objections to the design of the study.

How do I get information about the results of the study?

The results of the study will be compiled at the group level and reported in such a way that no individual individuals will be able to be identified. The results of the study will be published in scientific journals and research reports in such a way that individual answers, individuals, clinics or hospitals cannot be identified. Results from the study will also be published on Magnet4Europe's website, www.magnet4europe.eu, but will not contain information that allows individuals or hospitals to be identified.

Feel free to contact us if you have any questions or if you would like more information.

For peer review only - http://bmjopen.bmj.com/site/about/guidelines.xhtml

Consent to participate in the study

I have been given oral and written information about the study and have had the opportunity to ask questions. I may retain the written information.

☐ I agree to participate in an interview or focus group discussion within the study Magnet4Europe

☐ I agree that information about me is processed in the manner described in the research information.

Date	Signature

For peer review only

BMJ Open

A workplace organizational intervention to improve hospital nurses' and physicians' mental health: study protocol for the Magnet4Europe wait-list cluster randomized controlled trial

Journal:	*BMJ Open*
Manuscript ID	bmjopen-2021-059159.R2
Article Type:	Protocol
Date Submitted by the Author:	13-Jul-2022
Complete List of Authors:	Sermeus, Walter; Katholieke Universiteit Leuven, Department of Public Health and Primary Care<br>Aiken, Linda; University of Pennsylvania, Center for Health Outcomes and Policy Research<br>Ball, Jane; University of Southampton, Faculty of Health Sciences<br>Bridges, Jackie; University of Southampton, Faculty of Health Sciences<br>Bruyneel, Luk; Katholieke Universiteit Leuven, Department of Public Health and Primary Care<br>Busse, Reinhard; Technical University of Berlin, Health Care Management<br>De Witte, Hans; Katholieke Universiteit Leuven, Occupational & Organisational Psychology and Professional Learning<br>Dello, Simon; Katholieke Universiteit Leuven, Department of Public Health and Primary Care<br>Drennan, Jonathan; University College Cork National University of Ireland<br>Eriksson, Lars E; Karolinska Institutet, Department of Neurobiology, Care Sciences and Society, Karolinska Institutet<br>Griffiths, Peter; University of Southampton, Faculty of Health Sciences<br>Kohnen, Dorothea; KU Leuven Faculty of Psychology and Educational Sciences, Work, Organisational, and Personnel Psychology<br>Köppen, Julia; Technical University of Berlin, Department of Healthcare Management<br>Lindqvist, Rikard; Karolinska Institutet, Department of Neurobiology, Care Sciences and Society, Karolinska Institutet<br>Maier, Claudia; Technische Universitat Berlin, Department of Healthcare Management<br>McHugh, Matthew; University of Pennsylvania, School of Nursing, Center for Health Outcomes and Policy Research<br>McKee, Martin; London School of Hygiene & Tropical Medicine<br>Rafferty, Anne Marie; King's College London, Florence Nightingale School of Nursing and Midwifery<br>Schaufeli, Wilmar; Katholieke Universiteit Leuven; Utrecht University, Department of Psychology<br>Sloane, Douglas; University of Pennsylvania<br>Alenius, Lisa Smeds; Karolinska Institutet, Department of Learning, Informatics, Management and Ethics, Karolinska Institutet<br>Smith, Herbert; University of Pennsylvania,<br>Magnet4Europe consortium, Magnet4Europe consortium; Magnet4Europe

	consortium
**Primary Subject Heading**:	Health policy
Secondary Subject Heading:	Health policy, Health services research, Mental health, Public health, Nursing
Keywords:	Health policy < HEALTH SERVICES ADMINISTRATION & MANAGEMENT, Protocols & guidelines < HEALTH SERVICES ADMINISTRATION & MANAGEMENT, MENTAL HEALTH, Human resource management < HEALTH SERVICES ADMINISTRATION & MANAGEMENT, Organisation of health services < HEALTH SERVICES ADMINISTRATION & MANAGEMENT

SCHOLARONE™ Manuscripts

A WORKPLACE ORGANIZATIONAL INTERVENTION TO IMPROVE HOSPITAL NURSES' AND PHYSICIANS' MENTAL HEALTH: STUDY PROTOCOL FOR THE MAGNET4EUROPE WAIT-LIST CLUSTER RANDOMIZED CONTROLLED TRIAL

Authors

Sermeus Walter, KU Leuven, Belgium (first author)

Aiken Linda, University of Pennsylvania, USA (first author)

Ball Jane, University of Southampton, UK

Bridges Jackie, University of Southampton, UK

Bruyneel Luk, KU Leuven, Belgium

Busse Reinhard, Technical University Berlin, Germany

De Witte Hans, KU Leuven, Belgium

Dello Simon, KU Leuven, Belgium

Drennan Jonathan, University College Cork, Ireland

Eriksson Lars E., Karolinska Institutet, Sweden

Griffiths Peter, University of Southampton, UK

Kohnen, Dorothea, KU Leuven, Belgium

Köppen Julia, Technical University Berlin, Germany

Lindqvist Rikard, Karolinska Institutet, Sweden

Maier Claudia, Technical University Berlin, Germany

McHugh Matthew, University of Pennsylvania, USA

McKee Martin, London School of Hygiene and Tropical Medicine, UK

Rafferty Anne Marie, King's College London, UK

Schaufeli Wilmar B., KU Leuven, Belgium

Sloane Douglas, University of Pennsylvania, USA

Smeds Alenius Lisa, Karolinska Institutet, Sweden

Smith Herbert, University of Pennsylvania, USA

For the Magnet4Europe consortium

Corresponding author: Walter Sermeus, Leuven Institute for Healthcare Policy, KU Leuven, Kapucijnenvoer 35, 3000 Leuven, Belgium; e-mail: walter.sermeus@kuleuven.be

WORD COUNT

Words: 5395

For peer review only - http://bmjopen.bmj.com/site/about/guidelines.xhtml

ABSTRACT

Introduction The increasing burden of mental distress reported by healthcare professionals is a matter of serious concern and there is a growing recognition of the role of the workplace in creating this problem. Magnet® hospitals, a model shown to attract and retain staff in US research, creates positive work environments that aim to support the well-being of healthcare professionals.

Methods and analysis Magnet4Europe is a cluster randomized controlled trial, with wait list controls, designed to evaluate the effects of organizational redesign, based on the Magnet® model, on nurses' and physicians' well-being in general acute care hospitals, using a multicomponent implementation strategy. The study will be conducted in more than 60 general acute care hospitals in Belgium, England, Germany, Ireland, Norway, and Sweden. The primary outcome is burnout among nurses and physicians, assessed in longitudinal surveys of nurses and physicians at participating hospitals. Additional data will be collected from them on perceived work environments, patient safety, and patient quality of care and will be triangulated with data from medical records, including case mix adjusted in-hospital mortality. The process of implementation will be evaluated using qualitative data from focus group and key informant interviews.

Ethics and dissemination This study was approved by the Ethics Committee Research UZ/KU Leuven, Belgium; additionally, ethics approval is obtained in all other participating countries either through a central or decentral authority. Findings will be disseminated at conferences, through peer reviewed manuscripts, and via social media.

Trial registration number ISRCTN10196901.

For peer review only - http://bmjopen.bmj.com/site/about/guidelines.xhtml

STRENGTHS AND LIMITATIONS OF THIS STUDY

- Magnet4Europe uses a cluster randomized controlled trial design with wait-list controls in over 60 hospitals in six European countries.
- A multicomponent strategy is used to support implementation of a complex intervention at the hospital level, aimed to improve clinician well-being.
- The Magnet4Europe intervention is tailored to European hospitals, facilitating implementation and transferability without disrupting clinical practice.
- Contextual factors such as different health systems within and across countries may impact the standardized Magnet4Europe intervention and unintentionally lead to variation in the intervention and outcomes.
- Embedding a complex intervention at hospital-level takes time and any effect might become apparent only after the study ends.

For peer review only

INTRODUCTION

Mental health and well-being are high priorities for the European Union (EU).[1] Mental health conditions account for 22% of the EU's disease burden measured in Years Lived with Disability, thus imposing a significant burden on individuals, society and the economy.[2] Health workers experience greater levels of job-related burnout and other mental health disorders than those in other sectors.[3,4] An earlier European study of working conditions in European hospitals, identified high rates of job dissatisfaction and burnout, with burnout rates among nurses varying from 10% to 78%[5,6] while the corresponding figures from studies of physicians ranged from 25% to 60%, varying among organizations and medical specialties.[7–10] Burnout in the healthcare workforce not only impacts on those experiencing it, leading to depression, substance abuse and even suicide,[11–15] but is also associated with worse patient outcomes, lower patient satisfaction,[6] medical errors,[16] reduced quality and safety[17] and reduced efficiency of hospitals.[18] The resulting lost productivity, combined with current recruitment challenges, further threatens the already overstretched health workforce, widening the gap between provision of health services and population needs.[19]

This situation has been exacerbated by the COVID-19 pandemic,[20] increasing anxiety and stress among clinicians[21] and further impacting on health workers' mental health.[22–24]

Effective, affordable and sustainable interventions to improve health professionals' mental health and well-being are essential if we are to interrupt the vicious cycle of high burnout, worsening mental health, lost productivity, and unsafe care.[25] Interventions to prevent burnout can be divided into two types: firstly, psychological interventions aimed at individual level and secondly workplace redesign. There is only limited evidence of the impact of individual psychological approaches to improve resilience and coping skills (e.g. mindfulness).[26] The second approach, redesigning the organizational environment in which health professionals provide care, aims to reduce modifiable sources of clinician stress and burnout, reinforce clinicians' perceptions of well-being, and enhance their autonomy and control over the conditions of their work. There is substantial research pointing to organizational redesign as the most promising of these two approaches.[27]

For peer review only - http://bmjopen.bmj.com/site/about/guidelines.xhtml

The finding that the causes underlying burnout in nurses and physicians are similar and, especially, are rooted within the work environment[28–31] provides a compelling rationale for prioritizing the redesign of hospital environments to address this issue. In the 1980s, hospitals in the US were faced with high nurse turnover,[32,33] low retention, and increasing early retirement rates.[34] Despite the general nursing shortage, some US hospitals - later referred to as Magnet® hospitals - were more successful in recruiting and retaining nurses by creating a positive work environment. Features characterizing these hospitals included flat decentralized organizational structures, empowering frontline staff in decision making and transformational leadership.[35] The original characteristics of these hospitals[36] were developed into fourteen forces of Magnetism and later configured into five components of the Magnet® model that was used to establish the criteria for Magnet® designation.[37] A robust body of evidence, primarily stemming from US hospitals, documents the association of the Magnet® model with improved well-being of staff including lower burnout, higher job satisfaction, lower intent to leave their job,[38,39] in addition to positive financial outcomes for organizations,[40,41] higher levels of patient satisfaction,[42,43] and improved clinical outcomes.[44,45] Longitudinal panel studies have found that hospitals that follow the Magnet® model improve their work environments, well-being of staff and patient outcomes, compared to other hospitals. Two international pilots tested the transferability of the Magnet® model, demonstrating the feasibility of achieving positive results outside the USA. One, in England, focused primarily on the implementation of the Magnet® process.[46] The other, a four-hospital intervention with accompanying evaluation in Russia and Armenia, used the Magnet® model with twinning with Magnet® recognized hospitals.[47] In both cases, the intervention hospitals improved their work environments substantially from their baselines within the two-year intervention and achieved better outcomes for staff, such as reduced dissatisfaction, intent to leave, and emotional exhaustion, while perceived quality of care improved. Despite the large body of evidence and recent uptake in two European hospitals, Magnet® organizational redesign initiatives have not been adopted widely in Europe.

For peer review only - http://bmjopen.bmj.com/site/about/guidelines.xhtml

The aim of the Magnet4Europe project is to evaluate the ability of organizational redesign in European hospitals to improve nurses' and physicians' mental health and well-being. The organizational redesign intended in Magnet4Europe is a system level intervention, targeting the hospital as a whole on the level of the organization. Through co-creation and stakeholder co-designed adaptations, the Magnet® model is adapted to the European context, providing the first element and basis for the Magnet4Europe intervention. In addition to this first component, one-to-one twinning with Magnet® recognized hospitals, learning collaboratives and a critical mass of hospitals are also introduced into the intervention. While the Magnet4Europe intervention shares common elements with other organizational interventions, the combination of all the above components, targeting two major clinical professions – both nurses and physicians – and its primary focus on the work environment as levers for change differentiate the Magnet4Europe intervention from any other organizational intervention and make it unique in its kind.[48]

METHODS AND ANALYSIS

Study design and setting

Magnet4Europe will use a usual-practice cluster randomized controlled trial with wait-list controls in a nested mixed-methods evaluation. The study protocol for this study follows "the Standard protocol items: recommendations for intervention trials 2013 statement" (SPIRIT; supplementary file 1).[49] The setting will be acute general hospitals in six European countries, including both European Union member states (Belgium, Germany, Ireland and Sweden) and non-European member states (England and Norway). These six countries also represent two dominant types of health systems, Bismarck and Beveridge,[50] that vary with regard to the extent of national government intervention in health services delivery, something that may be a factor in uptake of innovation.

Eligibility

European hospitals are eligible to participate if: (1) no Magnet® designation by the American Nursing Credentialing Centre (ANCC) had been acquired in the past or at the time of the start of the intervention, (2) bed size ≥150, and (3) the hospital is focused on acute care for adults, including wards

For peer review only - http://bmjopen.bmj.com/site/about/guidelines.xhtml

in the area of internal medicine and/or surgery. Excluded are highly specialized hospitals, e.g., psychiatric hospitals, tropical medicine, or paediatrics.

Within each hospital, registered nurses, and physicians (including residents) will be eligible to participate in the quantitative and qualitative evaluation of the intervention if they: (1) have direct patient contact, (2) meet the minimum qualifications as stipulated by Directive 2013/55/EU amending Directive 2005/36/EC on the recognition of professional qualifications, and (3) work on adult inpatient units including intensive care units (ICU) and the emergency room (ER). Nurses and physicians working in specialized units such as neonatology, paediatrics, obstetrics, psychiatry, operating room, pathology, microbiology, radiology, and medical imaging will be excluded.

Study sample

A two-phased non-probability strategy will be used to sample organizations at the hospital level. First, in February and March 2019, principal investigators in the six European countries contacted eligible hospitals (see below) within those countries to consider taking part in the study using various recruitment strategies. Hospitals were informed of the Magnet4Europe objectives and methodology and were invited to show their interest in participating by submitting a letter of intent by March 2019. In spring 2020, participating hospitals (some of the sites and other new ones), were asked to confirm their commitment by signing an agreement committing to participate for the full duration of the study period and adhere to the Magnet4Europe protocol.

Sample size will be determined based on the power calculation for a cluster randomized controlled trial with before-and-after measures (CRT-BA).[51] The calculated design effect is based on average cluster size and the intraclass correlation coefficient (ICC), a measure of the amount of dependency among observations within classes (i.e. hospitals in our study).[51,52] Power calculation will be performed for the primary outcome of interest, burnout, measured by the emotional exhaustion subscale of the Maslach Burnout Inventory [53]. The estimated prevalence rate of emotional exhaustion is 30% based upon the team's earlier RN4CAST study.[6] In order to detect a 20% relative

For peer review only - http://bmjopen.bmj.com/site/about/guidelines.xhtml

reduction (i.e. a 6 percentage point reduction) in burnout with a power of 80% and an alpha of 5%, an average cluster size of 200 and an ICC of 0.025,[54] this study will require 10,253 health professionals in 51 clusters per measurement occasion (at endpoint of the study).

Based on the power calculation, we aim to include 60 general acute care hospitals in six countries (Belgium, England, Germany, Ireland, Norway, and Sweden) and recruit at least 200 professionals per hospital.

Randomization

Randomization to intervention and control group is at the hospital level. Within each country hospitals will be matched on three characteristics, (1) bed size, (2) teaching status and (3) high technology, using a Euclidian distance matrix based on the smallest within pair covariate distance. Subsequently, simple random sampling - allowing for selection with equal probability - will be used to allocate one hospital within each matched pair of hospitals to the immediate intervention group or the wait list control group. Both immediate intervention group and wait list controls will be informed which group they have been allocated to. Given the characteristics of the intervention in which active participation of the hospitals is required, blinding to the intervention will not be possible.

Intervention

A multicomponent intervention comprising organizational redesign of hospitals will be implemented in both arms of the study. The intervention was launched in the immediate intervention group in month 10 of the project (October 2020) and will have a total duration of 31 months. The intervention in the usual-practice wait-list control group will be initiated in month 17 (May 2021) and will last for 24 months (Figure 1).

The intervention consists of five distinct components: The Magnet blueprint, one-to-one twinning between European hospitals and US Magnet designated hospitals, learning collaboratives involving group meetings to share best practices, critical mass, and communication and feedback.

For peer review only

For peer review only - http://bmjopen.bmj.com/site/about/guidelines.xhtml

The first part of the intervention comprises the elements outlined in the Magnet® manual of organizational redesign. This contains a step-by-step blueprint through the five overarching Magnet® components: (1) structural empowerment of clinical staff; (2) transformational leadership; (3) exemplary and evidence-based professional practice; (4) new knowledge, innovations, and improvements; and (5) empirical outcomes. These principles serve as continuous feedback loops as to whether organizational changes are producing the intended outcomes for health professionals and patients. The manual provides definitions of the principles and gives examples of evidence-based indicators that reflect progress toward achieving them. Using the Magnet4Europe Gap Analysis Tool (adapted from the ANCC Magnet® Gap Analysis tool), each participating European hospital will, in collaboration with their Magnet® twinning partner, perform a gap analysis that will illuminate the gap between the as-is situation and the aspirational organizational features as delineated in the Magnet® blueprint. The tool should be used for initial and continuous assessment of workplace redesign. This will increase the ability of hospitals to identify and prioritize learning and infrastructure needs, while also identifying initiative-wide gaps and exemplars for systematic improvement and recognition. Drawing on the results of the gap analysis, each hospital will be responsible for tailoring and individualizing the interventions to their hospital-specific context and developing a concrete, written action plan that will be monitored and executed during the course of the intervention period. Results of the gap analysis and the effect of tailored interventions will serve as feedback to enhance learning.

The second intervention component consists of one-to-one twinning between European hospitals aspiring to implement the Magnet® model and longstanding US Magnet® designated hospitals. This seeks to promote capacity building through transfer of knowledge, skills, tools, technologies and best practices, thereby supporting positive reciprocal organizational changes.[55] An equal number of U.S. Magnet® hospitals – recognized for having an excellent work environment – will be recruited; allowing for a 1:1 twinning ratio of EU intervention hospitals with U.S. Magnet Hospitals. The twinning pairs will meet every two weeks virtually and two face-to-face mentoring sessions are planned each year when travel is safe. Face-to-face meetings between both parties allow for a profound, impact- and

For peer review only - http://bmjopen.bmj.com/site/about/guidelines.xhtml

meaningful experience that maximises the effect of the capacity building activities facilitating the intended organizational redesign.

Third, learning collaboratives will take place throughout the duration of the project and will be accessed and attended by all European hospitals in which the intervention is taking place at that point in time. The learning collaboratives will also involve policymakers so as to promote sustainability. Learning collaboratives build on the concept of communities of practice,[56] involving people coming together to engage in a process of learning in a shared domain of interest. These will be held monthly using remote means of communication with the possibility of in-person meetings annually when travel is safe. Effective elements of collaboratives, i.e. learning sessions, action periods and a collaborative extranet, will be implemented.[57–59]

Fourth is the creation of a critical mass of participating hospitals promoting innovation, attracting public interest, engaging stakeholders, and fostering replication and scaling of the intervention. Achieving a critical mass is essential for sustainability and scale-up of the intervention.[60] Critical mass shifts the context from one in which the change activities are unusual to one in which they are the norm. This long-standing principle in organizational sociology suggests that organizations become more open to and actively seek out change as the intervention gains legitimacy among peer institutions; this is particularly true in complex workplaces such as those found in health care.[61,62] Critical mass also maximizes the network exchanges and opportunities for feedback that arise out of other elements of our intervention such as the learning collaborative. This is supported by recent experience with the Magnet® model.[63,64]

A fifth component of the intervention is to provide regular survey feedback to hospitals on clinicians' reports on work conditions and well-being on the hospital level, separately for physicians and nurses.[65] Hospitals will be anonymized using a randomly generated letter code allowing each hospital to identify themselves, but not any of the other hospitals. Data will only be reported in aggregated form and will not highlight a single individual's data.

For peer review only - http://bmjopen.bmj.com/site/about/guidelines.xhtml

Evaluation of the effect of the intervention

A mixed-methods evaluation will be conducted to examine the effect of the intervention, including quantitative and qualitative components.

The guiding framework for our quantitative evaluation is the Quality Health Outcomes Model (QHOM) – as adapted by Aiken and colleagues[66] – based on a conceptual framework that sets out mechanisms relating organizational attributes to clinician and patient outcomes. Their model is theoretically founded on Donabedian's early conception of associations between structures, processes and outcomes[67] including the dynamism between model components that was proposed in the Health Quality Outcomes Model.[68] The Job Demands-Resources (JD-R) model also informed the choice of survey items, seeking to understand the intervention's impact on the imbalance between demands on the clinician and the resources available to clinicians to deal with these demands.[69] Job demands refer to aspects of the job that require sustained physical or mental effort and are associated with certain physiological and psychological costs. Job resources describe aspects of the job that are related to achieving work goals, reducing job demands and the associated physiological and psychological costs, and stimulating personal growth and development.

The qualitative part of the study aims first, to evaluate the implementation process, adaptation, reach and quality as well as mechanisms of impact manifested in participants' responses, mediators, unexpected pathways and consequences; and second, to explore barriers and enablers of the implementation and the experiences of the change process from the perspectives of health professionals and persons involved in leading the implementation.[70]

Quantitative evaluation

Quantitative data collection

Quantitative data collection across countries is standardized using an online data collection platform. Quantitative survey data will be collected at three points in time, i.e., at baseline (T0) in months 11-12 of the project timeline, at T1 (months 28-29) and T2 (months 41-42) (Figure 1) during an 8-week period. An open cohort design will be employed in which all eligible nurses and physicians are invited to

For peer review only - http://bmjopen.bmj.com/site/about/guidelines.xhtml

participate in each of these three measurement points (T0 – T2). Nurses and physicians will be recruited within each hospital at baseline and will be asked to participate in surveys with repeated measures taken over the three points in time allowing partially longitudinal follow-up of a cohort of individuals. Initial non-participants and new employees will be invited to join the study at later measurement points. Regular reminders will be sent to any clinicians (registered on the platform) through the online data collection platform who have not or only partially completed the survey. Individual informed consent for participation will be obtained by the researchers at the first contact (supplementary file 2). Each record of informed consent is retained on the online platform.

Quantitative measures

The main survey for nurses and physicians contains a core set of outcomes and main explanatory and process variables directly linked to the Magnet4Europe Magnet® Gap Analysis Tool. All study measures are based on validated instruments.

All survey items will be translated in Dutch, English, German, Norwegian and Swedish. When available, validated translations of instruments will be used. If no validated translation is available, a pre-specified translation procedure will be applied. First, a forward translation will be performed by a researcher involved in this research; the language in which the item is translated is the native language of the researcher. Secondly, other researchers within the country research group will thoroughly assess the forward translation in multiple phases until consensus is researched on each item.

Primary outcome

Burnout is the primary outcome measure and will be measured on individual level using the 9-item Emotional Exhaustion (EE) subscale of the Maslach Burnout Inventory (MBI).[71] In addition, the Burnout Assessment Tool (BAT), a 12-item novel self-report questionnaire will also be used to measure burnout.[72–74] The combination of both instruments to assess burnout, allows for: (1) a direct comparison of levels of emotional exhaustion with previous studies in the medical field (using the EE-subscale of MBI); (2) an up-to-date assessment of the more comprehensive burnout syndrome, as well

For peer review only - http://bmjopen.bmj.com/site/about/guidelines.xhtml

For peer review only

as a reliable estimation of the number of respondents with severe burnout symptoms and a comparison with the level of burnout of the national workforce.

Covariates

Secondary outcome measures include work engagement (UWES-3),[75] job satisfaction,[76,77] depression (PHQ-2),[78] anxiety (GAD-2),[79] general health (SF-8),[80] sleep quality (PSQ),[81] intent to leave the hospital,[76,77] absenteeism and presenteeism (HPQ),[14] workability, work-life conflict, team commitment, organizational commitment,[82] and whether clinicians would recommend the hospital.

Core exploratory variables include work environment (PES-NWI),[83] various measures of staffing and workload (NPQS),[84] care left undone,[85] operational failures, emotional demands (SIMPH),[86] red tape,[87] role conflicts (NPQS), job control, skill use, value congruence, performance feedback, opportunities for learning and development[88] and intrinsic motivation (WEIMS).[89]

Supplementary survey items are used to test more specific hypotheses.[69,90] These items contain measures on qualitative job insecurity [91], emotional dissonance,[92] task variety (QEEW),[93] and role clarity (NQPS),[84] basic need satisfaction,[94] and engaging leadership.[95]

Productivity measures consist of staff turnover rate, sickness absence rate, number of vacancies, and the use of agency staff - measured at organization level - complemented by previously mentioned secondary outcome measures, e.g., absenteeism and presenteeism are measured at individual level by means of a survey. Productivity measures evaluated at the level of the organization are evaluated over the period of one calendar year. The timeframe to evaluate absenteeism on individual level is 28 days, participants are asked to indicate the number of scheduled work days they have missed during the past 28 days. The timeframe to evaluate presenteeism on individual level is one year.

Data analysis

Regression models to estimate the association between the intervention and outcomes will include fixed effects of time, intervention, and intervention by time. Hospitals represent higher order units of

For peer review only - http://bmjopen.bmj.com/site/about/guidelines.xhtml

analysis, and clinicians the lower order unit of analysis. Countries will be treated as fixed effects. Repeated measures on nurses and physicians will also be appropriately modelled. More complex multilevel structural models will be employed to examine indirect and moderated associations between the process measures which we would expect to respond to the intervention and our outcome measures. All analyses will be conducted according to intention-to-treat. Data on hospital characteristics (e.g., size, teaching status) and clinician (e.g., gender, age) will be collected and used to adjust for potential confounders in the regression analyses. Statistical significance will be assessed at the 5% level. Occasional missing data will be limited by the electronic survey implementation using a force response option for each item. Data analysis will be conducted using SAS software, version 9.4 (and subsequent releases) of the SAS system for windows (SAS Institute Inc., Cary, NC, USA).

Process evaluation

Qualitative data collection

Data will be collected on context, implementation, and mechanisms of impact. Based on the results at baseline measurement, a purposive sample of two hospitals per country will be selected from the immediate intervention group for case studies to explore different aspects of the implementation process. Hospitals with the lowest burnout scores among clinicians will be contrasted with hospitals with the highest to explore potential differences in motivation and ambition in the hospitals. In total, four qualitative data collection periods are planned throughout the project (Figure 1). Two focus group interviews (one with 6-8 nurses, one with 6-8 physicians) will be conducted in each of the two case hospitals (per country) at one point in time (M34-35). This will generate 20 focus groups in total across all countries. A purposive sampling approach is used to recruit participants. We will invite physicians and nurses who work in direct patient care in clinical areas targeted by the Magnet intervention and most closely involved in implementing change. By doing so, we will aim for diversity within each staff group in terms of clinical areas and length of clinical experience. If focus groups cannot be convened (for example, recruitment difficulties), then each focus group will be replaced with 2 to 3 one-to-one interviews with similar staff types to the target focus group sample. Depending on practicalities, this

For peer review only - http://bmjopen.bmj.com/site/about/guidelines.xhtml

may result in a dataset that is generated from focus groups and/or one-to-one interviews. A minimum of 40 and maximum of 160 individuals will be involved in this part of the study.

In addition to the focus groups, two semi-structured individual interviews will be conducted at three time-points (months 23, 34 and 44) in each of the two case hospitals (per country). Persons directly involved in leading the implementation, i.e., the Magnet4Europe intervention coordinator, as well as another member of the interdisciplinary Magnet council will be invited to participate. This will lead to 60 interviews in total (across countries) over the course of the project. Where possible, we aim to interview the same two people across all time-points. If individuals leave the study, we will aim to recruit a replacement with a similar role and professional background. We will also aim for maximum diversity in professional backgrounds, e.g., one from nursing, one from medicine. A minimum of 30 and a maximum of 60 individuals will be involved in this part of the study.

To complement the qualitative evaluation, the 12-item Organizational Readiness for Implementing Change scale (ORIC)[96] and the 23-item Normalization Measure Development Questionnaire (NoMAD)[97,98] will be used to explore the readiness for change and the extent to which Magnet is integrated and sustained in the hospital over time. This will be done after the start of the intervention and at various time points. Magnet4Europe intervention coordinators and all other members of the interdisciplinary Magnet4Europe council in all hospitals will be invited to respond to the surveys. A minimum of 54 and a maximum of 324 individuals across all countries will be involved in this part of the study at each time point. If individuals leave and are replaced, their replacements will be invited to complete the survey on the next occasion.

Qualitative data analysis

Analysis of the qualitative data generated through focus groups and individual interviews will be guided by the Framework Analysis approach.[99] Data collection and analysis will be conducted by researchers in each of the Magnet4Europe countries, using a common coding template which will be developed based on initial themes. International meetings will be held regularly to discuss analysis and

For peer review only - http://bmjopen.bmj.com/site/about/guidelines.xhtml

findings. NVivo 12 (and subsequent releases) will be used for management and analysis of qualitative data.[100]

Data generated through collection of policy documents and general hospital information about context will be analysed through content analysis and/or discourse analysis[101] to explore different aspects of the organizational context in relation to the implementation process.

In the process evaluation we will analyse how organizational context links to the impact and sustainability of the Magnet programme. Emerging themes from the qualitative analysis will be used, together with the results from descriptive analyses of our quantitative measurements of readiness for change and of implementation normalization (NoMAD instrument), to explain variations in implementation between and within individual settings over time (Table 1).

Table 1 - Magnet4Europe Implementation Evaluation

**Context**<br>▪ General information about the hospital (e.g., type of hospital, size, organizational models, professional structure)<br>▪ Hospital policy documents (e.g., about staffing, work environment, leadership development, organizational plans)<br>▪ General country information (e.g., relevant legislation, financing of the healthcare sector)
**Implementation**<br>▪ Results from the Magnet4Europe Magnet® Gap Analysis Tool and the work plan of each hospital<br>▪ Implementation process survey data<br>▪ Data from interviews and focus groups
**Mechanisms of impact**<br>▪ Hospital policy documents (e.g., staffing, work environment, leadership development, organizational plans)<br>▪ Implementation process survey data<br>▪ Data from interviews and focus groups

Patient and public involvement

For peer review only - http://bmjopen.bmj.com/site/about/guidelines.xhtml

Patients and/or public were not involved in the design or development of research questions of this study.

ETHICS AND DISSEMINATION

Ethics

This study was approved by the Ethics Committee Research UZ/KU Leuven, Belgium; additionally, ethics approval is obtained in all other participating countries either through a central or decentral authority, to conduct nurse surveys as well as collect qualitative data. Any protocol amendments will be promptly reported to all relevant parties. An Ethical Advisory Committee was installed at the start of project and includes an independent ethics advisor with documented expertise to monitor ethics issues. The study is registered in the ISRCTN registry (ISRCTN10196901).

Dissemination

Magnet4Europe has a strong focus on disseminating evidence on the impact of work environment on mental health of health professionals. Findings will be published in peer reviewed journals and through other channels designed to reach a diverse community of researchers, practitioners, and other stakeholders. Gold open access will be used for the key findings of the study to ensure maximum exposure to a practice community. Beyond the funded life of the project the aim is to maximize accessibility by fully exploiting the opportunity for green open access and, where possible, identifying additional funds to support further gold open access publishing. Data management is compliant in line with the European Commission's Horizon 2020 Data Management Plan guidelines and FAIR-principles.

For peer review only

DISCUSSION

This paper describes the theoretical framework, conceptual design and methodological approaches of the EU H2020 Magnet4Europe project, which aims to evaluate the transfer, implementation, scale up and cost-effectiveness of the - Magnet® model of organizational redesign in a European context as a system-level approach to improve clinician well-being. Mental health and well-being is among one of the highest priorities of the public health agenda in the European Union.[1] This is based on the growing awareness and recognition of the magnitude of mental health problems and the burden on individuals, society, and economy. With the extraordinary conditions created by the coronavirus pandemic (COVID-19), this issue has taken an even more central role in the policy landscape.[102] COVID-19 is expected to have psychological impacts for health professional, in particular frontline workers that will be sustained over time.[103–106]

The research activities conducted within the project are expected to improve significantly the work environment of health professionals in hospitals, especially after the COVID-19 pandemic. In particular, Magnet4Europe proposes an organizational intervention, targeting the root of many of the problems (i.e. organizational and work-related stressors) facing health systems rather than focusing narrowly of their symptoms. It does so by means of a multi-centre study involving more than 60 hospitals across six European countries. By choosing a cluster randomized controlled design, this study is the first with the power to identify a causal association between implementing healthy workplace characteristics and improved staff wellbeing in European hospitals on a large scale. Two international pilot initiatives testing the transferability of Magnet key principles and accompanied by rigorous evaluations have informed the design of Magnet4Europe.[46,47] In both, the intervention hospitals improved their work environments substantially from their baselines at the end of the two-year intervention, and outcomes for staff improved. The Magnet4Europe intervention builds upon lessons learned from these pilot initiatives. The degree of innovation can also be seen in the use of a multi-faceted implementation strategy involving one-to-one twinning with international Magnet®-designated hospitals, creation of a critical mass, co-creation of solutions, development of learning collaboratives, benchmarking, and

For peer review only - http://bmjopen.bmj.com/site/about/guidelines.xhtml

interdisciplinary and management buy-in. In addition, Magnet4Europe makes a strong scientific contribution as it goes beyond traditional studies which focus solely on individual professions and disciplines separately. This allows us to study the interaction between physicians and nurses working in teams and the impact of healthy environments on their well-being and patient outcomes.

Magnet4Europe will also allow countries to learn from the experience of other health systems and understand the factors influencing their sustainability, as the study will be undertaken in six European countries representing the two dominant health system forms. The so-called Beveridge countries (England, Ireland, Sweden, Norway) are tax funded resulting in potentially more government health policy decision-making while the so-called Bismarck countries (Belgium, Germany) have an employer-based social insurance financing system, which potentially allows for more organizational variation. This study allows us to compare and contrast the impact of health care systems on the implementation and sustainability of the intervention.

The project involves dissemination and stakeholder engagement. Various stakeholders will be involved in the project including designees from international and European organizations who represent nursing professionals, health care employers, patients, and governments. Their main role is to raise awareness of the project and to support dissemination of the results as well as the Magnet4Europe consortium in formulating policy recommendations based on the scientific results.

Limitations

Contextual factors inherent to the countries where Magnet4Europe will be implemented, more specifically different health systems within and across countries, may impact the potential for uptake of the intervention and unintentionally lead to variation in the intervention and outcomes. Certain health care system characteristics such as labour supply policies, education requirements and funding schemes may interact with the intervention. In predominantly Beveridge type system countries, the interaction between these characteristics and the intervention may lead to a more cumbersome

For peer review only - http://bmjopen.bmj.com/site/about/guidelines.xhtml

process of change and innovation compared to countries with a more decentralised insurance-based health system.

Despite having a strong conceptual design for the Magnet4Europe study, i.e. a cluster randomized controlled trial, bearing many advantages and having the potential to facilitate the robust evaluation of this type of intervention, this design is susceptible to multiple sources for potential risk of bias. First, contamination of the wait-list control group cannot be ruled out. All hospitals were, prior to the start of the intervention, informed about the intervention and the active components. It was not possible to blind hospitals to their allocation in either the immediate intervention group or wait-list control group. We aimed to mitigate contamination of hospitals assigned to the wait-list control group by not providing them with the Magnet blueprint of organisational redesign and by not disclosing the name of their future Magnet® twinning partner to avoid early contact and exchange of information prior to their actual start of the intervention. A second additional risk related to contamination is when the intervention takes longer to be fully embedded as intended. The intervention proposed in Magnet4Europe is a complex intervention at the hospital level, earlier research findings demonstrated that a considerable amount of time is required for the intervention to become fully embedded into practice and influence outcomes accordingly. Due to the limited time frame available in Magnet4Europe, no transition period is incorporated. This potential risk is mitigated by two intervention components, i.e. the one-to-one twinning of the European intervention hospitals with a designated and experienced US Magnet® hospital and the Learning Collaboratives. Both components are expected to act as a catalysator for knowledge transfer and innovation and expedite the implementation of the intervention. There is a potential risk that the true effect of the intervention only becomes apparent after the formal ending of the four-year study period. A longer follow-up of the study beyond the funding is foreseen.

For peer review only - http://bmjopen.bmj.com/site/about/guidelines.xhtml

LIST OF FIGURES

1. Figure 1 – Magnet4Europe timeline. Legend: † - Activity for group 1; ‡ - Activity for group 2; * - Activity for both groups

AUTHORS' CONTRIBUTIONS

WS, LA, MMH and LB devised the study. WS, LA, MMH and LB drafted the protocol, with assistance from JB, JB, RB, HDW, SD, JD, LEE, PG, DK, JK, RL, CM, MMK, AMR, WBS, DS, LSA, HS. All authors provided edits and reviewed the manuscript for important intellectual content. All authors gave final approval of the version to be published and agreed to be accountable for all aspects of this work.

FUNDING

This study is funded by the European Union's Horizon 2020 research and innovation programme under the project Magnet4Europe: Improving Mental Health and Wellbeing in the Health Care Workplace (Grant Agreement No. 848031), starting 1st January 2020 until 31st December 2023. The materials presented here are the responsibility of the authors only. The EU Commission takes no responsibility for any use made of the information set out.

COMPETING INTERESTS

None declared.

ACKNOWLEDGMENTS

We thank the American Nurses Credentialing Center (ANCC) for granting the right to use Magnet® model as core element of the Magnet4Europe intervention. The Magnet® model is a trademark of ANCC registered in the United States of America and other jurisdictions, and is being used under license from ANCC. All rights are reserved by ANCC. ANCC's consent to the use of the Intervention Hospital Magnet® mark shall not be construed as ANCC sponsoring, participating, or endorsing the

For peer review only

Magnet4Europe intervention. ©American Nurses Credentialing Center. Reproduced under license from the American Nurses Credentialing Center. All rights reserved.

MAGNET4EUROPE CONSORTIUM

The Magnet4Europe Consortium consists of Walter Sermeus (director), Luk Bruyneel, Hans De Witte, Wilmar B. Schaufeli, Simon Dello, Dorothea Kohnen (Belgium, Catholic University Leuven); Linda Aiken (codirector), Matthew McHugh, Mary Del Guidice, Herbert Smith, Timothy Cheney, Douglas Sloane (USA, University of Pennsylvania); Reinhard Busse, Claudia Maier, Julia Köppen, Joan Kleine (Germany, Technical University Berlin); Jonathan Drennan, Vera McCarthy, Elaine Lehane, Noeleen Brady, Anne Scott (Ireland, University College Cork); Ingeborg Strømseng Sjetne (Norway, Norwegian Institute of Public Health); Anners Lerdal, Monica Bukkøy Kjetland (Norway, Lovisenberg Diaconal Hospital); Lars E. Eriksson, Rikard Lindqvist, Lisa Smeds Alenius (Sweden, Karolinska Institutet); Jane Ball, Peter Griffiths, Jackie Bridges, Sydney Anstee (England, University of Southampton); Anne Marie Rafferty (England, King's College London); Martin McKee (England, London School of Hygiene and Tropical Medicine); Paul Van Aken, Danny Van Heusden, Kaat Siebens, Peter Van Bogaert (Belgium, University Hospital Antwerp).

For peer review only

REFERENCES

1 Europa-Kommisisonen. GREEN PAPER- Improving the mental health of the population Towards a strategy on mental health for the European Union. OOPEC 2005. http://ec.europa.eu/health/ph_determinants/life_style/mental/green_paper/mental_gp_en.pdf

2 Health M, The IN, Europe IN. Eu Compass for Action on Mental Health and Well-Being Mental Health in the Workplace. 2020.

3 Kim M-S, Kim T, Lee D, *et al.* Mental disorders among workers in the healthcare industry: 2014 national health insurance data. *Ann Occup Environ Med* 2018;**30**:31. doi:10.1186/s40557-018-0244-x

4 Shanafelt TD, Boone S, Tan L, *et al.* Burnout and satisfaction with work-life balance among US physicians relative to the general US population. *Arch Intern Med* 2012;**172**:1377–85. doi:10.1001/archinternmed.2012.3199

5 Aiken LH, Sloane DM, Bruyneel L, *et al.* Nurse staffing and education and hospital mortality in nine European countries: A retrospective observational study. *Lancet* 2014;**383**:1824–30. doi:10.1016/S0140-6736(13)62631-8

6 Aiken LH, Sermeus W, Van Den Heede K, *et al.* Patient safety, satisfaction, and quality of hospital care: Cross sectional surveys of nurses and patients in 12 countries in Europe and the United States. *BMJ* 2012;**344**:e1717. doi:10.1136/bmj.e1717

7 Dyrbye LN, Burke SE, Hardeman RR, *et al.* Association of clinical specialty with symptoms of burnout and career choice regret among US resident physicians. *JAMA - J Am Med Assoc* 2018;**320**:1114–30. doi:10.1001/jama.2018.12615

8 Rotenstein LS, Torre M, Ramos MA, *et al.* Prevalence of burnout among physicians a systematic review. *JAMA - J Am Med Assoc* 2018;**320**:1131–50. doi:10.1001/jama.2018.12777

For peer review only - http://bmjopen.bmj.com/site/about/guidelines.xhtml

9 Van Gerven E, Vander Elst T, Vandenbroeck S, *et al.* Increased risk of burnout for physicians and nurses involved in a patient safety incident. *Med Care* 2016;**54**:937–43. doi:10.1097/MLR.0000000000000582

10 Vandenbroeck S, Van Gerven E, De Witte H, *et al.* Burnout in Belgian physicians and nurses. *Occup Med (Chic Ill)* 2017;**67**:546–54. doi:10.1093/occmed/kqx126

11 Schernhammer E. Taking Their Own Lives — The High Rate of Physician Suicide. *N Engl J Med* 2005;**352**:2473–6. doi:10.1056/nejmp058014

12 Dyrbye LN, Thomas MR, Massie FS, *et al.* Burnout and suicidal ideation among U.S. medical students. *Ann Intern Med* 2008;**149**:334–41. doi:10.7326/0003-4819-149-5-200809020-00008

13 Schwenk TL, Davis L, Wimsatt LA. Depression, stigma, and suicidal ideation in medical students. *JAMA - J Am Med Assoc* 2010;**304**:1181–90. doi:10.1001/jama.2010.1300

14 Kessler RC, Barber C, Beck A, *et al.* The World Health Organization Health and Work Performance Questionnaire (HPQ). *J Occup Environ Med* 2003;**45**:156–74. doi:10.1097/01.jom.0000052967.43131.51

15 Davis MA, Cher BAY, Friese CR, *et al.* Association of US Nurse and Physician Occupation with Risk of Suicide. *JAMA Psychiatry* 2021;**78**:651–8. doi:10.1001/jamapsychiatry.2021.0154

16 Shanafelt TD, Balch CM, Bechamps G, *et al.* Burnout and medical errors among American surgeons. *Ann Surg* 2010;**251**:995–1000. doi:10.1097/SLA.0b013e3181bfdab3

17 Salyers MP, Bonfils KA, Luther L, *et al.* The Relationship Between Professional Burnout and Quality and Safety in Healthcare: A Meta-Analysis. *J Gen Intern Med* 2017;**32**:475–82. doi:10.1007/s11606-016-3886-9

18 Dewa CS, Loong D, Bonato S, *et al.* The relationship between physician burnout and quality of healthcare in terms of safety and acceptability: A systematic review. *BMJ Open*

For peer review only

For peer review only - http://bmjopen.bmj.com/site/about/guidelines.xhtml

2017;**7**:e015141. doi:10.1136/bmjopen-2016-015141

19 Dewa CS, Jacobs P, Thanh NX, *et al.* An estimate of the cost of burnout on early retirement and reduction in clinical hours of practicing physicians in Canada. *BMC Health Serv Res* 2014;**14**:254. doi:10.1186/1472-6963-14-254

20 Muller AE, Hafstad EV, Himmels JPW, *et al.* The mental health impact of the covid-19 pandemic on healthcare workers, and interventions to help them: A rapid systematic review. Psychiatry Res. 2020;**293**:113441. doi:10.1016/j.psychres.2020.113441

21 Vanhaecht K, Seys D, Bruyneel L, *et al.* COVID-19 is having a destructive impact on health-care workers' mental well-being. *Int J Qual Heal Care* Published Online First: 3 December 2020. doi:10.1093/intqhc/mzaa158

22 Tan BYQ, Chew NWS, Lee GKH, *et al.* Psychological Impact of the COVID-19 Pandemic on Health Care Workers in Singapore. *Ann Intern Med* 2020;**173**:317–20. doi:10.7326/M20-1083

23 Pfefferbaum B, North CS. Mental Health and the Covid-19 Pandemic. *N Engl J Med* 2020;**383**:510–2. doi:10.1056/nejmp2008017

24 Walton M, Murray E, Christian MD. Mental health care for medical staff and affiliated healthcare workers during the COVID-19 pandemic. *Eur Hear J Acute Cardiovasc Care* 2020;**9**:241–7. doi:10.1177/2048872620922795

25 European Union. European Framework for Action on Mental Health and Wellbeing. 2016. http://www.mentalhealthandwellbeing.eu/assets/docs/publications/Framework for action_19jan (1)-20160119192639.pdf

26 Panagioti M, Panagopoulou E, Bower P, *et al.* Controlled interventions to reduce burnout in physicians a systematic review and meta-analysis. JAMA Intern. Med. 2017;**177**:195–205. doi:10.1001/jamainternmed.2016.7674

For peer review only - http://bmjopen.bmj.com/site/about/guidelines.xhtml

27 West CP, Dyrbye LN, Erwin PJ, *et al.* Interventions to prevent and reduce physician burnout: a systematic review and meta-analysis. *Lancet* 2016;**388**:2272–81. doi:10.1016/S0140-6736(16)31279-X

28 West CP, Dyrbye LN, Shanafelt TD. Physician burnout: contributors, consequences and solutions. *J Intern Med* 2018;**283**:516–29. doi:10.1111/joim.12752

29 Kutney-Lee A, Wu ES, Sloane DM, *et al.* Changes in hospital nurse work environments and nurse job outcomes: an analysis of panel data. *Int J Nurs Stud* 2013;**50**:195–201. doi:10.1016/j.ijnurstu.2012.07.014

30 Shanafelt TD, Gorringe G, Menaker R, *et al.* Impact of organizational leadership on physician burnout and satisfaction. *Mayo Clin Proc* 2015;**90**:432–40. doi:10.1016/j.mayocp.2015.01.012

31 Diez-Pinol M, Dolan SL, Sierra V, *et al.* Personal and organizational determinants of well-being at work: The case of Swedish physicians. *Int J Health Care Qual Assur* 2008;**21**:598–610. doi:10.1108/09526860810900754

32 Hayes LJ, O'Brien-Pallas L, Duffield C, *et al.* Nurse turnover: A literature review - An update. *Int J Nurs Stud* 2012;**49**:887–905. doi:10.1016/j.ijnurstu.2011.10.001

33 Hayes LJ, O'Brien-Pallas L, Duffield C, *et al.* Nurse turnover: a literature review. *Int J Nurs Stud* 2006;**43**:237–63. doi:10.1016/j.ijnurstu.2005.02.007

34 Buerhaus PI. Current and future State of the US nursing workforce. *JAMA - J Am Med Assoc* 2008;**300**:2422–4. doi:10.1001/jama.2008.729

35 Upenieks V V. The interrelationship of organizational characteristics of magnet hospitals, nursing leadership, and nursing job satisfaction. *Health Care Manag (Frederick)* 2003;**22**:83–98. doi:10.1097/00126450-200304000-00002

36 Magnet hospitals. Attraction and retention of professional nurses. Task Force on Nursing

For peer review only - http://bmjopen.bmj.com/site/about/guidelines.xhtml

Practice in Hospitals. American Academy of Nursing. *ANA Publ* 1983;:i–xiv, 1–135.

37 Blegen MA, Goode CJ, Spetz J, *et al.* Nurse staffing effects on patient outcomes safety-net and non-safety-net hospitals. *Med Care* 2011;**49**:406–14. doi:10.1097/MLR.0b013e318202e129

38 Kelly LA, McHugh MD, Aiken LH. Nurse Outcomes in Magnet® and Non-Magnet Hospitals. *JONA J Nurs Adm* 2012;**42**:S44–9. doi:10.1097/01.NNA.0000420394.18284.4f

39 Kutney-Lee A, Stimpfel AW, Sloane DM, *et al.* Changes in patient and nurse outcomes associated with magnet hospital recognition. *Med Care* 2015;**53**:550–7. doi:10.1097/MLR.0000000000000355

40 Drenkard K. The business case for magnet®. *J Nurs Adm* 2010;**40**:263–71. doi:10.1097/NNA.0b013e3181df0fd6

41 Jayawardhana J, Welton JM, Lindrooth RC. Is there a business case for magnet hospitals? Estimates of the cost and revenue implications of becoming a magnet. *Med Care* 2014;**52**:400–6. doi:10.1097/MLR.0000000000000092

42 Stimpfel AW, Sloane DM, McHugh MD, *et al.* Hospitals Known for Nursing Excellence Associated with Better Hospital Experience for Patients. *Health Serv Res* 2016;**51**:1120–34. doi:10.1111/1475-6773.12357

43 Aiken LH, Sloane DM, Ball J, *et al.* Patient satisfaction with hospital care and nurses in England: An observational study. *BMJ Open* 2018;**8**:e019189. doi:10.1136/bmjopen-2017-019189

44 McHugh MD, Kelly LA, Smith HL, *et al.* Lower mortality in magnet hospitals. *Med Care* 2013;**51**:382–8. doi:10.1097/MLR.0b013e3182726cc5

45 Barnes H, Rearden J, Mchugh MD. Magnet® Hospital Recognition Linked to Lower Central Line-Associated Bloodstream Infection Rates. *Res Nurs Heal* 2016;**39**:96–104.

For peer review only - http://bmjopen.bmj.com/site/about/guidelines.xhtml

doi:10.1002/nur.21709

46 Aiken LH, Buchan J, Ball J, *et al.* Transformative impact of Magnet designation: England case study. *J Clin Nurs* 2008;**17**:3330–7. doi:10.1111/j.1365-2702.2008.02640.x

47 Aiken LH, Poghosyan L. Evaluation of 'magnet journey to nursing excellence program' in Russia and Armenia. *J Nurs Scholarsh* 2009;**41**:166–74. doi:10.1111/j.1547-5069.2009.01268.x

48 Aunger JA, Millar R, Rafferty AM, *et al.* How, when, and why do inter-organisational collaborations in healthcare work? A realist evaluation. *PLoS One* 2022;**17**:e0266899. doi:10.1371/JOURNAL.PONE.0266899

49 Chan A-W, Tetzlaff JM, Altman DG, *et al.* SPIRIT 2013 statement: defining standard protocol items for clinical trials. *Ann Intern Med* 2013;**158**:200–7.

50 Kim HW, Chung CH. Mitral-aortic intervalvular fibrosa pseudoaneurysm resulting in the displacement of the left main coronary artery after aortic valve replacement. *J Thorac Cardiovasc Surg* 2010;**139**. doi:10.1016/j.jtcvs.2008.07.045

51 Hemming K, Taljaard M. Sample size calculations for stepped wedge and cluster randomised trials: A unified approach. *J Clin Epidemiol* 2016;**69**:137–46. doi:10.1016/j.jclinepi.2015.08.015

52 Woertman W, De Hoop E, Moerbeek M, *et al.* Stepped wedge designs could reduce the required sample size in cluster randomized trials. *J Clin Epidemiol* 2013;**66**:752–8. doi:10.1016/j.jclinepi.2013.01.009

53 Maslach C, Jackson SE. The measurement of experienced burnout. *J Organ Behav* 1981;**2**:99–113. doi:10.1002/job.4030020205

54 Li B, Bruyneel L, Sermeus W, *et al.* Group-level impact of work environment dimensions on burnout experiences among nurses: a multivariate multilevel probit model. *Int J Nurs Stud* 2013;**50**:281–91. doi:10.1016/j.ijnurstu.2012.07.001

For peer review only - http://bmjopen.bmj.com/site/about/guidelines.xhtml

55 Cadée F, Nieuwenhuijze MJ, Lagro-Janssen ALM, *et al.* The state of the art of twinning, a concept analysis of twinning in healthcare. *Global Health* 2016;**12**:66. doi:10.1186/s12992-016-0205-5

56 Cuddy C. *Cultivating Communities of Practice: A Guide to Managing Knowledge*. Harvard Business School Press 2002. doi:10.1108/bl.2002.17015bae.001

57 Mills PD, Weeks WB. Characteristics of successful quality improvement teams: lessons from five collaborative projects in the VHA. *Jt Comm J Qual Saf* 2004;**30**:152–62. doi:10.1016/S1549-3741(04)30017-1

58 Nembhard IM. Learning and improving in quality improvement collaboratives: Which collaborative features do participants value most?: Quality and patient safety. *Health Serv Res* 2009;**44**:359–78. doi:10.1111/j.1475-6773.2008.00923.x

59 Yuan CT, Nembhard IM, Stern AF, *et al.* Blueprint for the dissemination of evidence-based practices in health care. *Issue Brief (Commonw Fund)* 2010;**86**:1–16.https://www.ncbi.nlm.nih.gov/pubmed/20469542

60 Greenhalgh T, Robert G, Macfarlane F, *et al.* Diffusion of innovations in service organizations: Systematic review and recommendations. *Milbank Q* 2004;**82**:581–629. doi:10.1111/j.0887-378X.2004.00325.x

61 Meyer AD, Goes JB. Organizational Assimilation of Innovations: A Multilevel Contextual Analysis. *Acad Manag J* 1988;**31**:897–923. doi:10.5465/256344

62 Kimberly JR, Evanisko MJ. Organizational innovation: the influence of individual, organizational, and contextual factors on hospital adoption of technological and administrative innovations. *Acad Manage J* 1981;**24**:689–713. doi:10.2307/256170

63 Richards MR, Lasater K, McHugh M. A race to the top? Competitive pressure and magnet adoption among us hospitals 1997-2012. *Med Care* 2017;**55**:384–90.

For peer review only - http://bmjopen.bmj.com/site/about/guidelines.xhtml

doi:10.1097/MLR.0000000000000650

64 Lasater KB, Richards MR, Dandapani NB, *et al.* Magnet hospital recognition in hospital systems over time. *Health Care Manage Rev* 2019;**44**:19–29. doi:10.1097/HMR.0000000000000167

65 Björklund C, Grahn A, Jensen I, *et al.* Does survey feedback enhance the psychosocial work environment and decrease sick leave? *Eur J Work Organ Psychol* 2007;**16**:76–93. doi:10.1080/13594320601112169

66 Aiken LH, Sochalski J, Lake ET. Studying outcomes of organizational change in health services. *Med Care* 1997;**35**:NS6-18. doi:10.1097/00005650-199711001-00002

67 Donabedian A. Evaluating the quality of medical care. *Milbank Q* 2005;**83**:691–729. doi:10.1111/j.1468-0009.2005.00397.x

68 Mitchell PH, Ferketich S, Jennings BM. Quality Health Outcomes Model. *J Nurs Scholarsh* 1998;**30**:43–6. doi:10.1111/j.1547-5069.1998.tb01234.x

69 Demerouti E, Nachreiner F, Bakker AB, *et al.* The job demands-resources model of burnout. *J Appl Psychol* 2001;**86**:499–512. doi:10.1037/0021-9010.86.3.499

70 Moore GF, Audrey S, Barker M, *et al.* Process evaluation of complex interventions: Medical Research Council guidance. *BMJ* 2015;**350**:h1258. doi:10.1136/bmj.h1258

71 Maslach C, Jackson SE. The measurement of experienced burnout. *J Organ Behav* 1981;**2**:99–113. doi:10.1002/job.4030020205

72 de Beer LT, Schaufeli WB, De Witte H, *et al.* Measurement invariance of the burnout assessment tool (Bat) across seven cross-national representative samples. *Int J Environ Res Public Health* 2020;**17**:1–14. doi:10.3390/ijerph17155604

73 Schaufeli WB, Desart S, De Witte H. Burnout assessment tool (Bat)—development, validity, and reliability. *Int J Environ Res Public Health* 2020;**17**:1–21. doi:10.3390/ijerph17249495

For peer review only - http://bmjopen.bmj.com/site/about/guidelines.xhtml

74 Hadžibajramović E, Schaufeli W, De Witte H. A Rasch analysis of the Burnout Assessment Tool (BAT). *PLoS One* 2020;**15**. doi:10.1371/journal.pone.0242241

75 Schaufeli WB, Shimazu A, Hakanen J, *et al.* An ultra-short measure for work engagement: The UWES-3 validation across five countries. *Eur J Psychol Assess* 2019;**35**:577–91. doi:10.1027/1015-5759/a000430

76 Wanous JP, Reichers AE, Hudy MJ. Overall job satisfaction: How good are single-item measures? *J Appl Psychol* 1997;**82**:247–52. doi:10.1037/0021-9010.82.2.247

77 Wanous JP, Hudy MJ. Single-Item Reliability: A Replication and Extension. *Organ Res Methods* 2001;**4**:361–75. doi:10.1177/109442810144003

78 Kroenke K, Spitzer R, Williams J. The Patient Health Questionnaire-2: Validity of a Two-Item Depression Screener. *Med Care* 2003;**41**:1284–92.

79 Kroenke K, Spitzer RL, Williams JBW, *et al.* Anxiety disorders in primary care: Prevalence, impairment, comorbidity, and detection. *Ann Intern Med* 2007;**146**:317–25. doi:10.7326/0003-4819-146-5-200703060-00004

80 Ware JE, GlaxoSmithKline. How to score and interpret single-item health status measures : a manual for users of the of the SF-8 health survey : (with a supplement on the SF-6 health survey). 2001;:220.http://www.worldcat.org/title/how-to-score-and-interpret-single-item-health-status-measures-a-manual-for-users-of-the-of-the-sf-8-health-survey-with-a-supplement-on-the-sf-6-health-survey/oclc/47005803

81 Buysse DJ, Reynolds CF, Monk TH, *et al.* The Pittsburgh sleep quality index: A new instrument for psychiatric practice and research. *Psychiatry Res* 1989;**28**:193–213. doi:10.1016/0165-1781(89)90047-4

82 Rahmadani VG, Schaufeli WB, Stouten J, *et al.* Engaging leadership and its implication for work engagement and job outcomes at the individual and team level: A multi-level longitudinal

For peer review only

study. *Int J Environ Res Public Health* 2020;**17**:776. doi:10.3390/ijerph17030776

83 Lake ET. Development of the practice environment scale of the nursing work index. *Res Nurs Heal* 2002;**25**:176–88. doi:10.1002/nur.10032

84 Lindström K, Elo A-L, Skogstad A, *et al. User ' S Guide for the QPS Nordic. General Nordic Questionnaire for Psychological and Social Factors at Work.* KÃ¸benhavn: : Nordic Council of Ministers 2000.

85 Aiken LH, Clarke SP, Sloane DM, *et al.* Nurses' reports on hospital care in five countries. *Health Aff* 2001;**20**:43–53. doi:10.1377/hlthaff.20.3.43

86 Notelaers G, De Witte H, Van Veldhoven M, *et al.* Construction and validation of the Short Inventory to Monitor Psychosocial Hazards. *Arb en Ergon* 2007;**44**:11–7.

87 van Loon NM, Leisink PLM, Knies E, *et al.* Red Tape: Developing and Validating a New Job-Centered Measure. *Public Adm Rev* 2016;**76**:662–73. doi:10.1111/puar.12569

88 Schaufeli WB. Applying the Job Demands-Resources model: A 'how to' guide to measuring and tackling work engagement and burnout. *Organ Dyn* 2017;**46**:120–32. doi:10.1016/j.orgdyn.2017.04.008

89 Tremblay MA, Blanchard CM, Taylor S, *et al.* Work Extrinsic and Intrinsic Motivation scale: its value for organizational psychology research. *Can J Behav Sci* 2009;**41**:213–26. doi:10.1037/a0015167

90 Bakker AB, Demerouti E. The Job Demands-Resources model: State of the art. *J Manag Psychol* 2007;**22**:309–28. doi:10.1108/02683940710733115

91 De Witte H, De Cuyper N, Handaja Y, *et al.* Associations between quantitative and qualitative job insecurity and well-being. *Int Stud Manag Organ* 2010;**40**:40–56. doi:10.2753/IMO0020-8825400103

For peer review only

For peer review only - http://bmjopen.bmj.com/site/about/guidelines.xhtml

92 Zapf D. Emotion work and psychological well-being. A review of the literature and some conceptual considerations. *Hum Resour Manag Rev* 2002;**12**:237–68. doi:10.1016/S1053-4822(02)00048-7

93 Van Veldhoven M, De Jonge J, Broersen S, *et al.* Specific relationships between psychosocial job conditions and job-related stress: A three-level analytic approach. *Work Stress* 2002;**16**:207–28. doi:10.1080/02678370210166399

94 Van den Broeck A, Vansteenkiste M, De Witte H, *et al.* Capturing autonomy, competence, and relatedness at work: Construction and initial validation of the Work-related Basic Need Satisfaction scale. *J Occup Organ Psychol* 2010;**83**:981–1002. doi:10.1348/096317909X481382

95 Schaufeli WB. Engaging leadership in the job demands-resources model. *Career Dev Int* 2015;**20**:446–63. doi:10.1108/CDI-02-2015-0025

96 Shea CM, Jacobs SR, Esserman DA, *et al.* Organizational readiness for implementing change: A psychometric assessment of a new measure. *Implement Sci* 2014;**9**. doi:10.1186/1748-5908-9-7

97 Rapley T, Girling M, Mair FS, *et al.* Improving the normalization of complex interventions: Part 1 - Development of the NoMAD instrument for assessing implementation work based on normalization process theory (NPT) 17 Psychology and Cognitive Sciences 1701 Psychology. *BMC Med Res Methodol* 2018;**18**:133. doi:10.1186/s12874-018-0590-y

98 Finch TL, Girling M, May CR, *et al.* Improving the normalization of complex interventions: Part 2 - Validation of the NoMAD instrument for assessing implementation work based on normalization process theory (NPT) 17 Psychology and Cognitive Sciences 1701 Psychology. *BMC Med Res Methodol* 2018;**18**. doi:10.1186/s12874-018-0591-x

99 Perreault K. Research Design: Qualitative, Quantitative, and Mixed Methods Approaches. *Man Ther* 2011;**16**:103. doi:10.1016/j.math.2010.09.003

For peer review only - http://bmjopen.bmj.com/site/about/guidelines.xhtml

100 Ltd. QIP. NVivo. 2020.https://www.qsrinternational.com/nvivo-qualitative-data-analysis-software/home

101 Bowen GA. Document analysis as a qualitative research method. *Qual Res J* 2009;**9**:27–40. doi:10.3316/QRJ0902027

102 Muller AE, Hafstad EV, Himmels JPW, *et al.* The mental health impact of the covid-19 pandemic on healthcare workers, and interventions to help them: A rapid systematic review. Psychiatry Res. 2020;**293**:113441. doi:10.1016/j.psychres.2020.113441

103 Blake H, Bermingham F, Johnson G, *et al.* Mitigating the psychological impact of covid-19 on healthcare workers: A digital learning package. *Int J Environ Res Public Health* 2020;**17**:2997. doi:10.3390/ijerph17092997

104 Sampaio F, Sequeira C, Teixeira L. Impact of COVID-19 outbreak on nurses' mental health: A prospective cohort study. *Environ Res* 2021;**194**:110620. doi:10.1016/j.envres.2020.110620

105 De Kock JH, Latham HA, Leslie SJ, *et al.* A rapid review of the impact of COVID-19 on the mental health of healthcare workers: implications for supporting psychological well-being. *BMC Public Health* 2021;**21**:1–18. doi:10.1186/s12889-020-10070-3

106 Greenberg N, Weston D, Hall C, *et al.* Mental health of staff working in intensive care during Covid-19. *Occup Med (Lond)* 2021;**71**:62–7. doi:10.1093/occmed/kqaa220

For peer review only

For peer review only - http://bmjopen.bmj.com/site/about/guidelines.xhtml

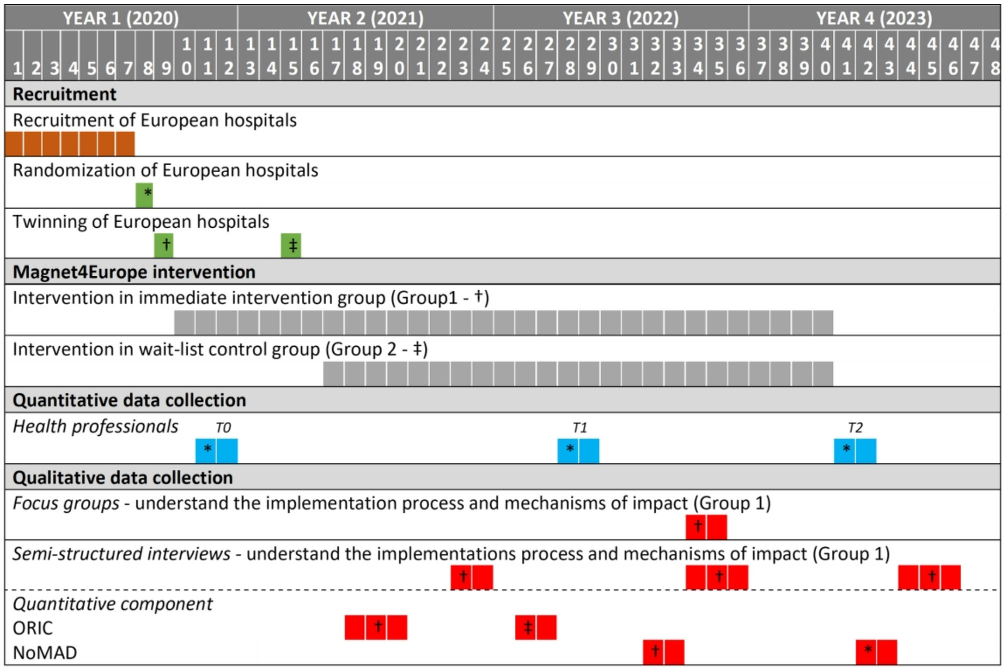

Figure 1 - Magnet4Europe timeline
† - Activity for group 1; ‡ - Activity for group 2; * - Activity for both groups

90x59mm (600 x 600 DPI)

SPIRIT

STANDARD PROTOCOL ITEMS: RECOMMENDATIONS FOR INTERVENTIONAL TRIALS

Supplementary File 1 - SPIRIT 2013 Checklist: Recommended items to address in a clinical trial protocol and related documents*

Section/item	Item No	Description	Addressed on page number
**Administrative information**			
Title	1	Descriptive title identifying the study design, population, interventions, and, if applicable, trial acronym	1
Trial registration	2a	Trial identifier and registry name. If not yet registered, name of intended registry	2, 17
	2b	All items from the World Health Organization Trial Registration Data Set	2, 6, 7
Protocol version	3	Date and version identifier	N/A
Funding	4	Sources and types of financial, material, and other support	20
Roles and responsibilities	5a	Names, affiliations, and roles of protocol contributors	1, 20
	5b	Name and contact information for the trial sponsor	20
	5c	Role of study sponsor and funders, if any, in study design; collection, management, analysis, and interpretation of data; writing of the report; and the decision to submit the report for publication, including whether they will have ultimate authority over any of these activities	20
	5d	Composition, roles, and responsibilities of the coordinating centre, steering committee, endpoint adjudication committee, data management team, and other individuals or groups overseeing the trial, if applicable (see Item 21a for data monitoring committee)	N/A

For peer review only

For peer review only - http://bmjopen.bmj.com/site/about/guidelines.xhtml

Introduction

Background and rationale	6a	Description of research question and justification for undertaking the trial, including summary of relevant studies (published and unpublished) examining benefits and harms for each intervention	4-5
	6b	Explanation for choice of comparators	4-5
Objectives	7	Specific objectives or hypotheses	6
Trial design	8	Description of trial design including type of trial (eg, parallel group, crossover, factorial, single group), allocation ratio, and framework (eg, superiority, equivalence, noninferiority, exploratory)	6

Methods: Participants, interventions, and outcomes

Study setting	9	Description of study settings (eg, community clinic, academic hospital) and list of countries where data will be collected. Reference to where list of study sites can be obtained	6
Eligibility criteria	10	Inclusion and exclusion criteria for participants. If applicable, eligibility criteria for study centres and individuals who will perform the interventions (eg, surgeons, psychotherapists)	6
Interventions	11a	Interventions for each group with sufficient detail to allow replication, including how and when they will be administered	9-12
	11b	Criteria for discontinuing or modifying allocated interventions for a given trial participant (eg, drug dose change in response to harms, participant request, or improving/worsening disease)	12
	11c	Strategies to improve adherence to intervention protocols, and any procedures for monitoring adherence (eg, drug tablet return, laboratory tests)	N/A
	11d	Relevant concomitant care and interventions that are permitted or prohibited during the trial	N/A
Outcomes	12	Primary, secondary, and other outcomes, including the specific measurement variable (eg, systolic blood pressure), analysis metric (eg, change from baseline, final value, time to event), method of aggregation (eg, median, proportion), and time point for each outcome. Explanation of the clinical relevance of chosen efficacy and harm outcomes is strongly recommended	12-16
Participant timeline	13	Time schedule of enrolment, interventions (including any run-ins and washouts), assessments, and visits for participants. A schematic diagram is highly recommended (see Figure)	8

For peer review only - http://bmjopen.bmj.com/site/about/guidelines.xhtml

Sample size	14	Estimated number of participants needed to achieve study objectives and how it was determined, including clinical and statistical assumptions supporting any sample size calculations	7
Recruitment	15	Strategies for achieving adequate participant enrolment to reach target sample size	7, 12

Methods: Assignment of interventions (for controlled trials)

Allocation:

Sequence generation	16a	Method of generating the allocation sequence (eg, computer-generated random numbers), and list of any factors for stratification. To reduce predictability of a random sequence, details of any planned restriction (eg, blocking) should be provided in a separate document that is unavailable to those who enrol participants or assign interventions	7-8
Allocation concealment mechanism	16b	Mechanism of implementing the allocation sequence (eg, central telephone; sequentially numbered, opaque, sealed envelopes), describing any steps to conceal the sequence until interventions are assigned	7-8
Implementation	16c	Who will generate the allocation sequence, who will enrol participants, and who will assign participants to interventions	7-8
Blinding (masking)	17a	Who will be blinded after assignment to interventions (eg, trial participants, care providers, outcome assessors, data analysts), and how	N/A
	17b	If blinded, circumstances under which unblinding is permissible, and procedure for revealing a participant's allocated intervention during the trial	N/A

Methods: Data collection, management, and analysis

Data collection methods	18a	Plans for assessment and collection of outcome, baseline, and other trial data, including any related processes to promote data quality (eg, duplicate measurements, training of assessors) and a description of study instruments (eg, questionnaires, laboratory tests) along with their reliability and validity, if known. Reference to where data collection forms can be found, if not in the protocol	8, 11-16
	18b	Plans to promote participant retention and complete follow-up, including list of any outcome data to be collected for participants who discontinue or deviate from intervention protocols	12

Data management	19	Plans for data entry, coding, security, and storage, including any related processes to promote data quality (eg, double data entry; range checks for data values). Reference to where details of data management procedures can be found, if not in the protocol	17
Statistical methods	20a	Statistical methods for analysing primary and secondary outcomes. Reference to where other details of the statistical analysis plan can be found, if not in the protocol	14-16
	20b	Methods for any additional analyses (eg, subgroup and adjusted analyses)	14-16
	20c	Definition of analysis population relating to protocol non-adherence (eg, as randomised analysis), and any statistical methods to handle missing data (eg, multiple imputation)	14
**Methods: Monitoring**			
Data monitoring	21a	Composition of data monitoring committee (DMC); summary of its role and reporting structure; statement of whether it is independent from the sponsor and competing interests; and reference to where further details about its charter can be found, if not in the protocol. Alternatively, an explanation of why a DMC is not needed	N/A
	21b	Description of any interim analyses and stopping guidelines, including who will have access to these interim results and make the final decision to terminate the trial	N/A
Harms	22	Plans for collecting, assessing, reporting, and managing solicited and spontaneously reported adverse events and other unintended effects of trial interventions or trial conduct	N/A
Auditing	23	Frequency and procedures for auditing trial conduct, if any, and whether the process will be independent from investigators and the sponsor	N/A
**Ethics and dissemination**			
Research ethics approval	24	Plans for seeking research ethics committee/institutional review board (REC/IRB) approval	2, 17
Protocol amendments	25	Plans for communicating important protocol modifications (eg, changes to eligibility criteria, outcomes, analyses) to relevant parties (eg, investigators, REC/IRBs, trial participants, trial registries, journals, regulators)	17

For peer review only - http://bmjopen.bmj.com/site/about/guidelines.xhtml

Consent or assent	26a	Who will obtain informed consent or assent from potential trial participants or authorised surrogates, and how (see Item 32)	12
	26b	Additional consent provisions for collection and use of participant data and biological specimens in ancillary studies, if applicable	N/A
Confidentiality	27	How personal information about potential and enrolled participants will be collected, shared, and maintained in order to protect confidentiality before, during, and after the trial	suppl. material #2
Declaration of interests	28	Financial and other competing interests for principal investigators for the overall trial and each study site	20
Access to data	29	Statement of who will have access to the final trial dataset, and disclosure of contractual agreements that limit such access for investigators	17
Ancillary and post-trial care	30	Provisions, if any, for ancillary and post-trial care, and for compensation to those who suffer harm from trial participation	N/A
Dissemination policy	31a	Plans for investigators and sponsor to communicate trial results to participants, healthcare professionals, the public, and other relevant groups (eg, via publication, reporting in results databases, or other data sharing arrangements), including any publication restrictions	17
	31b	Authorship eligibility guidelines and any intended use of professional writers	17
	31c	Plans, if any, for granting public access to the full protocol, participant-level dataset, and statistical code	17
**Appendices**			
Informed consent materials	32	Model consent form and other related documentation given to participants and authorised surrogates	12, suppl. material #2
Biological specimens	33	Plans for collection, laboratory evaluation, and storage of biological specimens for genetic or molecular analysis in the current trial and for future use in ancillary studies, if applicable	N/A

*It is strongly recommended that this checklist be read in conjunction with the SPIRIT 2013 Explanation & Elaboration for important clarification on the items. Amendments to the protocol should be tracked and dated. The SPIRIT checklist is copyrighted by the SPIRIT Group under the Creative Commons "Attribution-NonCommercial-NoDerivs 3.0 Unported" license.

For peer review only - http://bmjopen.bmj.com/site/about/guidelines.xhtml

Supplementary File 2 – Participant information sheet and consent form for quantitative (p. 2-3) and qualitative data collection (p.4-5) (English version – Example)

For peer review only

For peer review only - http://bmjopen.bmj.com/site/about/guidelines.xhtml

Magnet4Europe - Improving Mental Health and Wellbeing in the Health Care Workplace

Dear colleague,
you have been invited to take part in a research study that aims to improve mental health and wellbeing among health professionals in Europe. The project is funded under the European Union's Horizon 2020 Research and Innovation programme from 2020 to 2023 (Grant Agreement 848031). The study will be conducted in compliance with your country's ethics guidelines for scientific studies on individuals. Your participation will neither involve costs nor reimbursements. You can withdraw from the study at any time, without giving a reason. Before you decide whether to take part it is important for you to understand why the research is being done and what it will involve. Please take your time to read the following information carefully and to decide whether you wish to take part or not. Ask us if there is anything that is not clear or if you would like to receive more information. For questions, please reach out to the Magnet4Europe project coordinator prof. dr. Walter Sermeus at walter.sermeus@kuleuven.be.

What is the purpose of the study?

Mental health and wellbeing are among the highest priorities of the public health agenda in the European Union (EU). A large-scale European study of hospital work conditions and associated nurse and patient outcomes revealed high rates of job dissatisfaction and burnout, with burnout rates varying between 10% and 78%. Physician burnout rates ranging from 25% to 60% have been reported, varying across organizations and medical specialties. Magnet4Europe aims to redesign clinical environments in sixty hospitals in five European countries (Belgium, United Kingdom, Germany, Ireland, and Sweden) to promote the mental health and wellbeing of health professionals. Magnet4Europe will implement an evidence-based intervention based on the successful Magnet Recognition Program®, a voluntary hospital designation for nursing care excellence by the American Nurses Credentialing Center. Countless studies have shown that Magnet-recognized hospitals have lower health professional burnout and safer patient care suggesting that the Magnet journey is an intervention that results in positive changes Magnet4Europe seeks to achieve.

How does this research study work?

This research study will be carried out between January 2020 and December 2023 in adult inpatient units. The intervention itself will officially start in September 2020 in participating European hospitals and will target the five overarching principles of the Magnet® concept: structural empowerment of clinical staff, transformational leadership, exemplary and evidence-based professional practice, new knowledge, innovations, and improvements, and empirical outcomes. These principles will serve as a continuous feedback loop as to whether organizational changes are producing the intended outcomes for staff and patients. The Magnet® blueprint provides definitions of the principles and gives examples of evidence-based indicators that reflect progress toward achieving them. To facilitate the implementation process, each participating European hospital receives support by one to one twinning with an experienced Magnet recognized hospital including annual on-site visits and by the creation of a critical mass of participating hospitals drawing public interest and promoting innovation, and replication. Before the intervention period officially starts, eligible participants will receive a link to the online platform Meplis Care Monitor via mail in May/June 2020. Following the link, they will be able to register first and then to complete a questionnaire. Within the scope of this questionnaire participants will be asked about various aspects of their work environment (work relations, job satisfaction, perception of the quality of care and workload) and mental health. In the course of the study, this procedure will be repeated annually for a total of 4 measurement occasions. In addition to the surveys, occasional focus groups (i.e., moderated interactive group discussions) will take place in a selection of hospitals between July 2021 and September 2023. The focus groups aim to understand clinicians' personal views and thoughts on the process of the intervention and to identify barriers and facilitators to implementation of the intervention.

Why have I been invited and am I eligible?

60 hospitals across the five European countries being involved have agreed to participate in this study. Within each participating hospital, all adult inpatient units including ICU and ER have been selected. You are eligible to participate if you (1) have direct patient contact and (2) meet the minimum standards of Directive 2013/55/EU amending Directive 2005/35/EC on the recognition of professional qualifications. You are invited to participate in this study, because you belong to the clinical staff of one of these departments and meet the selection criteria.

What will my participation in the study involve?

In order to participate in the survey, we ask you to register on Meplis Care Monitor after receiving your activation link. Following the instructions given in the questionnaire, we ask you to answer a number of questions about your perception of your working environment: the relationship with the doctors, the workload and staffing of the ward, any intention (if any) to leave the profession, patient safety, etc. The questionnaire takes approx. 20 minutes to complete; it should be completed within two weeks of receiving this invitation. Once you have successfully created an account and participated in the first survey, you will receive an automatically generated mail by Meplis Care Monitor for three more times, inviting you to participate in the annual survey. If you are new to this hospital and did not participate in the last measurement occasion(s) please reach out to the person instructed by the hospital in order to invite you to the Meplis Care Monitor platform.

Do I have to take part?

It is up to you to decide whether or not to take part. If you do decide to take part you will be given this information sheet to keep. After signing up at Meplis Care Monitor, you will be asked to express your consent before completing the questionnaire. You will not be able to engage in the survey prior to providing an informed consent. If you decide to take part you are still free to withdraw at any time and without giving a reason. The choice that you make will have no bearing on your job or on any work-related evaluations or reports. You may change your mind later and stop participating even if you agreed earlier.

Are there any benefits for me in joining the study?

You have no direct benefit from participation and will not be compensated for your participation (there are no costs or compensation associated with participation). However, we hope that your participation in the study will help us to gain more insight on how you perceive hospital care and the demands that are placed on clinical staff. By doing so, you can help to improve the working environment and safety of patient care.

Are there any risks for me in joining the study?

There are no disadvantages associated with participation. Neither the research team nor the hospital will know whether you have participated in the study or not. A possible refusal to participate in the study will have no adverse effect on your employment at this hospital. Nor will your answers have any effect on your participation. However, you may feel uncomfortable reading the information and answering the questions.

Who has approved this study?

The research project was submitted to the central and/or local ethics committees within your country for approval. This authority has not raised any objections to the design of the study.

Will information about me be kept confidentially?

If you agree to participate in this study, a unique code will be stored along with your answers. Nobody but you can make the connection between your identity, this code, and your answers. Your data will be processed in accordance with Regulation (EU) 2016/679 of 27 April 2016 on the protection of individuals with regard to the processing of personal data and on the free movement of such data, which entered into force on 25 May 2018. If the results of the study are published, your anonymity is guaranteed. The final results of the analyses will be published on the Magnet4Europe website (www.magnet4europe.eu) and will be publicly accessible. Your identity will not be revealed in any of these cases. Individual answers will not be published and also research reports will not include information on clinical staff or individual hospitals. Neither the hospital nor your superiors will have access to individual answers.

For peer review only - http://bmjopen.bmj.com/site/about/guidelines.xhtml

Consent to participate in the study

	Mark 'Yes' to continue
I hereby declare that I have been informed in an understandable manner about the nature, method and purpose of this study.	Yes
I agree to participate in this scientific research.	Yes
I am aware that participation in these studies does not entail any additional costs and that there is no financial benefit.	Yes
I am aware that I can withdraw at any time up to the moment that the data is kept in the database without having to make a statement and without this in any way affecting me.	Yes
I understand that my data is collected and registered confidentially, and that the principal investigator guarantees its confidentiality.	Yes

Date	Signature

For peer review only

Magnet4Europe - Improving Mental Health and Wellbeing in the Health Care Workplace

Dear Sir/Madam,

you have been invited to take part in a research study that aims to improve mental health and wellbeing among health professionals in Europe. The project is funded under the European Union's Horizon 2020 Research and Innovation programme from 2020 to 2023 (Grant Agreement 848031). The study will be conducted in compliance with your country's ethics guidelines for scientific studies on individuals. Your participation will neither involve costs nor reimbursements. You can withdraw from the study at any time, without giving a reason. Before you decide whether to take part it is important for you to understand why the research is being done and what it will involve. Please take your time to read the following information carefully and to decide whether you wish to take part or not. Ask us if there is anything that is not clear or if you would like to receive more information. For questions, please reach out to the Magnet4Europe project coordinator prof. dr. Walter Sermeus at walter.sermeus@kuleuven.be

What is the purpose of the study?

Mental health and wellbeing are among the highest priorities of the public health agenda in the European Union (EU). A large-scale European study of hospital work conditions and associated nurse and patient outcomes revealed high rates of job dissatisfaction and burnout, with burnout rates varying between 10% and 78%. Physician burnout rates ranging from 25% to 60% have been reported, varying across organizations and medical specialties. Magnet4Europe aims to redesign clinical environments in sixty hospitals in six European countries (Belgium, United Kingdom, Germany, Ireland, Norway, and Sweden) to promote the mental health and wellbeing of health professionals. Magnet4Europe will implement an evidence-based intervention based on the successful Magnet Recognition Program®, a voluntary hospital designation for nursing care excellence by the American Nurses Credentialing Center. Countless studies have shown that Magnet-recognized hospitals have lower health professional burnout and safer patient care suggesting that the Magnet journey is an intervention that results in positive changes Magnet4Europe seeks to achieve.

How does this research study work?

This research study will be carried out between January 2020 and December 2023 in adult inpatient units. The intervention itself will officially start in September 2020 in participating European hospitals and will target the five overarching principles of the Magnet® concept: structural empowerment of clinical staff, transformational leadership, exemplary and evidence-based professional practice, new knowledge, innovations, and improvements, and empirical outcomes. These principles will serve as a continuous feedback loop as to whether organizational changes are producing the intended outcomes for staff and patients. The Magnet® blueprint provides definitions of the principles and gives examples of evidence-based indicators that reflect progress toward achieving them. To facilitate the implementation process, each participating European hospital receives support by one to one twinning with an experienced Magnet recognized hospital including annual on-site visits and by the creation of a critical mass of participating hospitals drawing public interest and promoting innovation, and replication.

You are asked to participate in this study because your workplace belongs to one of the hospitals participating in the Magnet4Europe project. A selection of nurses and doctors working at the participating hospitals will be asked to participate in focus groups (i.e moderated interactive group discussions) or individual interviews. Focus groups and interviews will be used to investigate experiences and perceptions of the implementation process and to identify factors that hinder or facilitate the implementation of the intervention.

Why have I been invited and am I eligible?

60 hospitals across the six European countries being involved have agreed to participate in this study. Within each participating hospital, all adult inpatient units including ICU and ER have been selected. You are eligible to participate if you (1) have direct patient contact and (2) meet the minimum standards of Directive 2013/55/EU amending Directive 2005/35/EC on the recognition of professional qualifications.

You are invited to participate in this study, because you belong to the clinical staff of one of these departments and meet the selection criteria.

What will my participation in the study involve?

We want to investigate perceptions of the clinical work environment and to monitor the mental health and well-being of healthcare professionals among the hospitals participating in Magnet4Europe. Participation means that you will participate in a focus group discussion (i.e together with about 2-7 other people from your hospital) or in an individual interview that will be about your experiences and experiences of the Magnet4Europe project at your workplace. The interviews and the focus group discussions are estimated to take about 30–60 minutes and 90–120 minutes respectively. The interviews and focus group discussions are conducted in person or via virtual means. The interviews and focus group discussions will be recorded through audio recording so that they can later be transcribed verbatim and analyzed.

Do I have to take part?

Your participation is voluntary and you can choose to cancel the participation at any time. If you choose not to participate or want to cancel your participation, you do not need to state why, nor will it affect your work or employment in the future. If you wish to cancel your participation, you should contact the person responsible for the study (see contact details at the end of the information letter).

Are there any benefits or risks for me to participate in the study?

You have no direct benefit from participation and will not be compensated for your participation (there are no costs or compensation associated with participation). However, we hope that your participation in the study will help us to gain more insight on how you perceive the implementation process in your hospital. By doing so, you can help to improve the working environment and safety of patient care. There are no disadvantages associated with participation. You should be aware, however, that the questions discussed during the interviews and focus groups can elicite discomfort or other emotional responses. If you feel that you need to talk to someone after the interviews, we recommend that you contact occupational health care expert at your hospital.

Will information about me be kept confidentially?

The research being done in the community may draw attention and if you participate you may be asked questions by other people in the community. We will not be sharing any information about you to anyone outside of the research team. If you participate in a focus group discussion, we will ask you and others in the group not to talk to people outside the group about what was said in the group. We will, in other words, ask each of you to keep what was said in the group confidential. You should know, however, that we cannot stop or prevent participants who were in the group from sharing things that should be confidential.

Who has approved this study?

The research project was submitted to the central and/or local ethics committees within your country for approval. This authority has not raised any objections to the design of the study.

How do I get information about the results of the study?

The results of the study will be compiled at the group level and reported in such a way that no individual individuals will be able to be identified. The results of the study will be published in scientific journals and research reports in such a way that individual answers, individuals, clinics or hospitals cannot be identified. Results from the study will also be published on Magnet4Europe's website, www.magnet4europe.eu, but will not contain information that allows individuals or hospitals to be identified.

Feel free to contact us if you have any questions or if you would like more information.

For peer review only - http://bmjopen.bmj.com/site/about/guidelines.xhtml

Consent to participate in the study

I have been given oral and written information about the study and have had the opportunity to ask questions. I may retain the written information.

☐ I agree to participate in an interview or focus group discussion within the study Magnet4Europe

☐ I agree that information about me is processed in the manner described in the research information.

Date	Signature

For peer review only
